# Global distribution and multidimensional risk assessment of brucellosis in humans, livestock, and wildlife

**DOI:** 10.3389/fmicb.2026.1844035

**Published:** 2026-07-06

**Authors:** Jianxiang Qiu, Ruihao Peng, Wei Kan, Zhixin Fang, Yiyu Chen

**Affiliations:** 1 The Department of Medical Administration, The Affiliated Guangdong Second Provincial General Hospital of Jinan University, Guangzhou, China; 2 NMPA Key Laboratory for Quality Monitoring and Evaluation of Vaccines and Biological Products, One Health Center of Excellence for Research and Training, School of Public Health, Sun Yat-Sen University, Guangzhou, China; 3 School of Public Health, Guangdong Medical University, Dongguan, China; 4 Animal Disease Prevention and Control Center in Qinghai Province, Xining, China

**Keywords:** brucellosis, ecological niche modeling, global risk assessment, One Health surveillance, wildlife–livestock interface

## Abstract

Brucellosis is a globally distributed zoonotic disease transmitted among livestock, wildlife, and humans, posing a substantial global public health challenge. However, the global distribution of brucellosis risk and its impacts across the human–livestock–wildlife interface remain insufficiently characterized. In this study, we developed a global ecological risk assessment framework to characterize the spatial distribution of brucellosis and its major environmental and anthropogenic drivers. Using a comprehensive global database of *Brucella* spp. occurrence records, we applied niche modeling approaches to predict disease suitability and quantify populations and animal species potentially exposed to brucellosis risk. The model demonstrated strong predictive performance (AUC = 0.901) and identified Asia as the primary global hotspot, with additional high-risk areas located in eastern and western Africa. Population density, land cover, pasture, urban water, soil characteristics, and minimum temperature of the coldest month were identified as the major predictors shaping the global distribution of brucellosis risk. We estimated that approximately 58.3 to 61.5 million people and 1.03 to 1.22 billion livestock, including goats, sheep, cattle, and buffaloes, reside within high-risk areas. Furthermore, 5,212 mammal species showed spatial overlap with predicted brucellosis risk zones, including 214 threatened wild artiodactyl species. Several threatened species, such as *Panthera tigris*, *Panthera pardus*, *Rusa unicolor*, and *Capricornis sumatraensis*, were found to have substantial overlap with high-risk areas, suggesting potential exposure at the livestock–wildlife interface. These findings reveal the extensive ecological footprint of brucellosis across human, livestock, and wildlife populations and highlight the importance of integrating wildlife conservation into brucellosis surveillance and control programs. The resulting global risk maps provide a valuable tool for One Health-based surveillance, targeted intervention, and conservation planning.

## 1 Introduction

Brucellosis remains an important yet neglected zoonotic disease worldwide. It is caused by *Brucella* spp., a group of the facultative intracellular Gram-negative bacteria capable of infecting a wide range of domestic animals, wildlife species, and humans. This disease is distributed in more than 170 counties and regions, and is estimated to affect approximately 1/7 ~ 1/6 of the global population, with the majority occurring in impoverished rural areas (Liang et al., 2020). Globally, more than 500,000 human brucellosis cases are reported each year, with the majority occurring in developing regions of Asia, Africa, and South and Central America (Pappas et al., 2006; Dean et al., 2012; Laine et al., 2023). Clinically, the symptoms of brucellosis are highly non-specific, frequently leading to misdiagnosis, missed diagnoses, or delayed treatment, thereby increasing the risk of complications and chronic disease (Zheng et al., 2018). Although mortality from brucellosis is low, its persistent incidence and widespread prevalence contribute to substantial disability, labor loss, and public health burden.

The transmission dynamics of brucellosis are strongly shaped by ecological interactions at the wildlife-domestic livestock-human interface, often involving complex pathogen spillover events (Hassell et al., 2017; Carlson et al., 2019). Different *Brucella* species exhibit varying host preferences. Among the domestic livestock animals, *Brucella melitensis* primarily infects goats and sheep, *Brucella abortus* mainly infects cattle, and *Brucella suis* is predominantly associated with swine (Godfroid et al., 2013; Gonzalez-Espinoza et al., 2021; Kurmanov et al., 2022). Therefore, livestock may serve as intermediate or amplifier hosts, spilling over the pathogens into humans (Godfroid, 2017). Meanwhile, wildlife species may also participate in the epidemiology of brucellosis as spillover hosts, maintenance hosts, or ecological reservoirs that contribute to pathogen circulation among animal populations and humans (Kamath et al., 2016). Furthermore, the *Brucella* spp. can persist in the contaminated environment substrates, including soil, water, and vegetation, creating opportunities for indirect transmission through livestock grazing, wildlife migration, and environmental exposure (Zhao et al., 2019). These complex transmission pathways highlight the importance of adopting a ‘One Health’ concept and approach that integrates human, animal, and environmental health perspectives for effective brucellosis surveillance and control (Godfroid et al., 2014).

In recent years, ecological niche modeling (ENM) has emerged as an effective tool for predicting the global distribution and risk of emerging and re-emerging infectious diseases, such as *anthrax*, *COVID-19*, *peste des petits ruminants virus*, and *Serratia marcescen* (Assefa et al., 2021; Carlson et al., 2019; Coro, 2020; Shana et al., 2023). By integrating environmental, climatic, demographic, and biological variables, ENM can identify environmentally suitable regions for pathogen persistence and transmission, thereby supporting risk assessment and disease surveillance planning. However, comprehensive ecological niche modeling studies of brucellosis remain limited. The absence of a unified global risk map hampers the ability of many countries (particularly low-income countries) to allocate their limited vaccination resources and establish evidence-based zoonotic disease surveillance systems. In addition, the ecological exposure of endangered wildlife species to brucellosis remains poorly characterized at the global scale, which may lead to an underestimation of its potential impacts on wildlife conservation and population recovery.

Although there have been numerous localized studies and ongoing global discussions of brucellosis (Laine et al., 2023), several important knowledge gaps remain. First, there is currently no unified global risk map integrating the human-livestock-wild animal interfaces. Second, quantitative assessment of potential brucellosis exposure among wild artiodactyl species at the global scale remains lacking. Third, operational risk classification framework capable of guiding evidence-based resource allocation for international organizations such as the World Organisation for Animal Health (WOAH) and the Food and Agriculture Organization (FAO) are still insufficiently developed. Previous research has rarely employed ecological niche models to evaluate regional suitability for the disease, and attempts to generate globally integrated risk assessments have been limited (Jia and Joyner, 2015). Given recent socioeconomic shifts and environmental change, it is imperative to update global brucellosis risk assessments using ecological niche modeling approaches. Which is crucial for enhancing brucellosis prevention and control measures.

To address current knowledge gaps in the global epidemiology of brucellosis, this study aimed to characterize the spatial distribution of brucellosis risk worldwide, identify the major ecological and anthropogenic factors associated with disease distribution, and evaluate potential exposure patterns across wildlife, livestock, and human populations across different ecological interfaces. To achieve these objectives, we compiled and integrated global occurrence data from scientific literature and multiple disease surveillance databases and constructed a global ecological niche model for *Brucella* spp. This framework provides a basis for improving risk assessment, supporting One Health surveillance, and informing future brucellosis prevention and control strategies.

## 2 Materials and methods

### 2.1 Occurrence data and environmental predictors

We assembled a global occurrence database for *Brucella melitensis*, *Brucella abortus*, and *Brucella suis* out of a combination of occurrence data collected in the field by the authors or their extended team of collaborators, national passive surveillance and WOAH, online records from ProMed Mail, and georeferenced records or digitized maps from peer-reviewed publications documenting *Brucella* outbreaks. The occurrence records covered the period from 1975 to 2023. The WOAH and ProMed databases provided occurrence records containing geographic coordinates or country−/region-level location information. For records extracted from published literature, geographic coordinates reported directly in the studies were preferentially used. When coordinates were unavailable, locations were geocoded based on place descriptions using Google Earth and the GeoNames database. To minimize potential sampling bias and reduce the effects of spatial autocorrelation, duplicate records from the same geographic area were removed, and occurrence points were spatially filtered using the “Spatially Rarefy Occurrence Data” tool implemented in SDM Toolbox v1.1c. Minimum distance thresholds of 10, 50, 100, and 150 km were evaluated, and a threshold of 100 km was selected as the optimal filtering distance because it effectively reduced spatial clustering while maintaining adequate sample representation. After spatial filtering and data cleaning, a total of 1,178 occurrence points were retained for subsequent analyses. The host datasets, climate, terrain, vegetation, and human impact were used to construct the model in this study (Table 1). We extracted climatic predictor variables from the WorldClim version 1.4 with data from 1950 to 2000 at 30 arc-second resolution.^1^ Nineteen bioclimatic variables were included in the analysis. Although the occurrence records spanned multiple decades, WorldClim v1.4 represents one of the most widely used and standardized global climate datasets for ecological niche modeling, and the temporal differences between occurrence records and climatic baselines were considered unlikely to substantially affect large-scale suitability predictions. Terrain-related variables include elevation and distance to the river. Host-related variables include sheep density, goat density, and cattle density. Anthropogenic variables includes population density, distance to road, nighttime lights, urban–rural catchment areas, croplands, and pastures. Vegetation-related variables includes land cover and soils. All spatial data were preprocessed and calculated in ArcGIS 10.6 and projected in UTM-WGS-1984 with standard settings or resampling to 30 arc seconds.

**Table 1:** The variable information data layer, source, and variable of the prediction model.

Layer	Source	Variable/proxy
Climate		
Bioclimatic 1–19	http://worldclim.org/version1	Annual trends, seasonality, extreme or limiting environmental variables
Terrain		
Elevation	https://earthexplorer.usgs.gov	Climbing distance
Distance to river	https://www.hydrosheds.org/	Water source
Human impact		
Population Density	https://www.worldpop.org/wprfpms/	Human-Animal interaction
Distance to road	http://download.geofabrik.de/	Human-Animal interaction
Vegetation		
Land cover	https://maps.elie.ucl.ac.be/CCI/viewer/	Animal food and refuge
Soils	https://soilgrids.org/	Vegetation nutrients
Economic		
Nighttime Lights	https://www.earthdata.nasa.gov/learn/backgrounders/nighttime-lights	Economic development-disease interaction
Urban, rural, and catchment areas	https://data.apps.fao.org/map/gsrv/gsrv1/	Economic development-disease interaction
Living habits		
Croplands	https://data.apps.fao.org/catalog/iso/9dc31512-a438-4b59-acfd-72830fbd6943?	Living habits-disease interaction
Pastures	https://gibs.earthdata.nasa.gov/wms/epsg4326/best/wms.cgi?	Living habits-disease interaction
Host		
Sheep density	https://livestockdata.org/contributor/gridded-livestock-world-glw3	Host-disease interaction
Goat density	https://livestockdata.org/contributor/gridded-livestock-world-glw3	Host-disease interaction
Cattle density	https://livestockdata.org/contributor/gridded-livestock-world-glw3	Host-disease interaction
Buffalo density	https://livestockdata.org/contributor/gridded-livestock-world-glw3	Host-disease interaction

### 2.2 Distribution modeling

The MaxEnt model is currently recognized as an effective ecological niche modeling approach for predicting species distributions and infectious disease risk, particularly when only presence data are available (Gesink Law et al., 2006; Matsane et al., 2025; Elith et al., 2011; Phillips et al., 2006). Previous studies have successfully applied MaxEnt to the risk assessment and spatial prediction of multiple infectious diseases (Peterson, 2006; Carlson et al., 2019; Gao et al., 2023). In this study, the filtered occurrence records of brucellosis (*Brucella. melitensis*, *Brucella. abortus*, and *Brucella. suis*) and a suite of environmental predictor variables were used to construct a global ecological suitability model. To minimize spatial autocorrelation and sampling bias, occurrence records were spatially filtered using the “Spatially Rarefy Occurrence Data” tool in SDM Toolbox v1.1c implemented in ArcGIS 10.6 (Brown et al., 2017), as described above. Sampling bias was further reduced through spatial thinning of occurrence data prior to model fitting.

Multicollinearity among predictor variables was addressed through a combination of principal component analysis (PCA) for climatic variables and variance inflation factor (VIF) diagnostics for the final variable set. Climatic predictor variables were first screened using PCA, and components with eigenvalues greater than 1.0 were retained according to the scree plot criterion and broken-stick stopping rule. Non-climatic variables (including land use, socioeconomic factors, and host density) were retained based on ecological relevance and collinearity filtering. Variables with low contribution or unstable response curves were iteratively removed to improve model stability and reduce overfitting.

MaxEnt modeling was implemented using MaxEnt version 3.3.4 software.^2^ Model settings included automatic feature selection, a regularization multiplier of 1.0, a maximum of 5,000 iterations, and a convergence threshold of 10^−5^. A total of 10,000 background points were randomly generated to characterize environmental availability within the study region. Occurrence data were partitioned into training (70%) and testing (30%) datasets, and model robustness was evaluated using 10 bootstrap replicates with random seed resampling to ensure independent data splits across runs.

**Figure 1:**
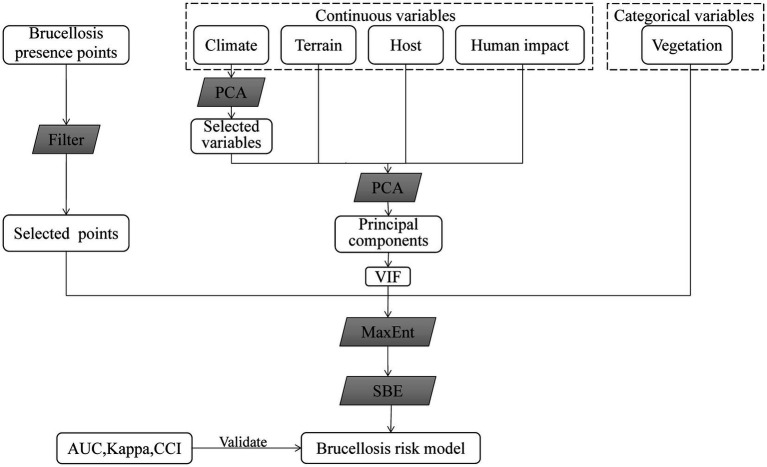
Flowchart of brucellosis risk model construction.

Different combinations of parameters were tested to optimize model performance and minimize overfitting, model instability, and omission errors. Model selection was guided using ENMTools (version 1.4.4) together with the Akaike information criterion corrected for small sample sizes (AICc) (Warren and Seifert, 2011). Model performance was evaluated using both threshold-independent and threshold-dependent metrics. The area under the receiver operating characteristic (ROC) curve (AUC) was used as a primary threshold-independent measure, with values interpreted as excellent (0.9 ~ 1), good (0.8 ~ 0.9), fair (0.7 ~ 0.8), and poor (<0.7). However, recognizing the limitation of AUC, additional evaluation metrics were also applied, including the true skill statistic (TSS) and omission rate at the 10% threshold. The final model achieved an AUC of 0.87, a TSS of 0.78, and an omission rate of 0.12, indicating good predictive performance and acceptable model reliability. In addition, the 95% confidence interval of AUC derived from bootstrap replicates further supported model stability. Finally, occurrence records were projected onto the model output maps for spatial validation. Refer to Figure 1 for the flowchart of the brucellosis risk model construction.

### 2.3 Estimation of livestock populations at risk

We estimated the vulnerability of human and livestock populations to brucellosis by overlaying the predicted brucellosis suitability maps with global population and livestock density datasets, according to the methodology reported previously (Arotolu et al., 2023; Han et al., 2024). To improve methodological clarity and avoid conceptual ambiguity, we explicitly distinguished between continuous model outputs, threshold-based suitability classification, and visualization-oriented categorical mapping.

The continuous suitability outputs from the MaxEnt model were converted into binary suitable/unsuitable areas using the equilibrium threshold. This threshold represents a commonly used criterion in species distribution modeling that balances omission and commission errors and is often applied in ecological and epidemiological studies for delineating potential suitable habitats. To evaluate the sensitivity of results to threshold selection, we conducted a threshold-based sensitivity analysis by applying values 5% below and 5% above the MaxEnt equilibrium threshold, following the approach described previously (Carlson et al., 2019). The resulting lower and upper estimates were used to provide a plausible range of livestock populations potentially exposed to brucellosis risk. These ranges should not be interpreted as statistical confidence intervals, but are derived from a threshold-based sensitivity analysis reflecting the effect of equilibrium threshold variation on exposure estimates.

To further facilitate spatial visualization, suitability values were additionally classified into categorical risk levels (low, medium, and high risk) using the Jenks natural breaks (natural breaks optimization) method. Importantly, this classification was applied only for map visualization and descriptive stratification and was not used to define or modify the primary high-risk threshold derived from the MaxEnt model. The Jenks method was selected because it minimizes within-class variance and maximizes between-class variance, and it is widely applied in geographical and epidemiological mapping for data-driven classification purposes.

To estimate population exposure, the classified risk layers were spatially intersected with global human population density data and livestock distribution datasets, including goats, sheep, cattle, and buffalo at approximately 1 km × 1 km spatial resolution. Analyses were conducted at the continental scale by overlaying risk layers with administrative boundaries to quantify population and livestock numbers within predicted brucellosis risk zones. The resulting estimates provide a spatially explicit quantification of potential exposure for both human and animal populations across different regions.

### 2.4 Define the area of wildlife at risk

We evaluated the overlap between the range map of each existing ungulates and the conservation status of the International Union for Conservation of Nature (IUCN) (Artiodactyla), reduced from all mammals in IUCN (see Supplementary material). To determine threatened protected species at risk of brucellosis outbreaks, we extracted high-risk areas for brucellosis and covered them with even-toed and odd-toed ungulates protected by the International Union for Conservation of Nature (IUCN), referencing previous studies on preserving of Artiodactyla. We have compiled a list of Artiodactyla using data from the International Union for Conservation of Nature’s Red List and classified them as Near Threatened, Vulnerable, and Endangered - critically endangered and extinct. At the same time, we cross the selected range of candidate species with high risk of brucellosis and calculate the areas where different wild animals are at high risk below the risk of brucellosis. Meanwhile, classifying and sequencing the wild species with a higher risk of brucellosis based on the size of the affected area.

## 3 Results

**Figure 2:**
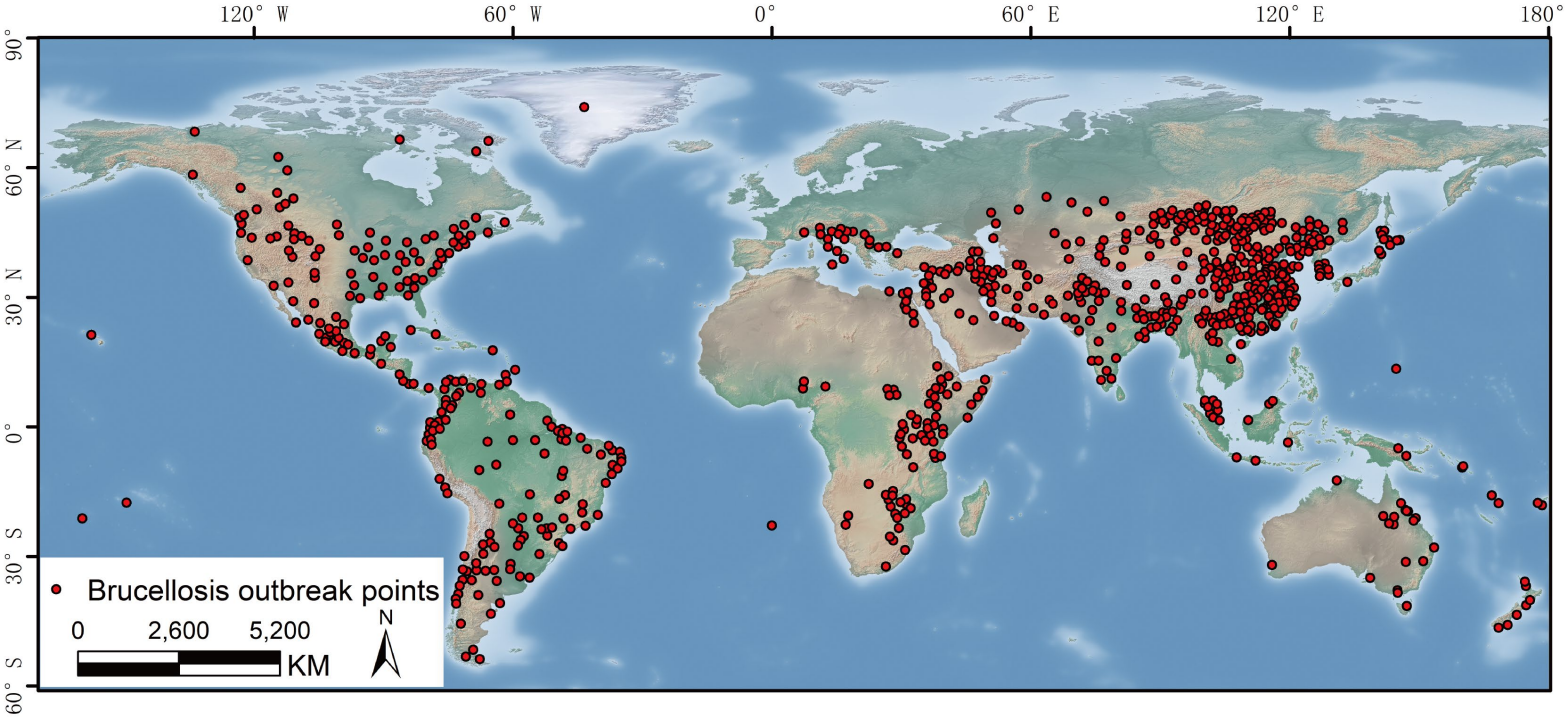
Global distribution of outbreaks by country and geographic locations of brucellosis events. This map was made in ArcGIS 10.6. The boundary was obtained from Natural Earth (http://www.naturalearthdata.com/), which is a schematic line illustrating the relative position of each country and should not be re-used or misinterpreted for any political reason.

Our model performed very well on validation data, AUC, SD, Kappa, and CCI are 0.901, 0.003, 0.80, and 0.81, respectively, indicating the best prediction, which also validated the model’s robustness. After spatial filtering, 795 geographic locations reporting brucellosis caused by *Brucella melitensis*, *Brucella abortus*, and *Brucella suis* were retained, with all occurrence records located at least 100 km apart from one another (Figure 2).

**Figure 3:**
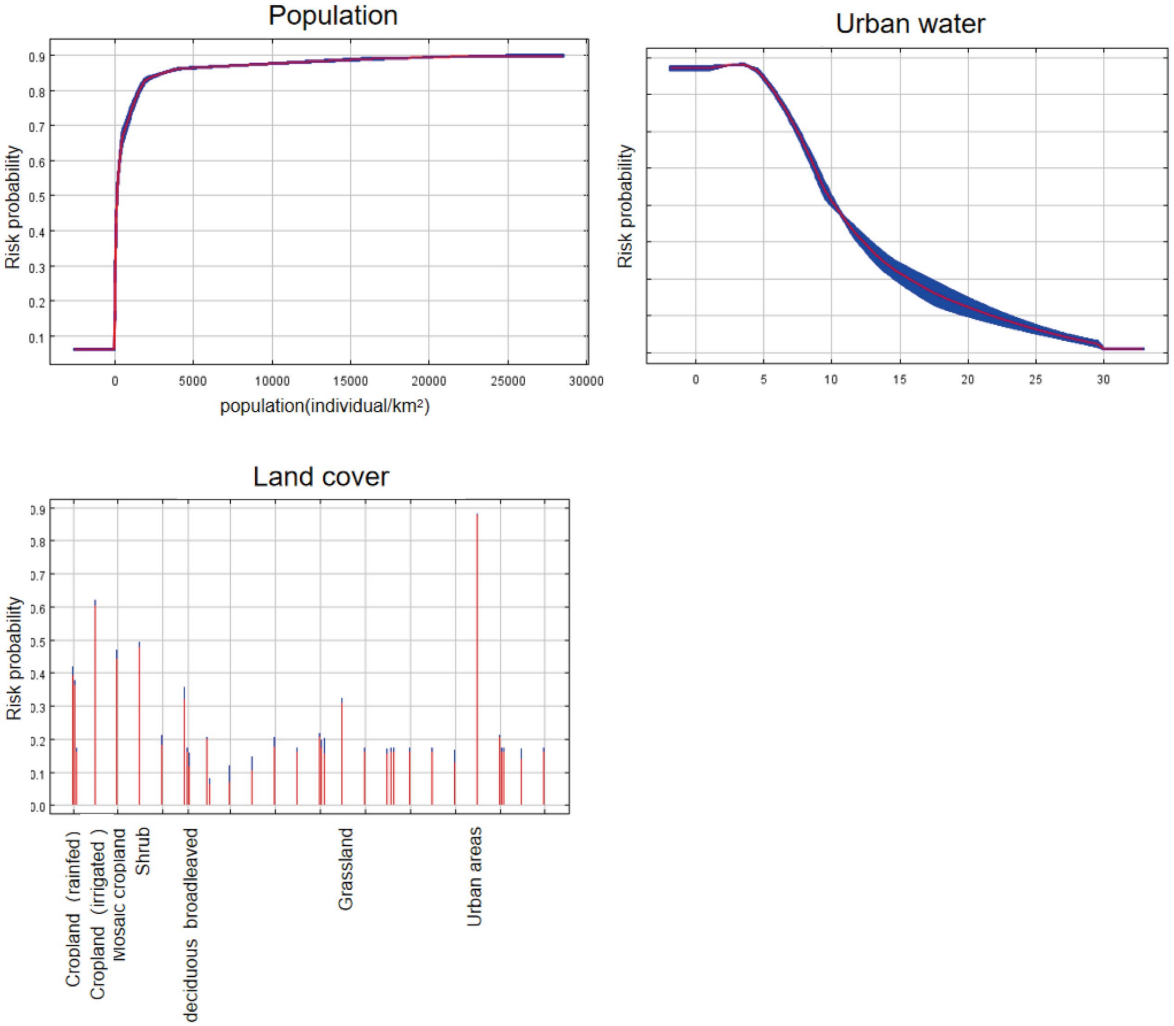
The response curves of the different predictors. The curves show the mean response (red) and the mean standard deviation (blue).

The PCA was applied to the initial set of 19 meteorological factors, identifying key components for dimensionality reduction. And the MaxEnt modeling, incorporating both meteorological and non-meteorological factors, facilitated the iterative removal of predictors exhibiting the lowest permutation importance. Further refinement was achieved by eliminating variables associated with high standard deviation (SD) in their response curves, which yielded a final optimized set of predictive variables. Finally, we obtained that population density, urban water, land cover, soil, pasture, and Bio11 are key factors affecting their occurrence, and the relative contributions of each predictor are shown in Table 2. The VIF value in the predictive factors is 1.325–4.560, which meets the low multicollinearity criterion (<10). The response curves of different predictive factors are shown in Figure 3.

**Table 2:** Estimation of relative contributions and the optimum interval of the multiple variants to the final models.

Variant	Contribution%	Permutation importance
Population density	57.1	50.8
Urban–rural catchment areas	13.9	6.1
Land cover	13.1	6.2
Soil	9.9	14.4
pasture	3.6	10.2
Bio11	2.3	12.2

**Figure 4:**
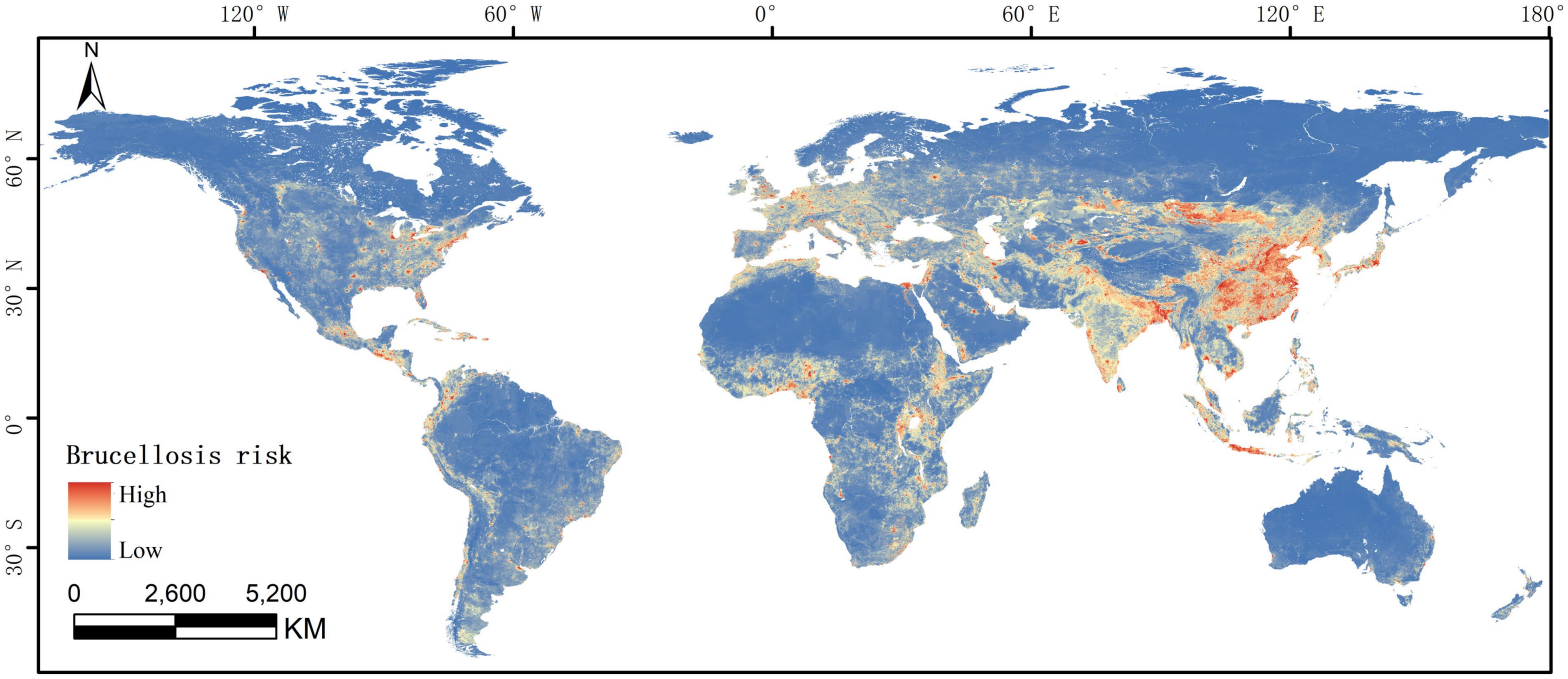
Global distribution of brucellosis suitability was predicted by the MaxEnt model. The map was generated in ArcGIS 10.6 using raster outputs derived from the MaxEnt model.

The predicted high-risk areas by the MaxEnt model are shown in Figure 4. The model identified that high-risk areas for brucellosis are predominantly located in Asia. This region is also known for having the largest number of cattle and sheep being raised globally. In Africa, two distinct high-risk zones were detected in the eastern and western regions. The Americas and Europe, despite having developed economies, standardized animal husbandry management, and high vaccination rates, still exhibit small and scattered high-risk areas.

On a global scale, we have found an estimated number of people (58,291,526.25-61,483,587.33) living in high-risk areas of brucellosis. Among them, Asia has the most affected population. In addition, we have found that livestock (1,027,551,961-1,219,762,242) are at risk of brucellosis in high-risk areas, including sheep (238,339,517 - 293,978,806), goat (364,762,480 - 417,577,064), cattle (334,771,242 - 399,572,703), and buffalo (89,678,722 - 108,633,669). Among them, Asian livestock are the region with the highest number of affected populations, as shown at Table 3.

**Table 3:** Estimated livestock populations at risk under threshold-based sensitivity analysis.

Region	Sheep		Goat		Cattle		Buffaloes	
Threshold scenario^a^	Lower	Upper	Lower	Upper	Lower	Upper	Lower	Upper
Africa	54,004,726	69,721,725	82,634,416	100,932,987	71,669,755	88,946,923	3,214,935	3,254,052
Asia	160,759,557	193,024,969	82,634,416	308,267,164	210,769,263	243,097,627	86,276,190	105,121,545
Europe	14,379,728	18,671,346	2,378,166	3,072,035	19,010,458	25,313,974	92,347	129,670
Northern America	3,801,217	4,850,472	196,212,809	4,035,764	17,330,455	21,302,053	7,525	8,611
Oceania	1,856,159	2,174,323	26,467	43,863	1,002,531	1,463,798	25	33
Southern America	3,538,127	5,535,969	876,204	1,225,247	14,988,777	19,448,326	87,697	119,756
Total	238,339,517	293,978,806	364,762,480	417,577,064	334,771,242	399,572,703	89,678,722	108,633,669

^a^Lower and upper estimates were derived from a sensitivity analysis in which the MaxEnt equilibrium threshold was varied by ±5%, resulting in corresponding changes in the spatial area of predicted suitable habitat used to quantify the number of livestock potentially exposed to brucellosis risk, as described in the Materials and Methods section.

Our results show that 5,625 mammal species are listed in the IUCN. Among them, 5,212 species of mammals have geographical distributions that overlap with the risk areas of brucellosis. For details, please refer to Table 4. There are 322 species of cloven-hoofed animals, of which 214 are threatened by brucellosis, accounting for about 66%. Please refer to attached Table 5. *Mus musculus* (11,527,645.99km^2^), *Vulpes vulpes* (8,459,133.90km^2^), *Panthera pardus* (7,391,504.42km^2^), *Sus scrofa* (7,200,021.43km^2^), *Rattus rattus* (7,028,081.82km^2^), *Canis lupus* (6,242,572.43km^2^), *Arctonyx albogularis* (5,242,413.06km^2^), *Panthera tigris* (4,888,832.34km^2^), *Rattus tanezumi* (4,226,701.05km^2^), *Micromys minutus* (3,976,998.23km^2^), and other animals were most affected by brucellosis. Reference Table 4: *Sus scrofa* (7,200,021.43km^2^), *Rusa unicolor* (2,729,116.89km^2^), *Muntiacus vaginalis* (2,729,116.89km^2^), *Capreolus pygargus* (2,070,177.65km^2^), *Muntiacus reevesi* (1,563,987.44km^2^), *Tragelaphus scriptus* (1,477,672.11km^2^), *Sylvicapra grimmia* (1,423,395.15km^2^), *Capricornis sumatraensis* (1,248,042.83km^2^), *Elaphodus cephalophus* (1,189,053.17km^2^), *Kobus ellipsiprymnus* (1,083,023.71km^2^) has the largest area affected by brucellosis as shown in Table 5.

**Table 4:** Several even-toed mammals designated by the International Union for Conservation of Nature (IUCN) as most affected by *Brucella* risk.

Latin name	IUCN status	Distribution area (km^2^)	Brucellosis overlap (km^2^)	Prevalence by brucellosis
*Mus musculus*	Least Concern (LC)	88,275,170.85	11,527,645.99	0.130587637
*Vulpes vulpes*	Least Concern (LC)	72,397,750.93	8,459,133.90	0.116842496
*Panthera pardus*	Vulnerable (VU)	34,699,649.09	7,391,504.42	0.213013809
*Sus scrofa*	Least Concern (LC)	28,012,632.32	7,200,021.43	0.257027663
*Rattus rattus*	Least Concern (LC)	28,229,929.32	7,028,081.82	0.248958534
*Canis lupus*	Least Concern (LC)	56,009,404.28	6,242,572.43	0.111455791
*Arctonyx albogularis*	Least Concern (LC)	8,420,321.946	5,242,413.06	0.622590572
*Panthera tigris*	Endangered (EN)	11,805,947.86	4,888,832.34	0.414099096
*Rattus tanezumi*	Not Evaluated (NE)	97,17,311.00	4,226,701.05	0.434966118
*Micromys minutus*	Least Concern (LC)	18,265,413.14	3,976,998.23	0.217733823

**Table 5:** Several even-toed ungulates designated by the International Union for Conservation of Nature (IUCN) as most affected by *Brucella* risk.

Latin name	IUCN status	Distribution area (km^2^)	Brucellosis overlap (km^2^)	Prevalence by brucellosis
*Sus scrofa*	Least Concern (LC)	28,012,632.32	7,200,021.43	0.257027663
*Rusa unicolor*	Vulnerable (VU)	7,567,521.34	2,729,116.89	0.360635507
*Muntiacus vaginalis*	Least Concern (LC)	5,871,914.69	2,729,116.89	0.387648283
*Capreolus pygargus*	Least Concern (LC)	3,512,341.66	2,070,177.65	0.58940099
*Muntiacus reevesi*	Least Concern (LC)	2,322,081.59	1,563,987.44	0.673528204
*Tragelaphus scriptus*	Least Concern (LC)	12,063,886.76	1,477,672.11	0.122487234
*Sylvicapra grimmia*	Least Concern (LC)	13,575,937.24	1,423,395.15	0.104846916
*Capricornis sumatraensis*	Vulnerable (VU)	3,512,341.66	1,248,042.83	0.355330702
*Elaphodus cephalophus*	Near Threatened (NT)	1,944,159.32	1,189,053.17	0.611602741
*Kobus ellipsiprymnus*	Least Concern (LC)	8,949,676.32	1,083,023.71	0.121012613

**Figure 5:**
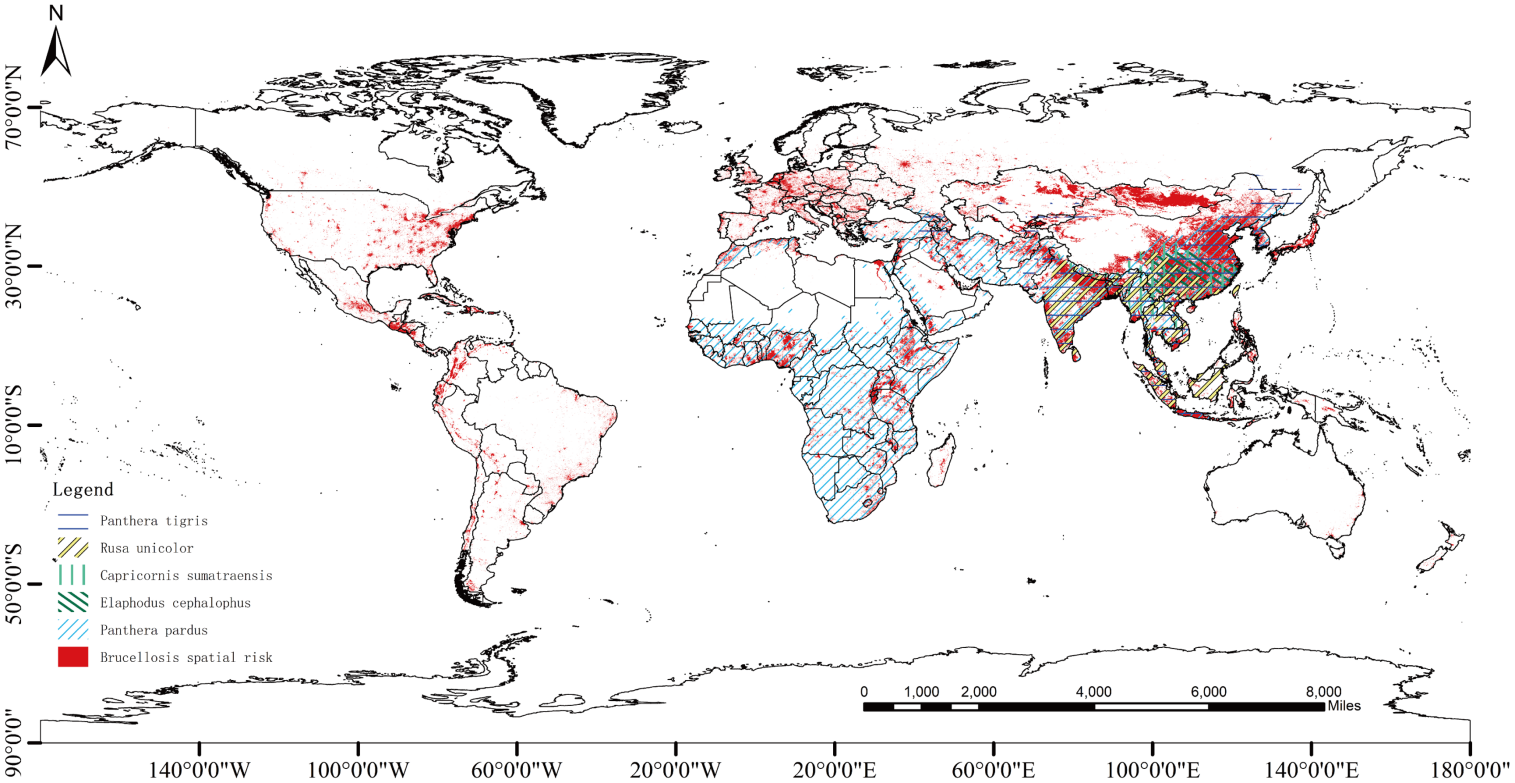
Brucellosis risk area and wildlife distribution overlap map. The map was generated in ArcGIS 10.6 using raster outputs derived from the MaxEnt model.

In order to more clearly show the affected areas of several vulnerable (VU), near threatened (NT), and endangered (EN) animals, we performed a geographical overlay analysis of wildlife species distribution data and brucellosis risk layers affecting livestock. The results showed that geographic areas where the distribution ranges of threatened wildlife species overlap with predicted livestock brucellosis risk zones (Figure 5). These overlapping areas highlight potential interfaces where wildlife may be exposed to environments suitable for *Brucella* circulation.

## 4 Discussion

In this study, we developed a global ecological risk map of brucellosis using an ecological niche modeling framework integrating human, livestock, wildlife, and environmental datasets. The results demonstrated that predicted high-risk areas are primarily concentrated in Asia, while additional hotspots occur in eastern and western Africa. In contrast, only scattered high-risk areas were identified in the Americas and Europe. These findings are generally consistent with previous studies identifying Asia as an persistent endemic area, particularly in the Middle East, for brucellosis (Laine et al., 2023; Negrón et al., 2019).

An important strength and novel contribution of this study lies in its integrated global-scale assessment of brucellosis risk across human, livestock, and wildlife systems within a unified One Health framework. Previous studies have primarily focused on localized epidemiological investigations or single-host risk assessments, whereas our study simultaneously incorporated global human population data, four major livestock host groups (sheep, goats, cattle, and water buffaloes), and the distribution ranges of more than 5,000 wildlife species. This comprehensive integration enabled a broader evaluation of the ecological interfaces associated with potential brucellosis exposure and transmission risk. The risk maps and quantitative estimates from this study can directly support the global brucellosis control roadmap of WOAH and FAO, and provide a data basis for the disease risk assessment of threatened species by IUCN.

The geographic heterogeneity observed in our study likely reflects substantial differences in veterinary infrastructure, livestock management systems, socioeconomic conditions, and disease surveillance capacity. In many high-income countries, long-term vaccination campaigns, test-and-slaughter programs, and coordinated surveillance systems have significantly reduced brucellosis prevalence (Zhang et al., 2018). However, such approaches are often difficult to implement in low-income developing countries because of high economic costs, logistical challenges, and limited public health resources. Consequently, Brucellosis remains endemic in many resource-limited settings where rapid population growth, insufficient veterinary services, and weak surveillance systems may contribute to delayed detection and reporting of cases, thereby hindering timely control measures and allowing continued disease persistence and transmission (Laine et al., 2023). Although the Americas and Europe generally exbibit lower predicted risk level, localized hotspots still occur in some areas due to imported infections, international travel, laboratory-acquired infections, and transboundary trade of livestock and unpasteurized dairy products (Negrón et al., 2019). These findings highlight the importance of strengthening international surveillance coordination and cross-border disease reporting systems to improve global brucellosis prevention and control.

Our analysis further revealed that population density was the primary driver responsible for the spatial distribution of brucellosis risk. This finding is consistent with previous studies demonstrating that densely populated regions often experience increased livestock production, intensified animal trade, and greater human-animal contact opportunities, all of which may facilitate the transmission of zoonotic pathogens (Zhao et al., 2022; Zhang et al., 2018). In addition, population mobility, including migration and international travel, may contribute to the introduction and spread of brucellosis across regions (Liu et al., 2024). Importantly, these associations should not be interpreted as direct causal relationships but rather as ecological correlates identified by the modeling framework.

Our findings revealed a negative association between urban water and brucellosis incidence. This pattern may reflect the indirect effects associated with more urbanized environments, such as improved sanitation infrastructure, safer water management systems, and stronger public health services, which could reduce opportunities for pathogen transmission. A previous studies have suggested that contaminated water sources can contribute to *Brucella* transmission, particularly in areas where wildlife and livestock share common water points (Ganter, 2015). In pastoral and agricultural regions lacking reliable sanitation or water infrastructure, environmental persistence of *Brucella* may further increase exposure opportunities. Collectively, these findings suggest that ecological interfaces shaped by land-use change, livestock intensification, and infrastructure disparities may play important roles in shaping global brucellosis risk patterns.

Our population-based estimates indicated that approximately 58.3 ~ 61.5 million people reside in areas predicted to be at elevated risk for brucellosis, with the majority concentrated in Asia, sub-Saharan Africa, and South America. Many of these populations rely on small-scale livestock farming under resource-limited conditions, where livestock serve not only as essential sources of food and household income but also as critical socioeconomic assets that buffer against poverty and livelihood instability. The persistence and transmission of *Brucella* spp. in these settings are frequently associated with unregulated animal movement, inadequate veterinary infrastructure, limited disease surveillance capacity, and the consumption of unpasteurized dairy products. Together, these factors may perpetuate cycles of infection, economic vulnerability, and limited access to effective disease prevention measures. In addition, the chronic and debilitating nature of brucellosis can result in prolonged disability, reduced labor productivity, and substantial out-of-pocket healthcare expenditures, thereby imposing considerable burdens on affected households, particularly in regions with inadequate healthcare access and weak social protection systems. Furthermore, zoonotic spillover events are often underrecognized because of limited diagnostic capacity, weak surveillance systems, and social stigma associated with disease reporting, which may further complicate accurate burden estimation and disease control efforts. Our findings therefore provide a broader perspective on the global population potentially exposed to brucellosis and complement previous estimates focused primarily on annual incidence. Previous studies estimated that more than 1.6 ~ 2.1 million new human brucellosis cases occur annually worldwide, substantially exceeding the historically cited estimate of 500,000 cases per year (Laine et al., 2023). Collectively, these findings highlight the disproportionate burden of brucellosis on rural and pastoral communities, where livestock play central economic, nutritional, and cultural roles. This underscores the importance of integrating brucellosis prevention and control strategies with broader poverty reduction, rural healthcare improvement, and One Health initiatives.

In addition to human populations, our study estimated that approximately 1.0 ~ 1.2 billion livestock animals, including sheep, goats, cattle, and buffalo, occur within predicted high-risk areas globally. Because livestock represent the primary reservoir hosts for *Brucella* spp., these findings highlight the potential scale of economic and veterinary impacts associated with the disease.

Brucellosis has been previously reported in a wide range of wildlife species, including red deer, wild boar, African buffalo, wildebeest, zebra, lions, baboons, impala, hyenas, horses, camels, and coyotes. Among these species, *Sus scrofa* (wild boar) occupies the largest *Brucella*-affected area, estimated at 7,200,021.43 km^2^, representing 0.25% of its total population range. Wild boars serve as major reservoirs and vectors of *Brucella* transmission, because of their wide geographic distribution, high mobility, and frequent interactions with livestock and human-associated environments (Ruano et al., 2025). Infected wild boars contaminate the environment through feces, run-off products, and secretions, thereby increasing the risk of exposure for other animals and humans (Fredriksson-Ahomaa, 2019; Silvestri and Calfee, 2014). Moreover, contact between wild boars and domestic animals such as cattle, sheep, and pigs increase the risk of *Brucella* infection in domestic animals. Such interactions are particularly important in agricultural and pastoral ecosystems where wildlife, livestock, and humans frequently share landscapes and water resources.

More broadly, the interface between wildlife and domestic animals represents an important challenge for brucellosis control. Cross-species transmission may complicate eradication efforts by maintaining environmental reservoirs and enabling pathogen persistence within multi-host systems (Brennan et al., 2017; Sambu et al., 2021). Consequently, effective brucellosis control requires integrated surveillance strategies that incorporate ecological risk assessment, public health strategies, and wildlife monitoring. Our study identified several threatened wildlife species whose geographic ranges overlap with predicted high-risk regions. Species at greater risk include *Panthera tigris* (Endangered, EN), *Panthera pardus* (Vulnerable, VU), *Rusa unicolor* (Vulnerable, VU), *Capricornis sumatraensis* (Vulnerable, VU), and *Elaphodus cephalophus* (Near Threatened, NT). *Brucella* can severely impact the reproductive success of *Panthera tigris*, hindering population recovery (Hensel et al., 2022). Monitoring and managing wild *Panthera tigris* populations are already challenging, and the presence of *Brucella* further complicates conservation efforts. Increased resources and efforts are required to monitor and control the spread of the disease, elevating the difficulty and cost of tiger conservation (Godfroid, 2017).

## 5 Limitations

First, significant reporting bias and data heterogeneity likely exist across countries and regions. In low-income countries, limited surveillance capacity and underreporting may lead to an underestimation of disease occurrence, while in high-income countries, the high density of surveillance records may bias the model toward environmental characteristics specific to those regions (Syfert et al., 2013). Second, the current modeling framework did not differentiate among the ecological niche characteristics of different *Brucella* species or strains, which may vary in host preference, transmission ecology, and environmental adaptability. Third, although spatial thinning was applied to reduce sampling bias and spatial autocorrelation, the model was validated using subsets of the same occurrence dataset rather than fully independent spatial datasets. Therefore, some degree of performance inflation cannot be excluded, and further validation using spatially independent data would help strengthen the generalizability of the model predictions. In addition, the analysis did not incorporate time dynamics, seasonal fluctuations, vaccination, other intervention measures, and the evolution of pathogen factors, all of which may influence the spatial and temporal patterns of brucellosis transmission. Finally, overlap identified between wildlife distributions and predicted high-risk areas should be interpreted cautiously, as ecological overlap does not necessarily indicate confirmed infection, host susceptibility, or active pathogen transmission.

Future studies integrating longitudinal surveillance data, molecular epidemiology, host-specific transmission dynamics, and spatially independent validation datasets may improve both the predictive reliability and ecological interpretation of global brucellosis risk assessments. Therefore, the present study should be viewed as a large-scale exploratory risk assessment and proof-of-concept framework rather than a definitive prediction of future disease dynamics.

## 6 Conclusion

Brucellosis remains a globally neglected yet important zoonotic disease affecting livestock, wildlife, and human populations, and continues to impose substantial public health and economic burdens worldwide. Despite ongoing control efforts, brucellosis remains endemic in many regions, particularly in Asia and Africa, where ecological complexity and limited public health infrastructure continue to hinder effective disease control. In this study, we applied an ecological niche modeling approach to delineate the global distribution of brucellosis, identify major environmental and anthropogenic drivers, and evaluate potential cross-species exposure patterns at the human-livestock-wildlife interface. Our findings underscore the challenges in controlling interspecies transmission between wildlife and domestic animals. Integrating spatial risk modeling with poverty-targeted public health strategies may improve resource allocation efficiency, facilitate the identification of vulnerable populations, and strengthen early warning and surveillance systems. More broadly, incorporating brucellosis prevention into poverty reduction and One Health framework aligns closely with global health equity priorities and may contribute to achieving the Sustainable Development Goals (SDGs), particularly those related to zoonotic disease control, food security, and the reduction of health disparities.

## Data Availability

The original contributions presented in the study are included in the article/Supplementary material, further inquiries can be directed to the corresponding authors.

## Footnotes

1
www.worldclim.org

2
http://www.cs.princeton.edu/-schapire/MaxEnt/
