# Supplementary material for: Global distribution and multidimensional risk assessment of brucellosis in humans, livestock, and wildlife
Source: Front Microbiol. 2026 Jul 6;17:1844035. doi: 10.3389/fmicb.2026.1844035 (PMC13381503; doi:10.3389/fmicb.2026.1844035)
Supplement: Supplementary file 1 [file Data_Sheet_1.PDF]

Table S1: The mammals designated by the International Union for Conservation of Nature (IUCN) as most affected by *Brucella* risk

No.	sci_name	Brucellosis Overlap（M²）	Brucellosis Overlap（km²）
1	Mus musculus	1.15276E+13	11527645.99
2	Vulpes vulpes	8.45913E+12	8459133.90
3	Panthera pardus	7.3915E+12	7391504.42
4	Sus scrofa	7.20002E+12	7200021.43
5	Rattus rattus	7.02808E+12	7028081.82
6	Canis lupus	6.24257E+12	6242572.43
7	Arctonyx albogularis	5.24241E+12	5242413.06
8	Panthera tigris	4.88883E+12	4888832.34
9	Rattus tanezumi	4.2267E+12	4226701.05
10	Micromys minutus	3.977E+12	3976998.23
11	Prionailurus bengalensis	3.88972E+12	3889718.34
12	Meles leucurus	3.57844E+12	3578443.01
13	Viverricula indica	3.55971E+12	3559706.78
14	Mustela sibirica	3.49398E+12	3493982.35
15	Ursus thibetanus	3.39882E+12	3398815.38
16	Nyctereutes procyonoides	3.21864E+12	3218639.82
17	Suncus murinus	3.20681E+12	3206813.47
18	Mustela nivalis	3.15324E+12	3153240.61
19	Rhinolophus ferrumequinum	3.1156E+12	3115604.96
20	Macaca mulatta	3.07763E+12	3077630.69
21	Mellivora capensis	2.94928E+12	2949277.76
22	Lepus tolai	2.94809E+12	2948086.95
23	Herpestes auropunctatus	2.90527E+12	2905269.58
24	Vespertilio sinensis	2.8921E+12	2892104.47
25	Felis lybica	2.8512E+12	2851204.47
26	Acinonyx jubatus	2.77657E+12	2776567.06
27	Rusa unicolor	2.72912E+12	2729116.90
28	Rousettus leschenaultii	2.65978E+12	2659778.56
29	Apodemus agrarius	2.59443E+12	2594434.12
30	Niviventer confucianus	2.4984E+12	2498398.37
31	Paguma larvata	2.49774E+12	2497741.78
32	Mustela eversmanii	2.47985E+12	2479848.16
33	Martes foina	2.45532E+12	2455324.23
34	Pipistrellus abramus	2.44908E+12	2449075.52
35	Canis aureus	2.44079E+12	2440787.73
36	Paradoxurus hermaphroditus	2.42106E+12	2421064.90
37	Hyaena hyaena	2.40042E+12	2400419.77
38	Mustela erminea	2.34688E+12	2346879.32

39	Martes flavigula	2.33397E+12	2333973.12
40	Muntiacus vaginalis	2.27624E+12	2276237.65
41	Eptesicus pachyomus	2.26691E+12	2266910.32
42	Apodemus peninsulae	2.24261E+12	2242613.00
43	Lyroderma lyra	2.22192E+12	2221922.19
44	Hystrix brachyura	2.20381E+12	2203805.62
45	Rhinolophus affinis	2.19852E+12	2198524.18
46	Neofelis nebulosa	2.18937E+12	2189373.18
47	Crocidura suaveolens	2.18612E+12	2186122.15
48	Viverra zibetha	2.18024E+12	2180240.48
49	Cynopterus sphinx	2.17431E+12	2174307.06
50	Felis chaus	2.15109E+12	2151094.57
51	Crocidura attenuata	2.13166E+12	2131658.14
52	Rattus norvegicus	2.11294E+12	2112941.51
53	Erinaceus amurensis	2.11278E+12	2112780.98
54	Myotis laniger	2.07741E+12	2077408.63
55	Otocolobus manul	2.0728E+12	2072795.49
56	Capreolus pygargus	2.07018E+12	2070177.66
57	Myotis davidii	2.05596E+12	2055955.72
58	Ursus arctos	2.05227E+12	2052273.03
59	Vespertilio murinus	2.04054E+12	2040544.86
60	Bandicota indica	2.03028E+12	2030278.35
61	Scotophilus heathii	1.99614E+12	1996144.58
62	Niviventer fulvescens	1.97716E+12	1977163.51
63	Rhinolophus luctus	1.87906E+12	1879057.15
64	Hipposideros armiger	1.87278E+12	1872782.21
65	Scotophilus kuhlii	1.83793E+12	1837930.38
66	Rattus nitidus	1.7657E+12	1765700.30
67	Callosciurus erythraeus	1.75798E+12	1757984.30
68	Herpestes ichneumon	1.73239E+12	1732394.56
69	Herpestes urva	1.72133E+12	1721334.48
70	Rhinopoma hardwickii	1.70776E+12	1707758.45
71	Crocidura shantungensis	1.68343E+12	1683434.35
72	Bandicota bengalensis	1.67376E+12	1673763.28
73	Hypsugo alaschanicus	1.65656E+12	1656557.13
74	Orycteropus afer	1.65301E+12	1653012.44
75	Eptesicus serotinus	1.64667E+12	1646665.14
76	Mastomys natalensis	1.62614E+12	1626144.21
77	Melogale moschata	1.61765E+12	1617654.28
78	Herpestes sanguineus	1.6164E+12	1616399.21
79	Hystrix indica	1.59217E+12	1592167.01

80	Ichneumia albicauda	1.56674E+12	1566741.55
81	Taphozous melanopogon	1.56635E+12	1566352.36
82	Muntiacus reevesi	1.56399E+12	1563987.45
83	Eutamias sibiricus	1.56094E+12	1560942.84
84	Diceros bicornis	1.55364E+12	1553642.34
85	Civettictis civetta	1.54962E+12	1549618.40
86	Nycteris thebaica	1.54785E+12	1547850.63
87	Vulpes corsac	1.54448E+12	1544480.17
88	Pteropus giganteus	1.53766E+12	1537659.53
89	Tscherskia triton	1.53495E+12	1534947.75
90	Caracal caracal	1.53328E+12	1533281.51
91	Taphozous longimanus	1.51948E+12	1519481.42
92	Crocidura olivieri	1.50986E+12	1509856.35
93	Rhinolophus pearsonii	1.50117E+12	1501168.43
94	Mustela altaica	1.4802E+12	1480195.34
95	Tragelaphus scriptus	1.47767E+12	1477672.12
96	Vormela peregusna	1.4715E+12	1471500.50
97	Crocuta crocuta	1.46606E+12	1466057.43
98	Lepus nigricollis	1.46169E+12	1461691.06
99	Hipposideros pratti	1.44809E+12	1448092.92
100	Pipistrellus pipistrellus	1.44324E+12	1443244.96
101	Sylvicapra grimmia	1.4234E+12	1423395.16
102	Nyctalus plancyi	1.42235E+12	1422345.90
103	Herpestes edwardsii	1.40739E+12	1407393.68
104	Sciurus vulgaris	1.40319E+12	1403186.61
105	Ictonyx striatus	1.39519E+12	1395185.13
106	Pipistrellus coromandra	1.38835E+12	1388350.81
107	Procavia capensis	1.38402E+12	1384022.76
108	Cricetulus barabensis	1.37715E+12	1377154.62
109	Hemiechinus auritus	1.37245E+12	1372451.72
110	Scotomanes ornatus	1.372E+12	1372001.10
111	Tatera indica	1.37153E+12	1371526.47
112	Barbastella leucomelas	1.37117E+12	1371170.36
113	Mesechinus dauuricus	1.36697E+12	1366965.16
114	Genetta genetta	1.36658E+12	1366583.88
115	Genetta maculata	1.36357E+12	1363573.95
116	Nycteris macrotis	1.34352E+12	1343520.54
117	Rhinolophus sinicus	1.33802E+12	1338016.77
118	Neoromicia capensis	1.33061E+12	1330608.42
119	Lepus europaeus	1.32975E+12	1329754.08
120	Mustela kathiah	1.32925E+12	1329246.73

121	Pipistrellus kuhlii	1.31847E+12	1318468.06
122	Rhinolophus lepidus	1.31821E+12	1318212.28
123	Allactaga sibirica	1.31299E+12	1312988.28
124	Hypsugo pulveratus	1.30822E+12	1308218.74
125	Manis pentadactyla	1.30327E+12	1303267.00
126	Mus booduga	1.30171E+12	1301711.14
127	Eidolon helvum	1.29593E+12	1295931.59
128	Scotophilus dinganii	1.28636E+12	1286356.20
129	Rhinolophus landeri	1.28634E+12	1286337.35
130	Myotis chinensis	1.28266E+12	1282658.66
131	Rhinolophus pusillus	1.28154E+12	1281539.79
132	Meles meles	1.26435E+12	1264347.30
133	Vandeleuria oleracea	1.25496E+12	1254962.77
134	Rhizomys sinensis	1.25014E+12	1250135.41
135	Capricornis sumatraensis	1.24804E+12	1248042.83
136	Murina leucogaster	1.24722E+12	1247219.67
137	Myotis blythii	1.24713E+12	1247125.30
138	Myotis fimbriatus	1.22833E+12	1228325.03
139	Petaurista philippensis	1.21306E+12	1213056.07
140	Barbastella darjelingensis	1.20873E+12	1208726.37
141	Nycteris hispida	1.199E+12	1199002.29
142	Nothocricetulus migratorius	1.19216E+12	1192157.57
143	Elaphodus cephalophus	1.18905E+12	1189053.17
144	Manis crassicaudata	1.18029E+12	1180293.81
145	Neoromicia nana	1.17332E+12	1173318.30
146	Lynx lynx	1.15971E+12	1159709.93
147	Chaerephon pumilus	1.15693E+12	1156931.11
148	Mus terricolor	1.15652E+12	1156516.59
149	Myospalax psilurus	1.14332E+12	1143319.93
150	Canis adustus	1.13738E+12	1137378.19
151	Rhinolophus hipposideros	1.13439E+12	1134387.53
152	Vulpes bengalensis	1.12925E+12	1129253.71
153	Tylonycteris pachypus	1.12751E+12	1127507.34
154	Myotis altarium	1.12554E+12	1125540.90
155	Nyctalus noctula	1.11395E+12	1113953.48
156	Myotis muricola	1.10934E+12	1109335.72
157	Sorex minutus	1.08373E+12	1083731.53
158	Rhinolophus rex	1.08316E+12	1083162.32
159	Kobus ellipsiprymnus	1.08302E+12	1083023.71
160	Golunda ellioti	1.07828E+12	1078282.99
161	Lepus victoriae	1.06195E+12	1061946.64

162	Eothenomys melanogaster	1.05914E+12	1059138.30
163	Rattus losea	1.05792E+12	1057921.60
164	Pipistrellus javanicus	1.05729E+12	1057292.67
165	Phacochoerus africanus	1.05218E+12	1052179.73
166	Meriones meridianus	1.05194E+12	1051939.09
167	Leopoldamys edwardsi	1.0471E+12	1047096.01
168	Berylmys bowersi	1.04527E+12	1045269.07
169	Tadarida brasiliensis	1.04282E+12	1042822.32
170	Mustela frenata	1.04136E+12	1041355.71
171	Cricetulus longicaudatus	1.02526E+12	1025262.91
172	Crocidura sibirica	1.01647E+12	1016467.14
173	Rhinolophus fumigatus	1.00994E+12	1009943.48
174	Petaurista petaurista	1.00943E+12	1009434.67
175	Odocoileus virginianus	1.00832E+12	1008316.99
176	Megaderma spasma	1.00478E+12	1004783.35
177	Gazella subgutturosa	1.00217E+12	1002165.79
178	Apodemus draco	9.8765E+11	987650.18
179	Martes martes	9.82876E+11	982875.53
180	Meriones unguiculatus	9.82534E+11	982534.24
181	Lissonycteris angolensis	9.80833E+11	980833.19
182	Rhinolophus macrotis	9.76026E+11	976026.39
183	Mungos mungo	9.73221E+11	973220.62
184	Lasiurus cinereus	9.72513E+11	972513.33
185	Eptesicus fuscus	9.72482E+11	972482.46
186	Galago senegalensis	9.70201E+11	970201.44
187	Axis axis	9.68069E+11	968069.04
188	Neoromicia rendalli	9.67775E+11	967774.62
189	Nyctalus leisleri	9.67591E+11	967590.89
190	Cuon alpinus	9.6572E+11	965719.63
191	Mops condylurus	9.62909E+11	962908.73
192	Taphozous mauritianus	9.60074E+11	960073.63
193	Pipistrellus ceylonicus	9.59042E+11	959041.82
194	Canis lupaster	9.5867E+11	958669.63
195	Funambulus pennantii	9.57984E+11	957983.86
196	Dryomys nitedula	9.54365E+11	954364.69
197	Hipposideros ruber	9.45398E+11	945397.62
198	Myotis nipalensis	9.38827E+11	938827.18
199	Hystrix cristata	9.38317E+11	938316.55
200	Hypsugo savii	9.35072E+11	935071.57
201	Kerivoula lanosa	9.254E+11	925400.31
202	Dremomys pernyi	9.21628E+11	921628.38

203	Scotozous dormeri	9.17554E+11	917554.18
204	Capreolus capreolus	9.14243E+11	914242.50
205	Tamiops maritimus	9.13112E+11	913111.99
206	Hipposideros fulvus	9.11861E+11	911861.46
207	Ochotona dauurica	9.11812E+11	911812.23
208	Urocyon cinereoargenteus	9.10547E+11	910547.41
209	Glauconycteris variegata	9.08688E+11	908687.96
210	Papio anubis	9.03169E+11	903168.92
211	Epomophorus minor	8.98881E+11	898881.15
212	Sciurotamias davidianus	8.94478E+11	894477.57
213	Puma concolor	8.92656E+11	892656.46
214	Myotis emarginatus	8.92646E+11	892646.39
215	Eonycteris spelaea	8.90448E+11	890448.23
216	Myotis horsfieldii	8.90001E+11	890001.06
217	Prionodon pardicolor	8.89892E+11	889891.92
218	Apodemus sylvaticus	8.88917E+11	888917.36
219	Canis latrans	8.82973E+11	882973.12
220	Moschus berezovskii	8.81048E+11	881047.60
221	Ellobius tancrei	8.79062E+11	879061.94
222	Pipistrellus nathusii	8.77676E+11	877675.58
223	Crocidura fuliginosa	8.77405E+11	877404.90
224	Lepus capensis	8.77152E+11	877152.48
225	Crocidura fuscomurina	8.76294E+11	876294.35
226	Dasypus novemcinctus	8.73935E+11	873934.58
227	Ourebia ourebi	8.7278E+11	872780.47
228	Prionailurus rubiginosus	8.71933E+11	871932.57
229	Procyon lotor	8.71031E+11	871030.69
230	Macaca thibetana	8.70006E+11	870005.91
231	Moschus moschiferus	8.64295E+11	864295.31
232	Petaurista elegans	8.62326E+11	862326.37
233	Kerivoula picta	8.61905E+11	861905.33
234	Rattus exulans	8.61688E+11	861687.78
235	Hipposideros larvatus	8.59318E+11	859318.13
236	Potamochoerus larvatus	8.57578E+11	857578.14
237	Myotis frater	8.54811E+11	854810.56
238	Millardia meltada	8.54368E+11	854367.82
239	Lavia frons	8.4589E+11	845889.60
240	Atherurus macrourus	8.39788E+11	839788.44
241	Rhizomys pruinosus	8.32767E+11	832766.55
242	Desmodus rotundus	8.30142E+11	830142.36
243	Myotis pilosus	8.29804E+11	829803.72

244	Alexandromys fortis	8.29093E+11	829092.79
245	Boselaphus tragocamelus	8.23295E+11	823294.60
246	Phodopus campbelli	8.20228E+11	820227.72
247	Coelops frithii	8.19806E+11	819805.70
248	Myotis daubentonii	8.16953E+11	816952.86
249	Atelerix albiventris	8.16359E+11	816359.13
250	Apodemus flavicollis	8.14681E+11	814680.93
251	Sorex araneus	8.08934E+11	808934.18
252	Helogale parvula	8.06414E+11	806414.07
253	Rousettus amplexicaudatus	8.03474E+11	803474.05
254	Heterohyrax brucei	8.02014E+11	802014.28
255	Microtus arvalis	8.00583E+11	800582.56
256	Hipposideros galeritus	7.99242E+11	799241.64
257	Suncus etruscus	7.93822E+11	793822.00
258	Hipposideros gentilis	7.93535E+11	793534.60
259	Tupaia belangeri	7.91665E+11	791665.44
260	Sylvilagus floridanus	7.88237E+11	788237.37
261	Apodemus uralensis	7.86781E+11	786781.26
262	Mustela putorius	7.84166E+11	784166.12
263	Rousettus aegyptiacus	7.81009E+11	781009.07
264	Mastomys erythroleucus	7.8087E+11	780869.72
265	Dasymys incomtus	7.78499E+11	778499.33
266	Eptesicus gobiensis	7.77503E+11	777502.70
267	Myotis sibiricus	7.75699E+11	775699.21
268	Scaptochirus moschatus	7.75429E+11	775428.78
269	Talpa europaea	7.67243E+11	767242.67
270	Mus musculoides	7.61804E+11	761804.15
271	Suncus megalura	7.6045E+11	760449.55
272	Didelphis virginiana	7.59289E+11	759288.67
273	Xerus erythropus	7.48877E+11	748877.06
274	Chaerephon ansorgei	7.487E+11	748700.42
275	Oreotragus oreotragus	7.46615E+11	746614.91
276	Lemniscomys striatus	7.45564E+11	745564.49
277	Tetracerus quadricornis	7.45011E+11	745010.56
278	Lasiurus blossevillii	7.44689E+11	744688.96
279	Clethrionomys glareolus	7.43683E+11	743683.11
280	Arvicanthis niloticus	7.43159E+11	743158.92
281	Molossus molossus	7.39359E+11	739358.80
282	Kerivoula hardwickii	7.39128E+11	739128.06
283	Plecotus auritus	7.35586E+11	735586.00
284	Myotis mystacinus	7.35171E+11	735171.25

285	Myotis myotis	7.29442E+11	729441.82
286	Herpestes javanicus	7.2838E+11	728380.40
287	Sorex tundrensis	7.26181E+11	726180.79
288	Redunca redunca	7.25679E+11	725678.95
289	Saccolaimus saccolaimus	7.19999E+11	719999.33
290	Dama dama	7.17918E+11	717918.00
291	Hipposideros diadema	7.1785E+11	717849.67
292	Lasiopodomys gregalis	7.16702E+11	716702.28
293	Meriones libycus	7.15677E+11	715676.93
294	Cynopterus brachyotis	7.14847E+11	714846.64
295	Glossophaga soricina	7.14419E+11	714418.89
296	Helarctos malayanus	7.08804E+11	708804.36
297	Barbastella barbastellus	7.03756E+11	703756.11
298	Hipposideros ater	7.03438E+11	703438.33
299	Syncerus caffer	7.01727E+11	701726.85
300	Crocidura leucodon	6.93597E+11	693596.83
301	Nandinia binotata	6.89946E+11	689946.34
302	Eptesicus furinalis	6.89072E+11	689071.78
303	Canis mesomelas	6.86621E+11	686620.75
304	Panthera uncia	6.85737E+11	685736.54
305	Macaca fascicularis	6.81064E+11	681064.13
306	Ceratotherium simum	6.79882E+11	679881.53
307	Macroglossus sobrinus	6.76121E+11	676120.76
308	Myotis tricolor	6.71867E+11	671866.61
309	Phodopus roborovskii	6.68446E+11	668446.05
310	Artibeus lituratus	6.6691E+11	666909.69
311	Asellia tridens	6.643E+11	664299.56
312	Rhinopoma microphyllum	6.6052E+11	660519.56
313	Plecotus ognevi	6.55944E+11	655944.12
314	Cervus elaphus	6.55839E+11	655839.34
315	Tylonycteris robustula	6.55688E+11	655688.04
316	Maxomys surifer	6.54711E+11	654711.10
317	Epomophorus wahlbergi	6.54477E+11	654477.48
318	Glis glis	6.54041E+11	654040.71
319	Rattus argentiventer	6.52529E+11	652528.50
320	Microtus agrestis	6.50425E+11	650425.26
321	Lepus timidus	6.48216E+11	648216.50
322	Chaerephon plicatus	6.47475E+11	647475.26
323	Rhinolophus blasii	6.47049E+11	647049.27
324	Muscardinus avellanarius	6.46737E+11	646736.89
325	Myotis welwitschii	6.43309E+11	643309.31

326	Spermophilus dauricus	6.41797E+11	641796.75
327	Typhlomys cinereus	6.38282E+11	638281.70
328	Poecilogale albinucha	6.3452E+11	634519.83
329	Noctilio leporinus	6.34189E+11	634188.66
330	Hystrix africaeaustralis	6.307E+11	630699.66
331	Cervus canadensis	6.30502E+11	630502.21
332	Myotis nigricans	6.29194E+11	629194.26
333	Tadarida teniotis	6.27878E+11	627878.46
334	Sorex minutissimus	6.27181E+11	627180.61
335	Trogopterus xanthipes	6.24174E+11	624173.99
336	Pecari tajacu	6.23986E+11	623985.72
337	Eospalax fontanierii	6.21166E+11	621166.32
338	Ia io	6.20121E+11	620121.16
339	Molossus rufus	6.19206E+11	619205.78
340	Macroglossus minimus	6.18432E+11	618432.28
341	Ursus americanus	6.17875E+11	617875.43
342	Mephitis mephitis	6.17477E+11	617476.55
343	Scotoecus hirundo	6.16233E+11	616233.42
344	Scotophilus viridis	6.12871E+11	612870.90
345	Anomalurus derbianus	6.11739E+11	611738.68
346	Nyctinomops macrotis	6.117E+11	611699.65
347	Lepus sinensis	6.07591E+11	607591.26
348	Myotis pequinius	6.06893E+11	606893.46
349	Anoura geoffroyi	6.06841E+11	606841.19
350	Tadarida aegyptiaca	6.03116E+11	603115.86
351	Crocidura lasiura	6.02463E+11	602462.98
352	Leopardus pardalis	6.02189E+11	602189.43
353	Crocidura viaria	6.02101E+11	602101.22
354	Eumops auripendulus	6.00302E+11	600302.11
355	Chlorocebus pygerythrus	5.97387E+11	597387.40
356	Mus caroli	5.95273E+11	595272.98
357	Herpestes smithii	5.95029E+11	595029.07
358	Mus minutoides	5.94364E+11	594363.73
359	Macaca arctoides	5.92807E+11	592806.85
360	Phataginus tricuspis	5.92115E+11	592115.44
361	Micropteropus pusillus	5.90016E+11	590016.38
362	Eptesicus nilssonii	5.89116E+11	589115.62
363	Neoromicia somalica	5.88279E+11	588279.08
364	Ratufa bicolor	5.87366E+11	587366.01
365	Otomops martiensseni	5.8732E+11	587320.36
366	Carollia perspicillata	5.87185E+11	587184.99

367	Rhinolophus euryale	5.85968E+11	585967.80
368	Eira barbara	5.83368E+11	583368.15
369	Marmota himalayana	5.80512E+11	580511.70
370	Pteropus vampyrus	5.79974E+11	579974.46
371	Nesokia indica	5.77888E+11	577888.09
372	Smutsia temminckii	5.74442E+11	574441.75
373	Arctictis binturong	5.72555E+11	572554.97
374	Ovis ammon	5.7035E+11	570350.10
375	Myotis nattereri	5.70096E+11	570096.20
376	Uranomys ruddi	5.68154E+11	568153.80
377	Pseudois nayaur	5.66866E+11	566865.57
378	Rattus andamanensis	5.66824E+11	566824.18
379	Glaucomys volans	5.62135E+11	562135.23
380	Tadarida ventralis	5.58917E+11	558917.15
381	Lynx rufus	5.58508E+11	558507.76
382	Tragelaphus strepsiceros	5.57554E+11	557553.85
383	Myotis bechsteinii	5.57383E+11	557382.65
384	Nycticeinops schlieffeni	5.56918E+11	556918.14
385	Lagurus lagurus	5.56444E+11	556443.85
386	Petaurista alborufus	5.53664E+11	553664.19
387	Cuniculus paca	5.53222E+11	553221.72
388	Nyctinomops laticaudatus	5.51594E+11	551593.63
389	Peromyscus maniculatus	5.5054E+11	550540.28
390	Saccopteryx bilineata	5.50275E+11	550275.38
391	Perimyotis subflavus	5.47422E+11	547421.63
392	Lasionycteris noctivagans	5.44519E+11	544519.33
393	Erinaceus roumanicus	5.44203E+11	544202.98
394	Myotis albescens	5.43956E+11	543955.90
395	Gerbilliscus kempi	5.43204E+11	543204.36
396	Erinaceus europaeus	5.42348E+11	542347.93
397	Marmota sibirica	5.40028E+11	540028.46
398	Potos flavus	5.38406E+11	538406.37
399	Rhinolophus hildebrandtii	5.38326E+11	538325.52
400	Grammomys dolichurus	5.36539E+11	536538.95
401	Myotis brandtii	5.35684E+11	535684.48
402	Philantomba monticola	5.35307E+11	535307.44
403	Mus pahari	5.33276E+11	533276.29
404	Allocricetulus curtatus	5.32733E+11	532732.75
405	Alcelaphus buselaphus	5.3061E+11	530609.80
406	Rhinolophus clivosus	5.30008E+11	530008.29
407	Mimetillus moloneyi	5.29777E+11	529776.96

408	Leopoldamys sabanus	5.29539E+11	529539.08
409	Gerbilliscus robustus	5.29523E+11	529522.61
410	Rhinopoma macinnesi	5.29104E+11	529104.49
411	Myotis ikonnikovi	5.28833E+11	528832.92
412	Tragelaphus oryx	5.28244E+11	528243.82
413	Phyllostomus discolor	5.26901E+11	526901.03
414	Pteromys volans	5.26832E+11	526831.72
415	Lasiurus ega	5.25285E+11	525284.89
416	Diphylla ecaudata	5.23628E+11	523628.36
417	Lepus oiostolus	5.21695E+11	521694.57
418	Hipposideros cineraceus	5.20328E+11	520328.43
419	Peromyscus leucopus	5.15979E+11	515979.45
420	Lasiopodomys mandarinus	5.14542E+11	514542.07
421	Neoromicia tenuipinnis	5.12919E+11	512918.53
422	Macrophyllum macrophyllum	5.11872E+11	511872.28
423	Antilope cervicapra	5.0913E+11	509129.65
424	Cardiocranius paradoxus	5.08122E+11	508121.51
425	Cryptotis parva	5.07778E+11	507778.04
426	Otolemur crassicaudatus	5.07357E+11	507357.48
427	Lasiurus borealis	5.05016E+11	505016.06
428	Myotis lucifugus	5.01585E+11	501585.23
429	Cricetomys gambianus	5.01566E+11	501565.65
430	Rattus pyctoris	5.01557E+11	501556.93
431	Didelphis marsupialis	4.99974E+11	499973.70
432	Rhinolophus osgoodi	4.99875E+11	499875.43
433	Rhombomys opimus	4.99212E+11	499211.67
434	Sciurus carolinensis	4.98488E+11	498488.22
435	Microtus subterraneus	4.96763E+11	496763.39
436	Manis javanica	4.95992E+11	495991.60
437	Heliosciurus gambianus	4.94313E+11	494312.68
438	Dendromus melanotis	4.93649E+11	493649.27
439	Lemniscomys zebra	4.92972E+11	492972.36
440	Crocidura turba	4.91075E+11	491075.15
441	Miniopterus africanus	4.91032E+11	491032.49
442	Dendromus mystacalis	4.90677E+11	490677.44
443	Cricetomys ansorgei	4.90495E+11	490495.14
444	Sorex caecutiens	4.89296E+11	489296.34
445	Scotoecus albofuscus	4.8722E+11	487220.00
446	Anourosorex squamipes	4.8696E+11	486960.20
447	Diclidurus albus	4.85693E+11	485692.96
448	Moschus chrysogaster	4.85499E+11	485499.38

449	Trachops cirrhosus	4.84246E+11	484246.18
450	Miniopterus magnater	4.8365E+11	483649.84
451	Myotis riparius	4.82335E+11	482335.27
452	Ochotona macrotis	4.82293E+11	482293.25
453	Mogera insularis	4.80727E+11	480727.30
454	Eptesicus brasiliensis	4.79986E+11	479985.52
455	Mus saxicola	4.7913E+11	479129.63
456	Herpailurus yagouaroundi	4.76203E+11	476203.07
457	Crocidura parvipes	4.75797E+11	475796.64
458	Macaca assamensis	4.75632E+11	475632.34
459	Miniopterus natalensis	4.75505E+11	475504.60
460	Thallomys paedulcus	4.7524E+11	475240.01
461	Eptesicus pachyotis	4.75208E+11	475208.48
462	Erythrocebus patas	4.74304E+11	474303.92
463	Taterillus emini	4.73549E+11	473548.81
464	Gerbillus nanus	4.73275E+11	473275.37
465	Anathana ellioti	4.71988E+11	471988.06
466	Alticola semicanus	4.71348E+11	471348.00
467	Harpiocephalus harpia	4.70985E+11	470985.44
468	Heliosciurus rufobrachium	4.68019E+11	468019.00
469	Rhinolophus malayanus	4.65438E+11	465438.11
470	Plecotus austriacus	4.64504E+11	464504.22
471	Procapra picticaudata	4.62633E+11	462633.33
472	Proteles cristata	4.59869E+11	459869.07
473	Nycteris arge	4.59141E+11	459141.09
474	Myotis keaysi	4.57788E+11	457788.43
475	Cricetus cricetus	4.56939E+11	456939.38
476	Phyllostomus hastatus	4.56203E+11	456202.90
477	Peropteryx macrotis	4.55937E+11	455936.81
478	Crocidura gmelini	4.55858E+11	455858.16
479	Leopardus wiedii	4.55046E+11	455046.24
480	Pipistrellus aladdin	4.52402E+11	452401.86
481	Platyrrhinus helleri	4.52301E+11	452300.59
482	Cephalophus silvicultor	4.50682E+11	450681.90
483	Lonchorhina aurita	4.47898E+11	447898.20
484	Myotis petax	4.47692E+11	447691.71
485	Noctilio albiventris	4.4577E+11	445770.13
486	Thryonomys gregorianus	4.44568E+11	444567.65
487	Chlorocebus tantalus	4.43596E+11	443595.90
488	Mormoops megalophylla	4.43437E+11	443436.85
489	Chaerephon chapini	4.43404E+11	443403.69

490	Oenomys hypoxanthus	4.43063E+11	443062.76
491	Myotis indochinensis	4.41945E+11	441944.95
492	Epomophorus gambianus	4.41202E+11	441201.57
493	Zelotomys hildegardeae	4.38596E+11	438595.75
494	Saccopteryx leptura	4.37183E+11	437182.79
495	Lasiopodomys brandtii	4.36639E+11	436639.37
496	Pipistrellus hesperidus	4.36389E+11	436389.19
497	Hipposideros speoris	4.36326E+11	436326.39
498	Crocidura russula	4.32741E+11	432741.40
499	Chrotopterus auritus	4.31792E+11	431792.23
500	Nycteris grandis	4.3093E+11	430929.53
501	Emballonura monticola	4.30135E+11	430135.01
502	Hipposideros lankadiva	4.29787E+11	429787.40
503	Atherurus africanus	4.29318E+11	429317.59
504	Dendrohyrax arboreus	4.28993E+11	428993.04
505	Lophuromys sikapusi	4.27866E+11	427866.32
506	Alces alces	4.27019E+11	427018.53
507	Scaptonyx fusicaudus	4.25903E+11	425903.16
508	Crocidura nigrofusca	4.24835E+11	424834.60
509	Vulpes ferrilata	4.24719E+11	424719.41
510	Spermophilus erythrogenys	4.24533E+11	424532.76
511	Rhinopoma cystops	4.24353E+11	424353.46
512	Scalopus aquaticus	4.24149E+11	424149.33
513	Gulo gulo	4.23943E+11	423942.97
514	Zapus hudsonius	4.23688E+11	423687.57
515	Felis bieti	4.21893E+11	421892.81
516	Miniopterus schreibersii	4.21846E+11	421846.11
517	Pipistrellus rueppellii	4.20822E+11	420822.38
518	Rhinolophus rouxii	4.2075E+11	420750.12
519	Chaerephon nigeriae	4.20086E+11	420086.29
520	Rhinolophus huananus	4.17072E+11	417072.44
521	Chiropodomys gliroides	4.16423E+11	416422.56
522	Diaemus youngi	4.15983E+11	415982.96
523	Allactaga major	4.15426E+11	415425.65
524	Phylloderma stenops	4.1473E+11	414729.78
525	Nycticebus bengalensis	4.14418E+11	414418.45
526	Sciurus niger	4.12268E+11	412268.17
527	Galagoides thomasi	4.11542E+11	411541.86
528	Glauconycteris argentata	4.11118E+11	411118.17
529	Nyctalus aviator	4.10764E+11	410763.87
530	Promops centralis	4.10684E+11	410684.01

531	Micronycteris megalotis	4.10665E+11	410664.54
532	Microtus pinetorum	4.10458E+11	410457.71
533	Hylomys suillus	4.09741E+11	409741.29
534	Ochotona curzoniae	4.0971E+11	409709.61
535	Genetta thierryi	4.09245E+11	409245.21
536	Tamias striatus	4.07701E+11	407700.94
537	Scotophilus leucogaster	4.07593E+11	407593.14
538	Hylopetes alboniger	4.04638E+11	404638.08
539	Alexandromys oeconomus	4.03872E+11	403872.14
540	Enchisthenes hartii	4.03154E+11	403154.05
541	Uropsilus gracilis	4.02692E+11	402692.38
542	Praomys daltoni	4.02412E+11	402412.16
543	Chiroderma salvini	4.01857E+11	401857.20
544	Microtus pennsylvanicus	4.01163E+11	401163.48
545	Rhynchonycteris naso	4.0007E+11	400070.14
546	Marmota monax	3.9991E+11	399910.03
547	Miniopterus pallidus	3.99812E+11	399811.61
548	Melogale personata	3.97155E+11	397155.33
549	Muntiacus muntjak	3.96946E+11	396946.46
550	Arctogalidia trivirgata	3.96179E+11	396178.94
551	Arctonyx collaris	3.96016E+11	396015.74
552	Elephantulus brachyrhynchus	3.94643E+11	394643.17
553	Myotis septentrionalis	3.94413E+11	394413.16
554	Otocyon megalotis	3.91712E+11	391712.13
555	Callosciurus notatus	3.91507E+11	391506.57
556	Rattus tiomanicus	3.90288E+11	390288.13
557	Cynopterus horsfieldii	3.89692E+11	389691.76
558	Funambulus palmarum	3.87403E+11	387402.64
559	Neoromicia guineensis	3.8732E+11	387320.48
560	Graphiurus kelleni	3.8487E+11	384869.71
561	Crocidura vorax	3.84194E+11	384194.29
562	Ochotona cansus	3.82431E+11	382430.67
563	Lepus tibetanus	3.81196E+11	381195.53
564	Peropteryx kappleri	3.79578E+11	379578.25
565	Myotis formosus	3.79443E+11	379443.31
566	Graphiurus microtis	3.78607E+11	378607.28
567	Tragulus kanchil	3.78171E+11	378170.57
568	Pygeretmus pumilio	3.77993E+11	377992.61
569	Cricetomys emini	3.77721E+11	377721.14
570	Pteronotus personatus	3.76367E+11	376367.44
571	Rhinolophus thomasi	3.76219E+11	376219.27

572	Galagoides demidoff	3.75591E+11	375590.60
573	Cerdocyon thous	3.74587E+11	374586.56
574	Micronycteris minuta	3.74264E+11	374264.16
575	Eliomys quercinus	3.74072E+11	374072.24
576	Nyctinomops aurispinosus	3.73527E+11	373527.27
577	Coleura afra	3.73389E+11	373389.28
578	Apodemus chevrieri	3.73028E+11	373027.58
579	Dremomys rufigenis	3.73012E+11	373011.75
580	Alexandromys limnophilus	3.72885E+11	372884.75
581	Murina suilla	3.72817E+11	372816.62
582	Potamochoerus porcus	3.72314E+11	372314.45
583	Melursus ursinus	3.72112E+11	372111.67
584	Pteronotus davyi	3.71893E+11	371893.47
585	Crocidura hildegardeae	3.70612E+11	370612.40
586	Rhinolophus acuminatus	3.69635E+11	369634.51
587	Lariscus insignis	3.67349E+11	367348.51
588	Gardnerycteris crenulatum	3.67195E+11	367195.40
589	Aepyceros melampus	3.66536E+11	366536.45
590	Nycticeius humeralis	3.66061E+11	366061.45
591	Macronycteris vittatus	3.64945E+11	364945.37
592	Gerbilliscus validus	3.63146E+11	363145.88
593	Redunca arundinum	3.62717E+11	362716.86
594	Rhinolophus borneensis	3.61731E+11	361731.44
595	Carollia brevicauda	3.61495E+11	361495.29
596	Myotis hasseltii	3.60642E+11	360642.07
597	Madromys blanfordi	3.59983E+11	359982.87
598	Bradypus variegatus	3.59087E+11	359087.40
599	Rhinolophus mehelyi	3.58551E+11	358551.27
600	Cynopterus minutus	3.58376E+11	358376.17
601	Petaurista xanthotis	3.58371E+11	358371.02
602	Rhinolophus stheno	3.58332E+11	358332.38
603	Allactaga bullata	3.57591E+11	357590.70
604	Tamiops swinhoei	3.5704E+11	357039.60
605	Gerbilliscus leucogaster	3.57039E+11	357038.75
606	Vulpes cana	3.56316E+11	356315.85
607	Protoxerus stangeri	3.55266E+11	355266.27
608	Hipposideros abae	3.53398E+11	353398.06
609	Tayassu pecari	3.52962E+11	352961.82
610	Dermanura phaeotis	3.51487E+11	351486.98
611	Crocidura monticola	3.51122E+11	351122.42
612	Myotis bocagii	3.49837E+11	349837.35

613	Taxidea taxus	3.48484E+11	348483.56
614	Blarina brevicauda	3.47562E+11	347562.34
615	Epomops franqueti	3.47292E+11	347291.67
616	Taphozous theobaldi	3.46464E+11	346464.30
617	Kerivoula argentata	3.4579E+11	345789.82
618	Epomophorus labiatus	3.42276E+11	342275.89
619	Crocidura luna	3.42222E+11	342221.54
620	Gazella bennettii	3.42147E+11	342147.04
621	Neoromicia zuluensis	3.40381E+11	340380.63
622	Viverra tangalunga	3.40101E+11	340100.57
623	Vampyrum spectrum	3.39639E+11	339639.47
624	Scarturus elater	3.39514E+11	339513.76
625	Pipistrellus nanulus	3.38479E+11	338479.23
626	Metachirus nudicaudatus	3.38143E+11	338142.89
627	Kerivoula pellucida	3.37781E+11	337780.67
628	Microtus socialis	3.3626E+11	336259.67
629	Tamiasciurus hudsonicus	3.34131E+11	334130.51
630	Furipterus horrens	3.31518E+11	331518.25
631	Laephotis wintoni	3.29876E+11	329876.30
632	Chiroderma villosum	3.29203E+11	329203.41
633	Miniopterus australis	3.27908E+11	327907.74
634	Cercopithecus mitis	3.27224E+11	327224.46
635	Myrmecophaga tridactyla	3.27069E+11	327068.85
636	Crocidura hirta	3.26598E+11	326598.16
637	Pipistrellus pygmaeus	3.26498E+11	326498.30
638	Vampyressa thyone	3.26475E+11	326475.10
639	Cheiromeles torquatus	3.25435E+11	325435.46
640	Aselliscus stoliczkanus	3.24841E+11	324841.44
641	Hippotragus equinus	3.24813E+11	324812.88
642	Urocitellus undulatus	3.2472E+11	324719.54
643	Micronycteris schmidtorum	3.24551E+11	324550.67
644	Myotis dasycneme	3.23894E+11	323893.99
645	Uroderma magnirostrum	3.23518E+11	323517.74
646	Colobus guereza	3.22233E+11	322232.54
647	Felis silvestris	3.21972E+11	321972.17
648	Speothos venaticus	3.21531E+11	321530.79
649	Cardioderma cor	3.2101E+11	321009.58
650	Tachyoryctes splendens	3.20702E+11	320701.73
651	Cephalophus rufilatus	3.2006E+11	320060.39
652	Nasua narica	3.20004E+11	320003.89
653	Rhinolophus simulator	3.19673E+11	319673.41

654	Callosciurus nigrovittatus	3.18834E+11	318834.22
655	Saccostomus campestris	3.17421E+11	317420.85
656	Taterillus gracilis	3.15484E+11	315483.64
657	Chaerephon bivittatus	3.14929E+11	314929.33
658	Molossus sinaloae	3.14703E+11	314702.60
659	Alticola argentatus	3.11674E+11	311674.04
660	Nanonycteris veldkampii	3.11424E+11	311424.22
661	Hipposideros cervinus	3.10714E+11	310713.95
662	Nyctalus lasiopterus	3.09443E+11	309442.90
663	Cyclopes didactylus	3.09233E+11	309232.55
664	Praomys jacksoni	3.07059E+11	307059.06
665	Mazama americana	3.0682E+11	306819.59
666	Capra sibirica	3.05076E+11	305076.21
667	Rhinolophus microglobosus	3.03398E+11	303398.26
668	Meriones persicus	3.03324E+11	303324.47
669	Synaptomys cooperi	3.02937E+11	302937.42
670	Niviventer cremoriventer	3.01813E+11	301813.00
671	Sorex cinereus	3.0175E+11	301750.16
672	Coendou prehensilis	3.00507E+11	300507.14
673	Dipus sagitta	3.00288E+11	300287.81
674	Dasyprocta punctata	2.99968E+11	299968.16
675	Dasymys rufulus	2.98556E+11	298556.01
676	Mungos gambianus	2.9853E+11	298529.60
677	Artibeus jamaicensis	2.9783E+11	297829.57
678	Clethrionomys rutilus	2.97413E+11	297413.43
679	Aethomys chrysophilus	2.96181E+11	296181.35
680	Sorex isodon	2.96087E+11	296087.02
681	Murina feae	2.95963E+11	295962.88
682	Centurio senex	2.9544E+11	295439.78
683	Mus triton	2.94979E+11	294978.94
684	Tamiops mcclellandii	2.93843E+11	293842.62
685	Galeopterus variegatus	2.93543E+11	293543.30
686	Crocidura jacksoni	2.93209E+11	293209.32
687	Neodon irene	2.92267E+11	292266.76
688	Jaculus jaculus	2.9175E+11	291749.56
689	Grammomys kuru	2.91299E+11	291299.07
690	Galictis cuja	2.90464E+11	290463.74
691	Meriones tamariscinus	2.88967E+11	288967.11
692	Ochotona thibetana	2.88706E+11	288705.93
693	Semnopithecus entellus	2.88593E+11	288592.80
694	Macronycteris gigas	2.88585E+11	288585.38

695	Meriones crassus	2.88156E+11	288155.53
696	Oligoryzomys fulvescens	2.8698E+11	286979.62
697	Chodsigoa parca	2.86976E+11	286975.80
698	Glossophaga commissarisi	2.86689E+11	286689.16
699	Sorex daphaenodon	2.86369E+11	286368.58
700	Hipposideros cyclops	2.86091E+11	286091.22
701	Myotis siligorensis	2.85518E+11	285518.25
702	Platyrrhinus lineatus	2.85133E+11	285132.98
703	Kobus kob	2.85107E+11	285107.40
704	Cynopterus titthaecheilus	2.8505E+11	285050.26
705	Prionodon linsang	2.85015E+11	285014.60
706	Triaenops afer	2.83601E+11	283601.16
707	Ochotona pallasii	2.83096E+11	283095.57
708	Macaca radiata	2.80989E+11	280989.05
709	Mustela strigidorsa	2.80922E+11	280922.39
710	Myotis oxyotus	2.8092E+11	280920.02
711	Stylodipus andrewsi	2.80808E+11	280808.29
712	Myotis aurascens	2.79799E+11	279799.04
713	Micronycteris hirsuta	2.79635E+11	279634.75
714	Petrodromus tetradactylus	2.79612E+11	279611.96
715	Sundamys muelleri	2.79106E+11	279106.45
716	Triaenops persicus	2.78971E+11	278970.90
717	Alticola stoliczkanus	2.78906E+11	278905.94
718	Condylura cristata	2.78612E+11	278611.87
719	Eptesicus ognevi	2.78116E+11	278116.30
720	Macaca leonina	2.7687E+11	276869.85
721	Plecotus ariel	2.75111E+11	275110.99
722	Nycteris tragata	2.74614E+11	274614.22
723	Echinosorex gymnura	2.73988E+11	273987.66
724	Viverra megaspila	2.7358E+11	273579.62
725	Histiotus montanus	2.7314E+11	273139.69
726	Glischropus tylopus	2.7299E+11	272990.41
727	Rhizomys sumatrensis	2.72778E+11	272778.24
728	Natalus mexicanus	2.7265E+11	272650.43
729	Paraechinus hypomelas	2.72387E+11	272386.88
730	Hylopetes spadiceus	2.72011E+11	272010.72
731	Scotophilus nigrita	2.71959E+11	271959.17
732	Tamandua mexicana	2.70852E+11	270851.91
733	Eozapus setchuanus	2.70725E+11	270724.92
734	Sturnira erythromos	2.70086E+11	270085.90
735	Murina hilgendorfi	2.69879E+11	269879.06

736	Microtus mystacinus	2.67288E+11	267287.78
737	Carollia castanea	2.66653E+11	266653.06
738	Mesophylla macconnelli	2.66531E+11	266531.37
739	Hypsignathus monstrosus	2.65492E+11	265492.36
740	Graphiurus murinus	2.65323E+11	265322.87
741	Iomys horsfieldii	2.64897E+11	264897.41
742	Sundasciurus lowii	2.64529E+11	264529.01
743	Aethomys hindei	2.64364E+11	264364.42
744	Ratufa affinis	2.64262E+11	264261.64
745	Mustela nudipes	2.64256E+11	264255.96
746	Raphicerus campestris	2.64005E+11	264004.64
747	Choeroniscus godmani	2.63949E+11	263949.19
748	Pipistrellus rusticus	2.63737E+11	263737.43
749	Macaca nemestrina	2.6349E+11	263489.85
750	Caryomys inez	2.63257E+11	263256.82
751	Hemigalus derbyanus	2.63242E+11	263242.17
752	Sicista subtilis	2.6312E+11	263120.24
753	Procapra gutturosa	2.62075E+11	262075.49
754	Galictis vittata	2.61413E+11	261412.61
755	Crocidura lamottei	2.61168E+11	261167.86
756	Hippotragus niger	2.61127E+11	261127.23
757	Bos gaurus	2.60976E+11	260975.76
758	Tragulus napu	2.60958E+11	260957.84
759	Suncus stoliczkanus	2.60837E+11	260837.36
760	Crocidura poensis	2.60683E+11	260682.80
761	Genetta servalina	2.5908E+11	259079.84
762	Plecotus strelkovi	2.5906E+11	259059.93
763	Maxomys whiteheadi	2.58708E+11	258708.23
764	Spermophilus pallidicauda	2.58436E+11	258435.96
765	Megaerops niphanae	2.5839E+11	258390.31
766	Lonchophylla thomasi	2.58168E+11	258168.38
767	Callosciurus prevostii	2.57985E+11	257984.69
768	Heliophobius argenteocinereus	2.56219E+11	256218.58
769	Maxomys rajah	2.55855E+11	255855.06
770	Sundasciurus hippurus	2.55624E+11	255623.67
771	Apodemus witherbyi	2.54857E+11	254857.19
772	Megaerops ecaudatus	2.54733E+11	254733.42
773	Myotis capaccinii	2.54007E+11	254007.44
774	Anomalurus beecrofti	2.53073E+11	253072.69
775	Handleyomys alfaroi	2.52955E+11	252954.83
776	Xerus rutilus	2.52175E+11	252174.56

777	Myotis leibii	2.5213E+11	252130.46
778	Caracal aurata	2.52093E+11	252093.42
779	Eothenomys miletus	2.51839E+11	251838.76
780	Myotis longicaudatus	2.51057E+11	251056.82
781	Lasiurus intermedius	2.49747E+11	249747.43
782	Miniopterus medius	2.49008E+11	249007.78
783	Reithrodontomys fulvescens	2.48913E+11	248913.02
784	Philander opossum	2.48091E+11	248091.00
785	Sundasciurus tenuis	2.47813E+11	247812.77
786	Spermophilus alashanicus	2.47649E+11	247649.08
787	Mogera robusta	2.46562E+11	246562.12
788	Allocricetulus eversmanni	2.4635E+11	246349.96
789	Rhinolophus trifoliatus	2.4561E+11	245610.24
790	Nycteris aurita	2.44716E+11	244716.26
791	Murina cyclotis	2.44393E+11	244392.59
792	Crocidura crossei	2.43542E+11	243542.41
793	Hipposideros megalotis	2.42635E+11	242634.63
794	Centronycteris centralis	2.4248E+11	242479.86
795	Taphozous perforatus	2.41962E+11	241961.87
796	Penthetor lucasi	2.41718E+11	241717.81
797	Ochotona pusilla	2.40714E+11	240713.70
798	Galago moholi	2.39364E+11	239364.15
799	Molossops temminckii	2.39282E+11	239281.59
800	Clethrionomys gapperi	2.39096E+11	239095.69
801	Lemniscomys rosalia	2.38498E+11	238498.00
802	Otomops harrisoni	2.38308E+11	238308.11
803	Ictonyx libycus	2.38135E+11	238134.53
804	Ellobius talpinus	2.38089E+11	238089.11
805	Conepatus semistriatus	2.37848E+11	237847.90
806	Sorex coronatus	2.3783E+11	237830.46
807	Cervus albirostris	2.37779E+11	237779.12
808	Kerivoula papillosa	2.37431E+11	237430.96
809	Cavia aperea	2.37144E+11	237144.33
810	Nannosciurus melanotis	2.36865E+11	236865.28
811	Crocidura fulvastra	2.36694E+11	236694.05
812	Tupaia minor	2.36196E+11	236196.35
813	Dendrohyrax dorsalis	2.35412E+11	235412.32
814	Lepus comus	2.35263E+11	235263.40
815	Myotis sodalis	2.34874E+11	234874.35
816	Rhinosciurus laticaudatus	2.34752E+11	234752.46
817	Eumops perotis	2.34278E+11	234277.72

818	Eumops glaucinus	2.33795E+11	233794.54
819	Paraechinus aethiopicus	2.3276E+11	232759.63
820	Myotis macrodactylus	2.32703E+11	232703.14
821	Alexandromys maximowiczii	2.32463E+11	232462.65
822	Erinaceus concolor	2.32187E+11	232186.93
823	Chlorocebus aethiops	2.32125E+11	232125.48
824	Glauconycteris poensis	2.31795E+11	231794.54
825	Aethomys kaiseri	2.31571E+11	231570.52
826	Hipposideros doriae	2.3133E+11	231329.56
827	Herpestes brachyurus	2.31247E+11	231246.86
828	Trachypithecus cristatus	2.31203E+11	231202.58
829	Graphiurus lorraineus	2.3076E+11	230760.32
830	Blarinella griselda	2.30404E+11	230403.57
831	Cercopithecus ascanius	2.30078E+11	230077.66
832	Conepatus chinga	2.28823E+11	228823.22
833	Cynomops greenhalli	2.28377E+11	228377.22
834	Phaiomys leucurus	2.27954E+11	227954.14
835	Plecotus kolombatovici	2.27672E+11	227672.05
836	Smutsia gigantea	2.27599E+11	227598.93
837	Anoura caudifer	2.27481E+11	227480.75
838	Chaerephon major	2.27463E+11	227463.38
839	Praomys tullbergi	2.2738E+11	227379.87
840	Tamandua tetradactyla	2.26849E+11	226849.00
841	Craseomys rufocanus	2.26809E+11	226809.00
842	Leopardus tigrinus	2.26554E+11	226554.38
843	Pteronotus gymnonotus	2.2578E+11	225779.94
844	Crocidura cyanea	2.25194E+11	225193.63
845	Lophostoma brasiliense	2.24981E+11	224980.88
846	Kerivoula furva	2.24697E+11	224696.54
847	Rhabdomys dilectus	2.24587E+11	224587.49
848	Mus macedonicus	2.24396E+11	224395.89
849	Mimon bennettii	2.24263E+11	224263.46
850	Myospalax aspalax	2.24026E+11	224025.93
851	Taphozous nudiventris	2.2298E+11	222980.36
852	Belomys pearsonii	2.22722E+11	222721.58
853	Petinomys genibarbis	2.22634E+11	222634.07
854	Thyroptera tricolor	2.22561E+11	222561.41
855	Menetes berdmorei	2.22154E+11	222153.70
856	Trichys fasciculata	2.21317E+11	221317.49
857	Elephantulus rufescens	2.21108E+11	221107.85
858	Cynogale bennettii	2.20901E+11	220901.09

859	Sicista concolor	2.20405E+11	220404.70
860	Hipposideros atrox	2.20147E+11	220146.91
861	Funisciurus pyrropus	2.1966E+11	219660.16
862	Leopardus colocolo	2.16889E+11	216888.80
863	Sturnira parvidens	2.15253E+11	215253.47
864	Crocidura voi	2.15117E+11	215117.13
865	Lonchophylla robusta	2.14746E+11	214746.09
866	Reithrodontomys megalotis	2.14495E+11	214495.47
867	Chaerephon bemmeleni	2.14494E+11	214494.03
868	Saccopteryx canescens	2.14469E+11	214468.69
869	Otonycteris hemprichii	2.1393E+11	213929.76
870	Lionycteris spurrelli	2.13659E+11	213659.04
871	Sorex longirostris	2.13183E+11	213183.20
872	Sigmodon hispidus	2.12011E+11	212011.17
873	Kerivoula titania	2.11206E+11	211205.65
874	Epomophorus crypturus	2.10983E+11	210983.23
875	Raphicerus sharpei	2.10966E+11	210965.92
876	Eumops ferox	2.10965E+11	210965.02
877	Conepatus leuconotus	2.09172E+11	209172.38
878	Myomyscus brockmani	2.09159E+11	209159.37
879	Sorex bedfordiae	2.08792E+11	208791.83
880	Sciurus granatensis	2.08414E+11	208413.58
881	Hylochoerus meinertzhageni	2.08135E+11	208134.87
882	Mops thersites	2.08097E+11	208096.66
883	Rhinolophus eloquens	2.07918E+11	207918.41
884	Mephitis macroura	2.07783E+11	207782.58
885	Cynomops planirostris	2.07331E+11	207331.46
886	Phyllostomus elongatus	2.07016E+11	207015.98
887	Cynomops abrasus	2.06516E+11	206515.96
888	Micronycteris microtis	2.06114E+11	206113.62
889	Steatomys pratensis	2.05553E+11	205553.31
890	Lophostoma silvicolum	2.04995E+11	204995.46
891	Sigmodon hirsutus	2.0462E+11	204619.81
892	Salpingotus crassicauda	2.04472E+11	204472.33
893	Rhynchogale melleri	2.04298E+11	204298.32
894	Nycteris gambiensis	2.04248E+11	204247.74
895	Arvicanthis rufinus	2.04007E+11	204007.42
896	Herpestes semitorquatus	2.03937E+11	203936.74
897	Spilogale angustifrons	2.03665E+11	203665.08
898	Mus platythrix	2.03632E+11	203631.71
899	Cercopithecus mona	2.03482E+11	203481.88

900	Mylomys dybowskii	2.03215E+11	203215.10
901	Megaloglossus woermanni	2.032E+11	203200.04
902	Mazama gouazoubira	2.03073E+11	203072.67
903	Euphractus sexcinctus	2.0235E+11	202349.82
904	Sturnira tildae	2.0217E+11	202170.48
905	Equus quagga	2.01034E+11	201033.82
906	Hylopetes platyurus	2.00963E+11	200962.75
907	Salpingotus kozlovi	2.00852E+11	200851.69
908	Mus spretus	2.00429E+11	200429.05
909	Miniopterus fraterculus	2.00144E+11	200144.37
910	Hipposideros bicolor	1.99839E+11	199839.13
911	Papio ursinus	1.99303E+11	199303.05
912	Sorex fumeus	1.98375E+11	198374.61
913	Murina ussuriensis	1.98143E+11	198143.38
914	Promops nasutus	1.97582E+11	197581.84
915	Mops brachypterus	1.96585E+11	196585.09
916	Neotoma floridana	1.96559E+11	196559.18
917	Spilogale putorius	1.96452E+11	196451.79
918	Balantiopteryx plicata	1.96202E+11	196202.30
919	Ochotona rufescens	1.95534E+11	195533.64
920	Cephalophus dorsalis	1.95491E+11	195491.12
921	Redunca fulvorufula	1.95435E+11	195434.84
922	Gerbilliscus boehmi	1.95372E+11	195372.46
923	Epomops buettikoferi	1.95327E+11	195327.49
924	Rhogeessa io	1.94816E+11	194815.84
925	Didelphis pernigra	1.94522E+11	194522.22
926	Hesperoptenus tickelli	1.93298E+11	193297.79
927	Bassariscus astutus	1.92841E+11	192840.69
928	Pipistrellus stenopterus	1.92485E+11	192484.77
929	Micaelamys namaquensis	1.91912E+11	191912.48
930	Papio cynocephalus	1.91906E+11	191906.28
931	Murina huttoni	1.91628E+11	191628.48
932	Arvicola scherman	1.91587E+11	191586.93
933	Molossus coibensis	1.91215E+11	191215.07
934	Dicerorhinus sumatrensis	1.91211E+11	191211.03
935	Mops nanulus	1.90969E+11	190968.60
936	Mus setulosus	1.90813E+11	190812.50
937	Euchoreutes naso	1.90741E+11	190741.21
938	Mus cookii	1.90654E+11	190654.31
939	Alexandromys mongolicus	1.90517E+11	190517.30
940	Petinomys setosus	1.89814E+11	189814.12

941	Antrozous pallidus	1.89525E+11	189525.21
942	Paraxerus cepapi	1.88459E+11	188458.61
943	Craseomys shanseius	1.87447E+11	187446.68
944	Vulpes pallida	1.86872E+11	186871.98
945	Graphiurus nagtglasii	1.86774E+11	186774.14
946	Loris lydekkerianus	1.86116E+11	186116.27
947	Sturnira ludovici	1.85742E+11	185741.84
948	Molossus pretiosus	1.85524E+11	185524.19
949	Spermophilus fulvus	1.84976E+11	184975.58
950	Marmosa mexicana	1.84488E+11	184488.27
951	Didelphis albiventris	1.84107E+11	184107.08
952	Rhinolophus alcyone	1.83824E+11	183823.84
953	Apodemus latronum	1.83821E+11	183821.06
954	Sorex thibetanus	1.83593E+11	183592.77
955	Myotis badius	1.83415E+11	183414.63
956	Rhinolophus schnitzleri	1.83415E+11	183414.63
957	Molossus bondae	1.82962E+11	182962.29
958	Suncus varilla	1.82675E+11	182675.49
959	Dremomys pyrrhomerus	1.82593E+11	182593.31
960	Chiroderma trinitatum	1.823E+11	182300.12
961	Crocidura roosevelti	1.82285E+11	182285.37
962	Apodemus pallipes	1.82269E+11	182268.62
963	Atelerix algirus	1.81922E+11	181921.70
964	Artibeus obscurus	1.81699E+11	181699.34
965	Tupaia tana	1.81285E+11	181285.40
966	Glischropus bucephalus	1.80886E+11	180885.84
967	Nycteris nana	1.80681E+11	180680.52
968	Pipistrellus paterculus	1.80358E+11	180357.69
969	Myotis californicus	1.80065E+11	180064.97
970	Lophuromys flavopunctatus	1.7971E+11	179710.19
971	Crocidura foxi	1.79692E+11	179691.86
972	Corynorhinus townsendii	1.79674E+11	179674.43
973	Thyroptera discifera	1.79477E+11	179477.13
974	Ateles geoffroyi	1.79321E+11	179321.09
975	Ovis vignei	1.79318E+11	179317.56
976	Hipposideros jonesi	1.78746E+11	178746.46
977	Gerbillus henleyi	1.78729E+11	178729.04
978	Phataginus tetradactyla	1.78506E+11	178506.25
979	Corynorhinus rafinesquii	1.78377E+11	178377.18
980	Ictidomys tridecemlineatus	1.78289E+11	178289.07
981	Ochrotomys nuttalli	1.77914E+11	177914.31

982	Reithrodontomys humulis	1.77717E+11	177717.09
983	Dendromus nyikae	1.77688E+11	177688.04
984	Lepus mandshuricus	1.76862E+11	176861.91
985	Choloepus hoffmanni	1.76342E+11	176341.61
986	Mus mahomet	1.76152E+11	176151.66
987	Miniopterus inflatus	1.76114E+11	176113.68
988	Emballonura alecto	1.75652E+11	175652.17
989	Acomys wilsoni	1.75469E+11	175468.92
990	Bdeogale crassicauda	1.74945E+11	174944.92
991	Uroderma bilobatum	1.74803E+11	174803.45
992	Mus haussa	1.74575E+11	174575.24
993	Platyrrhinus dorsalis	1.7451E+11	174510.41
994	Oecomys trinitatis	1.74447E+11	174447.03
995	Crocidura dolichura	1.74436E+11	174435.65
996	Rhinolophus siamensis	1.74292E+11	174292.10
997	Pedetes capensis	1.73855E+11	173855.25
998	Reithrodontomys mexicanus	1.73505E+11	173505.45
999	Lycalopex culpaeus	1.73232E+11	173232.36
1000	Nectogale elegans	1.73226E+11	173225.84
1001	Microtus ochrogaster	1.72553E+11	172553.16
1002	Capra aegagrus	1.72321E+11	172321.36
1003	Eumops underwoodi	1.71711E+11	171710.78
1004	Hipposideros lylei	1.71612E+11	171612.21
1005	Melanomys caliginosus	1.70678E+11	170678.45
1006	Chlorocebus cynosuros	1.70427E+11	170426.74
1007	Meriones tristrami	1.70216E+11	170216.24
1008	Carollia sowelli	1.69025E+11	169024.62
1009	Arvicanthis neumanni	1.68891E+11	168890.84
1010	Chodsigoa hypsibia	1.68831E+11	168831.03
1011	Elephas maximus	1.68759E+11	168759.13
1012	Eptesicus isabellinus	1.68619E+11	168618.52
1013	Eliomys munbyanus	1.6853E+11	168529.74
1014	Choeronycteris mexicana	1.68372E+11	168372.38
1015	Miniopterus pusillus	1.68298E+11	168297.75
1016	Coendou mexicanus	1.68083E+11	168083.40
1017	Nesotragus moschatus	1.68027E+11	168027.03
1018	Rhinolophus swinnyi	1.6736E+11	167359.87
1019	Plecotus macrobullaris	1.67138E+11	167137.78
1020	Crocidura whitakeri	1.66566E+11	166566.06
1021	Perodicticus potto	1.66365E+11	166365.25
1022	Lemniscomys macculus	1.66269E+11	166268.90

1023	Nycteris intermedia	1.6625E+11	166250.10
1024	Eptesicus hottentotus	1.66063E+11	166062.70
1025	Ochotona gloveri	1.65977E+11	165977.49
1026	Lepus habessinicus	1.65627E+11	165627.39
1027	Scapanulus oweni	1.65623E+11	165623.01
1028	Myotis thysanodes	1.65519E+11	165519.46
1029	Myotis volans	1.65502E+11	165501.79
1030	Cephalophus niger	1.64921E+11	164920.87
1031	Ochotona erythrotis	1.6483E+11	164830.17
1032	Vampyrodes major	1.64794E+11	164793.83
1033	Dermanura tolteca	1.64742E+11	164742.27
1034	Sicista betulina	1.64283E+11	164283.45
1035	Myonycteris leptodon	1.64077E+11	164076.97
1036	Bandicota savilei	1.63791E+11	163790.70
1037	Steatomys parvus	1.63643E+11	163643.44
1038	Nyctomys sumichrasti	1.63405E+11	163405.11
1039	Anoura peruana	1.62889E+11	162888.52
1040	Gerbillus campestris	1.62509E+11	162508.72
1041	Equus kiang	1.62331E+11	162330.64
1042	Stenocephalemys albipes	1.62152E+11	162151.84
1043	Madoqua kirkii	1.60057E+11	160056.84
1044	Trachypithecus francoisi	1.59791E+11	159790.51
1045	Eptesicus bottae	1.5952E+11	159519.51
1046	Sturnira lilium	1.58857E+11	158857.18
1047	Dermanura gnoma	1.58501E+11	158501.32
1048	Pardofelis marmorata	1.58192E+11	158192.17
1049	Scotonycteris bergmansi	1.57338E+11	157338.33
1050	Rhinolophus celebensis	1.5715E+11	157149.87
1051	Erethizon dorsatum	1.56541E+11	156541.13
1052	Tonatia saurophila	1.56487E+11	156487.46
1053	Mazama temama	1.56222E+11	156221.95
1054	Apodemus argenteus	1.55979E+11	155978.73
1055	Apodemus speciosus	1.55975E+11	155974.95
1056	Trinycteris nicefori	1.55967E+11	155967.26
1057	Caluromys derbianus	1.55763E+11	155763.13
1058	Myotis punicus	1.55718E+11	155717.94
1059	Meriones grandis	1.55609E+11	155608.72
1060	Sciurus deppei	1.5549E+11	155489.71
1061	Mustela itatsi	1.55451E+11	155451.07
1062	Anoura cultrata	1.54789E+11	154788.69
1063	Rhinolophus darlingi	1.54171E+11	154171.33

1064	Eptesicus andinus	1.53978E+11	153978.12
1065	Neotoma mexicana	1.53633E+11	153632.64
1066	Myocastor coypus	1.53277E+11	153276.90
1067	Parascalops breweri	1.52948E+11	152947.67
1068	Semnopithecus hypoleucos	1.5277E+11	152770.03
1069	Myotis peytoni	1.52664E+11	152664.29
1070	Necromys lasiurus	1.51684E+11	151684.48
1071	Lepus californicus	1.51678E+11	151677.97
1072	Callosciurus pygerythrus	1.51387E+11	151387.11
1073	Hystrix sumatrae	1.51303E+11	151302.83
1074	Dyacopterus brooksi	1.51303E+11	151302.69
1075	Tupaia ferruginea	1.51303E+11	151302.52
1076	Nycticebus coucang	1.51017E+11	151017.15
1077	Leptonycteris yerbabuenae	1.50845E+11	150845.24
1078	Alouatta seniculus	1.506E+11	150600.15
1079	Paraxerus palliatus	1.50043E+11	150043.03
1080	Paraxerus boehmi	1.49989E+11	149988.71
1081	Artibeus planirostris	1.49617E+11	149616.69
1082	Caluromys lanatus	1.494E+11	149400.03
1083	Crocidura dsinezumi	1.49265E+11	149264.71
1084	Cremnomys cutchicus	1.49252E+11	149251.56
1085	Vulpes rueppellii	1.49231E+11	149230.94
1086	Sturnira bidens	1.49043E+11	149043.08
1087	Pipistrellus imbricatus	1.48746E+11	148746.31
1088	Crocidura mariquensis	1.48144E+11	148144.33
1089	Glossophaga leachii	1.47898E+11	147898.41
1090	Urotrichus talpoides	1.47779E+11	147779.48
1091	Lepus brachyurus	1.4764E+11	147639.82
1092	Lasiurus egregius	1.47346E+11	147346.45
1093	Tupaia javanica	1.47105E+11	147105.17
1094	Macrotus waterhousii	1.46705E+11	146705.10
1095	Stochomys longicaudatus	1.46676E+11	146675.87
1096	Crocidura maxi	1.4662E+11	146619.92
1097	Lasiurus seminolus	1.46382E+11	146381.74
1098	Marmosa demerarae	1.46307E+11	146307.29
1099	Hipposideros caffer	1.46241E+11	146240.70
1100	Rhogeessa tumida	1.46237E+11	146236.67
1101	Myotis velifer	1.46172E+11	146171.63
1102	Gerbillus dasyurus	1.45619E+11	145619.14
1103	Myopterus daubentonii	1.45443E+11	145442.77
1104	Rhinolophus bocharicus	1.45192E+11	145192.37

1105	Lepus americanus	1.44892E+11	144891.92
1106	Tylomys nudicaudus	1.44812E+11	144811.64
1107	Eolagurus przewalskii	1.44757E+11	144756.59
1108	Saccolaimus peli	1.446E+11	144600.12
1109	Rhyneptesicus nasutus	1.44217E+11	144216.98
1110	Funisciurus anerythrus	1.44216E+11	144215.82
1111	Dyacopterus spadiceus	1.43842E+11	143842.39
1112	Sylvisorex johnstoni	1.43767E+11	143767.15
1113	Sciurus aestuans	1.43623E+11	143623.12
1114	Alouatta juara	1.43418E+11	143418.27
1115	Rhinophylla pumilio	1.42704E+11	142704.39
1116	Oligoryzomys flavescens	1.42552E+11	142552.33
1117	Oligoryzomys nigripes	1.42509E+11	142508.97
1118	Mus cervicolor	1.42476E+11	142475.66
1119	Graphiurus crassicaudatus	1.42189E+11	142189.22
1120	Philetor brachypterus	1.42066E+11	142066.03
1121	Pachyuromys duprasi	1.41901E+11	141901.12
1122	Sturnira bogotensis	1.41862E+11	141862.19
1123	Hylobates agilis	1.41421E+11	141420.87
1124	Lepus peguensis	1.41132E+11	141132.38
1125	Crocidura littoralis	1.40222E+11	140222.38
1126	Artibeus amplus	1.39878E+11	139877.94
1127	Oecomys catherinae	1.39635E+11	139635.23
1128	Mops demonstrator	1.39266E+11	139265.66
1129	Episoriculus macrurus	1.39232E+11	139231.73
1130	Lichonycteris obscura	1.39015E+11	139015.33
1131	Pteronotus mesoamericanus	1.38602E+11	138602.07
1132	Genetta pardina	1.38394E+11	138393.65
1133	Glyphonycteris sylvestris	1.38094E+11	138093.68
1134	Hipposideros beatus	1.37809E+11	137809.29
1135	Mesechinus hughi	1.37798E+11	137797.79
1136	Hylopetes phayrei	1.37503E+11	137503.40
1137	Miniopterus tristis	1.37435E+11	137435.15
1138	Rhinolophus mossambicus	1.37391E+11	137391.42
1139	Marmota caudata	1.37358E+11	137358.48
1140	Hystrix javanica	1.37161E+11	137161.38
1141	Perodicticus ibeanus	1.37007E+11	137006.76
1142	Nasua nasua	1.36064E+11	136064.34
1143	Choeroniscus minor	1.35692E+11	135691.82
1144	Euroscaptor longirostris	1.35621E+11	135620.53
1145	Phodopus sungorus	1.3535E+11	135350.29

1146	Hylomyscus aeta	1.35271E+11	135271.18
1147	Bassariscus sumichrasti	1.35234E+11	135233.73
1148	Miniopterus shortridgei	1.35059E+11	135058.96
1149	Dasypus septemcinctus	1.34869E+11	134868.76
1150	Sorex palustris	1.34832E+11	134831.73
1151	Crocidura planiceps	1.34626E+11	134625.80
1152	Stylodipus telum	1.34347E+11	134347.09
1153	Myotis yumanensis	1.34066E+11	134066.41
1154	Sturnira hondurensis	1.33994E+11	133993.52
1155	Rhinolophus marshalli	1.33852E+11	133851.74
1156	Sciurotamias forresti	1.33501E+11	133500.67
1157	Arvicanthis abyssinicus	1.33352E+11	133352.47
1158	Tragelaphus imberbis	1.33161E+11	133161.15
1159	Alticola strelzowi	1.33078E+11	133078.39
1160	Cephalophus nigrifrons	1.33017E+11	133016.56
1161	Glaucomys sabrinus	1.329E+11	132900.22
1162	Scarturus williamsi	1.32249E+11	132249.27
1163	Myonycteris torquata	1.32071E+11	132070.63
1164	Acomys dimidiatus	1.31815E+11	131815.11
1165	Martes melampus	1.31483E+11	131483.18
1166	Diplomesodon pulchellus	1.3088E+11	130880.45
1167	Neoromicia helios	1.3076E+11	130759.86
1168	Peromyscus gossypinus	1.30553E+11	130553.20
1169	Hylobates lar	1.30521E+11	130520.87
1170	Cervus nippon	1.3043E+11	130430.23
1171	Sorex hoyi	1.30344E+11	130344.18
1172	Genetta abyssinica	1.30232E+11	130231.80
1173	Dinomys branickii	1.30059E+11	130059.27
1174	Anoura latidens	1.29311E+11	129310.71
1175	Chionomys syriacus	1.29257E+11	129256.62
1176	Ratufa indica	1.29139E+11	129139.00
1177	Myotis melanorhinus	1.28744E+11	128743.64
1178	Lichonycteris degener	1.28305E+11	128304.58
1179	Hapalomys delacouri	1.28251E+11	128251.03
1180	Papio hamadryas	1.28E+11	128000.34
1181	Pipistrellus anchietae	1.27974E+11	127973.51
1182	Cannomys badius	1.27861E+11	127860.80
1183	Connochaetes taurinus	1.27545E+11	127545.12
1184	Plecotus homochrous	1.2754E+11	127539.54
1185	Steatomys caurinus	1.26676E+11	126675.67
1186	Thomasomys aureus	1.26561E+11	126561.07

1187	Nycteris javanica	1.26416E+11	126416.40
1188	Cabassous centralis	1.26154E+11	126153.94
1189	Pygoderma bilabiatum	1.25992E+11	125992.41
1190	Mops niveiventer	1.25934E+11	125933.83
1191	Papio kindae	1.25287E+11	125287.38
1192	Trachypithecus phayrei	1.25243E+11	125243.16
1193	Cynomops paranus	1.25134E+11	125133.91
1194	Ochotona hyperborea	1.24995E+11	124995.34
1195	Alexandromys montebelli	1.2492E+11	124920.43
1196	Heteromys desmarestianus	1.2483E+11	124830.06
1197	Martes zibellina	1.24271E+11	124270.58
1198	Symphalangus syndactylus	1.23983E+11	123983.33
1199	Callosciurus finlaysonii	1.23862E+11	123861.66
1200	Pteropus hypomelanus	1.23809E+11	123808.88
1201	Petaurista leucogenys	1.23424E+11	123424.28
1202	Funisciurus leucogenys	1.23219E+11	123219.05
1203	Kerivoula depressa	1.23177E+11	123176.85
1204	Cheiromeles parvidens	1.22693E+11	122692.68
1205	Cynopterus luzoniensis	1.2264E+11	122639.74
1206	Dermanura azteca	1.21677E+11	121677.16
1207	Murina aurata	1.21333E+11	121333.14
1208	Calomyscus baluchi	1.20842E+11	120841.73
1209	Crocidura nigeriae	1.20842E+11	120841.71
1210	Vampyriscus bidens	1.20819E+11	120819.02
1211	Spermophilus pygmaeus	1.20651E+11	120650.81
1212	Baiomys musculus	1.2064E+11	120640.07
1213	Melogale orientalis	1.19784E+11	119783.87
1214	Heliosciurus mutabilis	1.19617E+11	119617.03
1215	Crocidura indochinensis	1.19607E+11	119607.47
1216	Paraxerus ochraceus	1.19364E+11	119363.99
1217	Scotoecus pallidus	1.18969E+11	118968.56
1218	Hylopetes sagitta	1.18954E+11	118954.04
1219	Mus tenellus	1.18603E+11	118602.52
1220	Marmota bobak	1.18214E+11	118214.07
1221	Odocoileus hemionus	1.17873E+11	117873.25
1222	Callosciurus caniceps	1.17838E+11	117838.20
1223	Histiotus velatus	1.17651E+11	117650.84
1224	Elephantulus rozeti	1.17536E+11	117535.84
1225	Talpa levantis	1.1751E+11	117509.73
1226	Myotis elegans	1.17477E+11	117477.01
1227	Mimon cozumelae	1.17426E+11	117425.77

1228	Microtus afghanus	1.16663E+11	116663.41
1229	Alouatta palliata	1.16465E+11	116464.90
1230	Sciurus aureogaster	1.16271E+11	116271.32
1231	Perodicticus edwardsi	1.15924E+11	115924.50
1232	Myotis rosseti	1.1574E+11	115740.34
1233	Falsistrellus mordax	1.15683E+11	115682.63
1234	Ametrida centurio	1.1566E+11	115660.17
1235	Microryzomys minutus	1.15613E+11	115613.02
1236	Rhinoceros sondaicus	1.15607E+11	115607.27
1237	Vernaya fulva	1.15326E+11	115325.87
1238	Sphaerias blanfordi	1.15276E+11	115275.91
1239	Hylomyscus alleni	1.15152E+11	115152.46
1240	Pteromyscus pulverulentus	1.15138E+11	115138.00
1241	Lophiomys imhausi	1.15035E+11	115034.81
1242	Dermanura cinerea	1.15021E+11	115020.55
1243	Rhinopithecus roxellana	1.14935E+11	114935.31
1244	Praomys derooi	1.1479E+11	114790.40
1245	Coendou quichua	1.14219E+11	114219.49
1246	Marmosa murina	1.14218E+11	114218.07
1247	Napaeozapus insignis	1.1408E+11	114080.39
1248	Myotis rufoniger	1.13918E+11	113917.53
1249	Hypsugo dolichodon	1.13894E+11	113894.21
1250	Platyrrhinus vittatus	1.13861E+11	113861.23
1251	Plecotus kozlovi	1.13761E+11	113760.78
1252	Trachypithecus pileatus	1.13394E+11	113393.58
1253	Eptesicus diminutus	1.13359E+11	113358.75
1254	Rhinophylla fischerae	1.13077E+11	113077.07
1255	Sylvilagus gabbi	1.12374E+11	112373.92
1256	Paraxerus poensis	1.12142E+11	112142.48
1257	Psammomys obesus	1.12118E+11	112117.91
1258	Chlorocebus sabaeus	1.12062E+11	112062.46
1259	Paracynictis selousi	1.12031E+11	112031.14
1260	Coendou rufescens	1.11713E+11	111713.12
1261	Ochotona thomasi	1.11583E+11	111583.22
1262	Heterocephalus glaber	1.11271E+11	111270.60
1263	Cercopithecus nictitans	1.11163E+11	111163.25
1264	Sciurus variegatoides	1.1113E+11	111129.99
1265	Eumops hansae	1.10238E+11	110237.51
1266	Maxomys tajuddinii	1.10178E+11	110178.47
1267	Artibeus fimbriatus	1.10104E+11	110104.33
1268	Hybomys univittatus	1.09724E+11	109723.83

1269	Panthera onca	1.09597E+11	109596.64
1270	Colobus vellerosus	1.09166E+11	109166.41
1271	Monodelphis americana	1.0895E+11	108949.80
1272	Felis margarita	1.08907E+11	108907.38
1273	Trachypithecus auratus	1.08689E+11	108689.30
1274	Colobus angolensis	1.08531E+11	108531.18
1275	Chiroderma doriae	1.08371E+11	108371.35
1276	Leopardus geoffroyi	1.07973E+11	107973.08
1277	Ochotona alpina	1.07967E+11	107966.56
1278	Rhinolophus beddomei	1.07773E+11	107772.97
1279	Neoromicia melckorum	1.07585E+11	107585.48
1280	Malacomys edwardsi	1.07394E+11	107393.74
1281	Mesocricetus brandti	1.07227E+11	107227.46
1282	Leptonycteris curasoae	1.07224E+11	107224.17
1283	Meriones shawi	1.07219E+11	107218.55
1284	Nycticebus pygmaeus	1.07149E+11	107149.36
1285	Hyemoschus aquaticus	1.07098E+11	107097.67
1286	Malacomys longipes	1.07059E+11	107058.61
1287	Gazella dorcas	1.06748E+11	106748.32
1288	Myotis grisescens	1.06732E+11	106731.55
1289	Peromyscus mexicanus	1.06481E+11	106480.55
1290	Lophocebus albigena	1.063E+11	106300.03
1291	Lutreolina crassicaudata	1.05646E+11	105646.18
1292	Ototylomys phyllotis	1.05635E+11	105635.06
1293	Carollia subrufa	1.0563E+11	105629.73
1294	Monodelphis dimidiata	1.05207E+11	105207.15
1295	Mus spicilegus	1.05187E+11	105186.51
1296	Hoolock hoolock	1.0518E+11	105180.03
1297	Pygeretmus platyurus	1.0512E+11	105119.87
1298	Sciurus lis	1.04975E+11	104975.19
1299	Leptonycteris nivalis	1.04938E+11	104938.07
1300	Cephalophus weynsi	1.04679E+11	104679.20
1301	Otomys angoniensis	1.04533E+11	104532.91
1302	Otospermophilus variegatus	1.04334E+11	104333.56
1303	Tragelaphus eurycerus	1.04226E+11	104226.30
1304	Jaculus orientalis	1.04193E+11	104193.12
1305	Madoqua guentheri	1.03774E+11	103773.77
1306	Reithrodontomys sumichrasti	1.03375E+11	103375.29
1307	Epomops dobsonii	1.0294E+11	102940.27
1308	Lasiurus xanthinus	1.02838E+11	102837.63
1309	Apodemus mystacinus	1.02821E+11	102820.72

1310	Cricetulus sokolovi	1.02406E+11	102405.77
1311	Rhynchocyon cirnei	1.02391E+11	102390.61
1312	Spilogale gracilis	1.02248E+11	102248.37
1313	Litocranius walleri	1.02036E+11	102035.81
1314	Semnopithecus schistaceus	1.02032E+11	102032.23
1315	Crossarchus platycephalus	1.02002E+11	102002.15
1316	Pipistrellus crassulus	1.01771E+11	101771.43
1317	Myotis alcathoe	1.01545E+11	101544.82
1318	Lepus coreanus	1.0104E+11	101040.15
1319	Cephalopachus bancanus	1.01011E+11	101011.44
1320	Cabassous unicinctus	1.00669E+11	100669.36
1321	Vampyrodes caraccioli	1.00632E+11	100632.34
1322	Cabassous tatouay	1.00348E+11	100347.52
1323	Loxodonta africana	1.0025E+11	100249.57
1324	Mops trevori	1.00095E+11	100094.78
1325	Pan troglodytes	99939211404	99939.21
1326	Aeromys tephromelas	99935256556	99935.26
1327	Tamiops rodolphii	99880761042	99880.76
1328	Nephelomys albigularis	99779249029	99779.25
1329	Sylvilagus audubonii	99560965280	99560.97
1330	Platyrrhinus infuscus	99350090714	99350.09
1331	Cansumys canus	99240425322	99240.43
1332	Oligoryzomys eliurus	99004586404	99004.59
1333	Lemniscomys barbarus	98775547080	98775.55
1334	Hesperoptenus blanfordi	98002540160	98002.54
1335	Baiomys taylori	97959852423	97959.85
1336	Suncus lixus	97934034164	97934.03
1337	Sorex cylindricauda	97845028914	97845.03
1338	Niviventer niviventer	97186006442	97186.01
1339	Budorcas taxicolor	97129777073	97129.78
1340	Gerbillus cheesmani	97004504836	97004.50
1341	Marmota baibacina	96778500812	96778.50
1342	Kerivoula phalaena	96735928520	96735.93
1343	Rousettus lanosus	96280436762	96280.44
1344	Scotophilus nux	96183689148	96183.69
1345	Philantomba maxwellii	96143323584	96143.32
1346	Eothenomys olitor	95937819939	95937.82
1347	Pedetes surdaster	95384380708	95384.38
1348	Spermophilopsis leptodactylus	95327516312	95327.52
1349	Caluromys philander	95058315296	95058.32
1350	Handleyomys rostratus	94990868450	94990.87

1351	Glossophaga longirostris	94971750477	94971.75
1352	Spermophilus citellus	94618093195	94618.09
1353	Nannospalax leucodon	94546075322	94546.08
1354	Kerivoula intermedia	94486729152	94486.73
1355	Gerbilliscus brantsii	94242544167	94242.54
1356	Acomys cineraceus	93841574858	93841.57
1357	Muntiacus rooseveltorum	93690057107	93690.06
1358	Scotophilus collinus	93491683173	93491.68
1359	Glirulus japonicus	93455874214	93455.87
1360	Eospalax rothschildi	93454875727	93454.88
1361	Meles anakuma	92920726130	92920.73
1362	Myotis ruber	92710160020	92710.16
1363	Didelphis aurita	92666355812	92666.36
1364	Natalus espiritosantensis	92610182173	92610.18
1365	Chrysocyon brachyurus	92560623355	92560.62
1366	Molossops mattogrossensis	92529416877	92529.42
1367	Parastrellus hesperus	92498344686	92498.34
1368	Myotis ridleyi	92134497985	92134.50
1369	Hybomys trivirgatus	91856656412	91856.66
1370	Parascaptor leucura	91734170998	91734.17
1371	Philantomba walteri	91574707477	91574.71
1372	Blarina carolinensis	91442645539	91442.65
1373	Sciurus pucheranii	91381625936	91381.63
1374	Panthera leo	91264812804	91264.81
1375	Steatomys cuppedius	90718038506	90718.04
1376	Saccostomus mearnsi	90706833445	90706.83
1377	Neotetracus sinensis	90634372326	90634.37
1378	Heteromys salvini	90275430279	90275.43
1379	Leopardus guttulus	90131971849	90131.97
1380	Genetta angolensis	90112588600	90112.59
1381	Dasyprocta leporina	90072721275	90072.72
1382	Tadarida latouchei	89685331273	89685.33
1383	Crocidura rapax	89509369814	89509.37
1384	Allactaga severtzovi	89125756861	89125.76
1385	Gazella marica	89023809972	89023.81
1386	Episoriculus caudatus	89014978080	89014.98
1387	Idionycteris phyllotis	88923031723	88923.03
1388	Epomophorus minimus	88908842122	88908.84
1389	Eumops dabbenei	88876663876	88876.66
1390	Rhinolophus arcuatus	88758271639	88758.27
1391	Plecotus wardi	88669567740	88669.57

1392	Mus phillipsi	88661132968	88661.13
1393	Pipistrellus inexspectatus	88631826025	88631.83
1394	Sylvilagus aquaticus	88441517585	88441.52
1395	Funisciurus congicus	88192589778	88192.59
1396	Elephantulus myurus	88127929710	88127.93
1397	Platyrrhinus recifinus	88041832675	88041.83
1398	Lagidium viscacia	88032371566	88032.37
1399	Reithrodontomys gracilis	87835152840	87835.15
1400	Otomys auratus	87824807446	87824.81
1401	Cercopithecus neglectus	87735301405	87735.30
1402	Heteromys irroratus	87025939609	87025.94
1403	Sooretamys angouya	86654596486	86654.60
1404	Nasuella olivacea	86453590971	86453.59
1405	Gerbillus pusillus	86339062205	86339.06
1406	Myotis evotis	86153268726	86153.27
1407	Microtus guentheri	86129689100	86129.69
1408	Praomys rostratus	86120872354	86120.87
1409	Caprolagus hispidus	86043637748	86043.64
1410	Chaetodipus hispidus	86020734196	86020.73
1411	Cuniculus taczanowskii	86007267788	86007.27
1412	Idiurus macrotis	85923177249	85923.18
1413	Platyrrhinus angustirostris	85917927253	85917.93
1414	Alticola barakshin	85791967160	85791.97
1415	Ellobius lutescens	85789769795	85789.77
1416	Microtus juldaschi	85710927081	85710.93
1417	Hipposideros obscurus	85696831744	85696.83
1418	Myotis macrotarsus	85599081434	85599.08
1419	Nanger granti	85573547689	85573.55
1420	Herpestes naso	85527095339	85527.10
1421	Giraffa camelopardalis	85509031755	85509.03
1422	Ptenochirus jagori	85261130971	85261.13
1423	Meriones hurrianae	85227947211	85227.95
1424	Cavia tschudii	85114915428	85114.92
1425	Otomys tropicalis	85110165467	85110.17
1426	Haplonycteris fischeri	84878339991	84878.34
1427	Spermophilus brevicauda	84756773846	84756.77
1428	Miniopterus paululus	84693629093	84693.63
1429	Glauconycteris beatrix	84690202774	84690.20
1430	Rhinolophus virgo	84586679911	84586.68
1431	Cephalophus harveyi	84469005342	84469.01
1432	Sciurus anomalus	83962036870	83962.04

1433	Crocidura serezkyensis	83762293010	83762.29
1434	Crocidura lepidura	83715957480	83715.96
1435	Sphaeronycteris toxophyllum	83625383987	83625.38
1436	Dendromus mesomelas	83596209171	83596.21
1437	Thainycteris aureocollaris	83552200447	83552.20
1438	Acomys cahirinus	83504565235	83504.57
1439	Cephalophus natalensis	83322320081	83322.32
1440	Mops spurrelli	82782288913	82782.29
1441	Ochotona nubrica	82755183087	82755.18
1442	Centronycteris maximiliani	81827671891	81827.67
1443	Gerbillus gerbillus	81430916659	81430.92
1444	Semnopithecus priam	81156256069	81156.26
1445	Eptesicus anatolicus	81009776894	81009.78
1446	Rhogeessa minutilla	80961650652	80961.65
1447	Hypsugo affinis	80933810782	80933.81
1448	Arctonyx hoevenii	80896925839	80896.93
1449	Cercopithecus petaurista	80722261836	80722.26
1450	Lampronycteris brachyotis	80684150764	80684.15
1451	Transandinomys talamancae	80519529749	80519.53
1452	Cormura brevirostris	80325177619	80325.18
1453	Myotis bombinus	80241820545	80241.82
1454	Nannospalax ehrenbergi	80185429204	80185.43
1455	Acomys russatus	80151353567	80151.35
1456	Vampyressa pusilla	79903508731	79903.51
1457	Ellobius fuscocapillus	79750231880	79750.23
1458	Oryctolagus cuniculus	79717931353	79717.93
1459	Coendou spinosus	79187339925	79187.34
1460	Ovis gmelini	79087277835	79087.28
1461	Peromyscus aztecus	79062433292	79062.43
1462	Oecomys bicolor	78920515454	78920.52
1463	Lepus fagani	78847353746	78847.35
1464	Vulpes chama	78682658595	78682.66
1465	Rhinolophus coelophyllus	78677415222	78677.42
1466	Mastomys coucha	78432914659	78432.91
1467	Marmosops caucae	78403515758	78403.52
1468	Tonatia bidens	78396177649	78396.18
1469	Corynorhinus mexicanus	78203726745	78203.73
1470	Kerivoula kachinensis	78179095418	78179.10
1471	Myotis scotti	78119625040	78119.63
1472	Akodon montensis	78107669739	78107.67
1473	Martes pennanti	78106634934	78106.63

1474	Madoqua saltiana	77938203628	77938.20
1475	Craseomys regulus	77916315236	77916.32
1476	Heliosciurus ruwenzorii	77804026572	77804.03
1477	Myotis levis	77237509846	77237.51
1478	Marmosa robinsoni	76600535537	76600.54
1479	Eolagurus luteus	76319061507	76319.06
1480	Procolobus verus	76313733051	76313.73
1481	Priodontes maximus	76292081515	76292.08
1482	Sturnira oporaphilum	76233271976	76233.27
1483	Eumops delticus	76203343256	76203.34
1484	Monodelphis adusta	76086149532	76086.15
1485	Oligoryzomys destructor	76058681521	76058.68
1486	Aethalops alecto	75207649000	75207.65
1487	Sorex alpinus	75071391311	75071.39
1488	Rhinolophus sedulus	75064181160	75064.18
1489	Damaliscus lunatus	74875534758	74875.53
1490	Arielulus circumdatus	74715858534	74715.86
1491	Myosorex varius	74700238371	74700.24
1492	Mogera wogura	74602427112	74602.43
1493	Crossarchus obscurus	74590526335	74590.53
1494	Neoromicia brunnea	74572764794	74572.76
1495	Artibeus concolor	74542797785	74542.80
1496	Caenolestes fuliginosus	74373949488	74373.95
1497	Sturnira luisi	74314026450	74314.03
1498	Otolemur garnettii	74286551057	74286.55
1499	Aethomys ineptus	73951615065	73951.62
1500	Pipistrellus grandidieri	73909465306	73909.47
1501	Rhinolophus inops	73902365668	73902.37
1502	Murina annamitica	73886608509	73886.61
1503	Sorex veraecrucis	73656813469	73656.81
1504	Eospalax smithii	73580305210	73580.31
1505	Muntiacus crinifrons	73553845468	73553.85
1506	Dasyprocta prymnolopha	73525881332	73525.88
1507	Lycalopex gymnocercus	73461924971	73461.92
1508	Brachiones przewalskii	73430270846	73430.27
1509	Platyrrhinus nigellus	73352653445	73352.65
1510	Crossarchus alexandri	73269183389	73269.18
1511	Dipodomys ordii	73182274212	73182.27
1512	Meriones vinogradovi	72886278452	72886.28
1513	Jaculus blanfordi	72805647217	72805.65
1514	Rattus everetti	72796996906	72797.00

1515	Neodon clarkei	72398712809	72398.71
1516	Artibeus aequatorialis	72383131190	72383.13
1517	Mogera imaizumii	72331314982	72331.31
1518	Euryoryzomys russatus	72182533995	72182.53
1519	Paraechinus micropus	72120114207	72120.11
1520	Cercocebus torquatus	72081822052	72081.82
1521	Trachypithecus obscurus	72024172311	72024.17
1522	Herpestes vitticollis	71971137668	71971.14
1523	Myotis austroriparius	71877684221	71877.68
1524	Dermanura watsoni	71769779505	71769.78
1525	Peromyscus gratus	71707983113	71707.98
1526	Marmosa paraguayana	71272033793	71272.03
1527	Gerbilliscus guineae	71191893792	71191.89
1528	Nycteris major	70923663041	70923.66
1529	Myopterus whitleyi	70887800043	70887.80
1530	Lepus callotis	70743736611	70743.74
1531	Orthogeomys hispidus	70673370027	70673.37
1532	Galea spixii	70669823661	70669.82
1533	Kerivoula minuta	70491691803	70491.69
1534	Abrothrix olivaceus	70413140322	70413.14
1535	Chilomys instans	70317942763	70317.94
1536	Rhinolophus paradoxolophus	70288412842	70288.41
1537	Peromyscus melanophrys	70223991946	70223.99
1538	Anoura aequatoris	69993812922	69993.81
1539	Rhinolophus subrufus	69902794247	69902.79
1540	Capricornis crispus	69858227178	69858.23
1541	Mosia nigrescens	69717635711	69717.64
1542	Peromyscus melanotis	69425290011	69425.29
1543	Lepus townsendii	69410758729	69410.76
1544	Asellia patrizii	69396309840	69396.31
1545	Lycalopex griseus	69243642983	69243.64
1546	Cynictis penicillata	69240758053	69240.76
1547	Thaptomys nigrita	69200687219	69200.69
1548	Neotragus pygmaeus	69123528102	69123.53
1549	Trachypithecus crepusculus	68971795304	68971.80
1550	Apodemus ponticus	68864460167	68864.46
1551	Microtus mexicanus	68778232475	68778.23
1552	Eoglaucomys fimbriatus	68700090276	68700.09
1553	Monophyllus redmani	68687530740	68687.53
1554	Parahyaena brunnea	68635048718	68635.05
1555	Trachypithecus germaini	68575734250	68575.73

1556	Crocidura negligens	68532265953	68532.27
1557	Pteromys momonga	68457066647	68457.07
1558	Pteronotus parnellii	68398303689	68398.30
1559	Mormoops blainvillei	68398303689	68398.30
1560	Pteronotus quadridens	68398303689	68398.30
1561	Kannabateomys amblyonyx	68339010804	68339.01
1562	Paraxerus flavovittis	68186370280	68186.37
1563	Leopoldamys ciliatus	68181266395	68181.27
1564	Pantholops hodgsonii	68175204700	68175.20
1565	Hypsugo cadornae	68130447061	68130.45
1566	Neacomys tenuipes	68064582117	68064.58
1567	Natalus tumidirostris	67886603802	67886.60
1568	Talpa altaica	67475434303	67475.43
1569	Crocidura muricauda	67358255221	67358.26
1570	Peromyscus beatae	67188168602	67188.17
1571	Crocidura zarudnyi	67167744787	67167.74
1572	Tupaia glis	67138561506	67138.56
1573	Poliocitellus franklinii	67008972907	67008.97
1574	Nyctalus montanus	66948108302	66948.11
1575	Mazama nemorivaga	66760829219	66760.83
1576	Grammomys ibeanus	66447055299	66447.06
1577	Nycteris woodi	66373168086	66373.17
1578	Macaca fuscata	66363190903	66363.19
1579	Sorex mirabilis	66082014533	66082.01
1580	Phoniscus atrox	66012092400	66012.09
1581	Scarturus euphratica	65925080731	65925.08
1582	Cerradomys subflavus	65914907361	65914.91
1583	Alouatta guariba	65721435412	65721.44
1584	Handleyomys saturatior	65696298481	65696.30
1585	Euryzygomatomys spinosus	65597728238	65597.73
1586	Ptilocercus lowii	65583089735	65583.09
1587	Akodon cursor	65540196909	65540.20
1588	Microtus duodecimcostatus	65423701173	65423.70
1589	Sicista tianshanica	65367260960	65367.26
1590	Microtus longicaudus	65332015174	65332.02
1591	Caryomys eva	65128644251	65128.64
1592	Niviventer andersoni	65030334572	65030.33
1593	Sapajus nigritus	64907148350	64907.15
1594	Berylmys berdmorei	64827661349	64827.66
1595	Microtus ilaeus	64741019639	64741.02
1596	Aotus lemurinus	64514105002	64514.11

1597	Fukomys mechowii	64503647951	64503.65
1598	Chaetophractus villosus	64136074448	64136.07
1599	Heteromys australis	64046119886	64046.12
1600	Phyllotis xanthopygus	63888180817	63888.18
1601	Peromyscus polionotus	63840964595	63840.96
1602	Scutisorex somereni	63819880202	63819.88
1603	Perognathus flavus	63771637693	63771.64
1604	Gerbillus harwoodi	63768288821	63768.29
1605	Lepus yarkandensis	63559230668	63559.23
1606	Eudorcas rufifrons	63499290251	63499.29
1607	Hipposideros dyacorum	63448845046	63448.85
1608	Monodelphis domestica	63229199804	63229.20
1609	Pronolagus randensis	63050840819	63050.84
1610	Scotonycteris occidentalis	63049386193	63049.39
1611	Philander frenatus	63005088925	63005.09
1612	Crocidura obscurior	62965315396	62965.32
1613	Brachyphylla nana	62836419850	62836.42
1614	Sturnira magna	62626645348	62626.65
1615	Calomys musculinus	62514376868	62514.38
1616	Myotis bucharensis	62504228687	62504.23
1617	Zygodontomys brevicauda	62485351896	62485.35
1618	Glauconycteris humeralis	62323040071	62323.04
1619	Crocidura malayana	62169298341	62169.30
1620	Lophostoma occidentalis	62117028638	62117.03
1621	Coendou bicolor	62072167731	62072.17
1622	Rhipidomys latimanus	61827095790	61827.10
1623	Crocidura nanilla	61786006195	61786.01
1624	Baeodon alleni	61730051571	61730.05
1625	Platyrrhinus brachycephalus	61661555638	61661.56
1626	Suncus malayanus	61655921860	61655.92
1627	Pelea capreolus	61609530966	61609.53
1628	Sus barbatus	61435980961	61435.98
1629	Crocidura thalia	61166663713	61166.66
1630	Chaerephon aloysiisabaudiae	60982515760	60982.52
1631	Mystromys albicaudatus	60948483568	60948.48
1632	Scotinomys teguina	60654704278	60654.70
1633	Hypsugo ariel	60295003734	60295.00
1634	Dermanura glauca	60190081980	60190.08
1635	Plecotus sacrimontis	60174342714	60174.34
1636	Gracilinanus agilis	60027591365	60027.59
1637	Neotoma cinerea	60023517525	60023.52

1638	Heteromys anomalus	60018105259	60018.11
1639	Sorex asper	59956996116	59957.00
1640	Myotis moluccarum	59937398311	59937.40
1641	Rhipidomys mastacalis	59917773991	59917.77
1642	Tadarida lobata	59885603671	59885.60
1643	Euroscaptor klossi	59857054454	59857.05
1644	Talpa caeca	59806864203	59806.86
1645	Microtus savii	59706769187	59706.77
1646	Semnopithecus hector	59501668512	59501.67
1647	Gerbilliscus nigricaudus	59432592124	59432.59
1648	Peromyscus difficilis	59268428041	59268.43
1649	Aethomys nyikae	59216502528	59216.50
1650	Hipposideros fuliginosus	59210721404	59210.72
1651	Cercopithecus lowei	59153630756	59153.63
1652	Atelerix frontalis	59037459366	59037.46
1653	Naemorhedus goral	59030895946	59030.90
1654	Crocidura silacea	59007021979	59007.02
1655	Hipposideros madurae	58949703382	58949.70
1656	Pteropus lylei	58845264905	58845.26
1657	Neotoma magister	58775371093	58775.37
1658	Rhipidomys leucodactylus	58724383413	58724.38
1659	Gracilinanus marica	58607982937	58607.98
1660	Pelomys campanae	58542877648	58542.88
1661	Phyllonycteris poeyi	58486418049	58486.42
1662	Notiosorex crawfordi	58428427340	58428.43
1663	Phyllops falcatus	58372442146	58372.44
1664	Eptesicus bobrinskoi	58297077724	58297.08
1665	Chironax melanocephalus	58157742802	58157.74
1666	Cryptomys natalensis	58140082496	58140.08
1667	Bassaricyon neblina	58054031887	58054.03
1668	Beamys hindei	57793937614	57793.94
1669	Peromyscus boylii	57758836830	57758.84
1670	Sus celebensis	57717050802	57717.05
1671	Sylvilagus palustris	57672975612	57672.98
1672	Acomys muzei	57573281265	57573.28
1673	Dermanura rava	57498969737	57498.97
1674	Sorex excelsus	57377913699	57377.91
1675	Kerivoula whiteheadi	57346247424	57346.25
1676	Cloeotis percivali	56923228775	56923.23
1677	Crocidura flavescens	56899564783	56899.56
1678	Chaetophractus vellerosus	56763490146	56763.49

1679	Sorex volnuchini	56669449014	56669.45
1680	Peropteryx leucoptera	56395504683	56395.50
1681	Otomys helleri	56368208335	56368.21
1682	Gerbillus pyramidum	56250752034	56250.75
1683	Dasypus kappleri	56226228001	56226.23
1684	Arctocebus calabarensis	56150759753	56150.76
1685	Ochotona roylei	56094857944	56094.86
1686	Aethomys stannarius	56064190134	56064.19
1687	Ictidomys mexicanus	55931484793	55931.48
1688	Petaurus breviceps	55867492592	55867.49
1689	Myotis dinellii	55843083865	55843.08
1690	Monodelphis iheringi	55737991785	55737.99
1691	Oryx beisa	55723471199	55723.47
1692	Sorex satunini	55647940019	55647.94
1693	Miniopterus maghrebensis	55322530364	55322.53
1694	Artibeus fraterculus	55252162287	55252.16
1695	Poiana richardsonii	55212315765	55212.32
1696	Funambulus tristriatus	55179382341	55179.38
1697	Dologale dybowskii	55042907362	55042.91
1698	Euroscaptor grandis	54875130913	54875.13
1699	Lepus granatensis	54867138372	54867.14
1700	Pteropus griseus	54840088642	54840.09
1701	Harpyionycteris whiteheadi	54702368052	54702.37
1702	Rhinolophus euryotis	54665152661	54665.15
1703	Rhinolophus shameli	54601303775	54601.30
1704	Lonchophylla concava	54539200501	54539.20
1705	Gracilinanus microtarsus	54528685479	54528.69
1706	Myotis escalerai	54416592759	54416.59
1707	Onychomys leucogaster	54279340149	54279.34
1708	Platymops setiger	54211443508	54211.44
1709	Sorex roboratus	54105132009	54105.13
1710	Crocidura yankariensis	54017000280	54017.00
1711	Rhinolophus denti	53997402112	53997.40
1712	Callosciurus inornatus	53898285650	53898.29
1713	Bdeogale nigripes	53877909603	53877.91
1714	Aegialomys xanthaeolus	53793988639	53793.99
1715	Abrothrix longipilis	53787789409	53787.79
1716	Calomyscus mystax	53746494710	53746.49
1717	Talpa occidentalis	53728164476	53728.16
1718	Trinomys iheringi	53646879610	53646.88
1719	Murina rozendaali	53544631810	53544.63

1720	Heliosciurus punctatus	53493711477	53493.71
1721	Meriones rex	53406517945	53406.52
1722	Sauromys petrophilus	53184967330	53184.97
1723	Nycticebus javanicus	53150311731	53150.31
1724	Reithrodontomys montanus	53033948778	53033.95
1725	Cavia fulgida	52770833627	52770.83
1726	Dasypus hybridus	52769013346	52769.01
1727	Acomys louisae	52699188002	52699.19
1728	Chrotogale owstoni	52686478526	52686.48
1729	Zapus princeps	52599405910	52599.41
1730	Hystrix crassispinis	52467818204	52467.82
1731	Rheithrosciurus macrotis	52467818204	52467.82
1732	Crocidura foetida	52467680662	52467.68
1733	Eonycteris major	52467680662	52467.68
1734	Muntiacus atherodes	52467680662	52467.68
1735	Chiropodomys pusillus	52411392693	52411.39
1736	Crocidura maquassiensis	52089919679	52089.92
1737	Nycticebus menagensis	52014581901	52014.58
1738	Cryptotis tropicalis	51967523495	51967.52
1739	Uropsilus soricipes	51888761128	51888.76
1740	Dasyprocta azarae	51567670716	51567.67
1741	Oligoryzomys fornesi	51487527711	51487.53
1742	Dusicyon avus	51459254841	51459.25
1743	Sorex unguiculatus	51306235360	51306.24
1744	Acerodon jubatus	51188491710	51188.49
1745	Myotis gomantongensis	51111690171	51111.69
1746	Hoolock tianxing	51012966146	51012.97
1747	Mustela nigripes	50987683294	50987.68
1748	Eumops patagonicus	50982933521	50982.93
1749	Pronolagus crassicaudatus	50975699715	50975.70
1750	Histiotus laephotis	50972625022	50972.63
1751	Craseomys smithii	50955930527	50955.93
1752	Geomys bursarius	50869303677	50869.30
1753	Spermophilus relictus	50693303568	50693.30
1754	Exilisciurus exilis	50690606557	50690.61
1755	Dermanura bogotensis	50599258561	50599.26
1756	Helogale hirtula	50546555570	50546.56
1757	Histiotus humboldti	50302094183	50302.09
1758	Dremomys lokriah	50085478851	50085.48
1759	Dendrogale murina	49982191536	49982.19
1760	Steatomys krebsii	49900943147	49900.94

1761	Antidorcas marsupialis	49894549839	49894.55
1762	Neotamias minimus	49780455175	49780.46
1763	Pronolagus saundersiae	49774162250	49774.16
1764	Sorex monticola	49693631681	49693.63
1765	Theropithecus gelada	49664273186	49664.27
1766	Oecomys concolor	49423488731	49423.49
1767	Arvicanthis ansorgei	49287593767	49287.59
1768	Crocidura elgonius	49137522034	49137.52
1769	Cryptotis merriami	49099250449	49099.25
1770	Peromyscus levipes	49086326865	49086.33
1771	Rhinolophus subbadius	49071033397	49071.03
1772	Phoniscus jagorii	49030841277	49030.84
1773	Sigmodon leucotis	48811747530	48811.75
1774	Hemiechinus collaris	48733727775	48733.73
1775	Felis nigripes	48710205264	48710.21
1776	Scotophilus andrewreborii	48491541657	48491.54
1777	Crocidura lusitania	48408764233	48408.76
1778	Otomys denti	48073840704	48073.84
1779	Pteropus alecto	47953190519	47953.19
1780	Malacomys cansdalei	47927621772	47927.62
1781	Sylvilagus cunicularius	47778315717	47778.32
1782	Niviventer langbianis	47738959830	47738.96
1783	Nyctimene cephalotes	47719526307	47719.53
1784	Funisciurus carruthersi	47711518633	47711.52
1785	Histiotus macrotus	47633429695	47633.43
1786	Hylonycteris underwoodi	47622567034	47622.57
1787	Amblysomus hottentotus	47620552073	47620.55
1788	Hipposideros ridleyi	47417248119	47417.25
1789	Pronolagus rupestris	47270668453	47270.67
1790	Phyllomys medius	47265589912	47265.59
1791	Epixerus ebii	47240253989	47240.25
1792	Desmodilliscus braueri	47219887248	47219.89
1793	Nannospalax xanthodon	46936888675	46936.89
1794	Sorex antinorii	46670112461	46670.11
1795	Proechimys semispinosus	46404687091	46404.69
1796	Calomys laucha	46401878851	46401.88
1797	Acomys johannis	46400143633	46400.14
1798	Tadarida fulminans	46319237380	46319.24
1799	Ailurus fulgens	46249293807	46249.29
1800	Crocidura maurisca	46247249623	46247.25
1801	Damaliscus pygargus	46032866633	46032.87

1802	Myotis adversus	45985948120	45985.95
1803	Urocricetus kamensis	45976035113	45976.04
1804	Molossops neglectus	45960679235	45960.68
1805	Calomyscus bailwardi	45932323346	45932.32
1806	Mus mattheyi	45923099514	45923.10
1807	Gazella arabica	45823811231	45823.81
1808	Graphiurus platyops	45749083791	45749.08
1809	Hylaeamys megacephalus	45712852899	45712.85
1810	Lagostomus maximus	45452103361	45452.10
1811	Lonchophylla orienticollina	45376032709	45376.03
1812	Thomomys bottae	45322427468	45322.43
1813	Glossophaga morenoi	45313933986	45313.93
1814	Calomys callosus	45289710290	45289.71
1815	Lariscus niobe	45283320860	45283.32
1816	Peromyscus truei	45070141901	45070.14
1817	Chrysochloris stuhlmanni	45042837452	45042.84
1818	Megaloglossus azagnyi	45019927768	45019.93
1819	Niviventer fraternus	44972699485	44972.70
1820	Dipodomys ornatus	44936570354	44936.57
1821	Galea leucoblephara	44875082339	44875.08
1822	Plerotes anchietae	44701366856	44701.37
1823	Crocidura denti	44662597562	44662.60
1824	Mus bufo	44596550038	44596.55
1825	Mustela lutreolina	44439986110	44439.99
1826	Scarturus toussi	44413837377	44413.84
1827	Ochotona syrinx	44380961505	44380.96
1828	Akodon mollis	44325596955	44325.60
1829	Rangifer tarandus	44323571292	44323.57
1830	Necromys punctulatus	44042342082	44042.34
1831	Mops sarasinorum	43733520514	43733.52
1832	Phyllomys sulinus	43728963076	43728.96
1833	Herpestes ochraceus	43617246850	43617.25
1834	Sapajus apella	43513906319	43513.91
1835	Oligoryzomys longicaudatus	43511256144	43511.26
1836	Eumops bonariensis	43433957400	43433.96
1837	Platyrrhinus umbratus	43412485323	43412.49
1838	Peromyscus pectoralis	43332378609	43332.38
1839	Presbytis mitrata	43287243265	43287.24
1840	Thomomys talpoides	42822498636	42822.50
1841	Microtus chrotorrhinus	42687122974	42687.12
1842	Fukomys bocagei	42655129587	42655.13

1843	Naemorhedus caudatus	42616767667	42616.77
1844	Myotis fortidens	42544606107	42544.61
1845	Lasiurus varius	42535453979	42535.45
1846	Genetta cristata	42521115008	42521.12
1847	Calomys lepidus	42484723730	42484.72
1848	Myopus schisticolor	42419068576	42419.07
1849	Thrichomys laurentius	42339466898	42339.47
1850	Oxymycterus hispidus	42199528171	42199.53
1851	Eptesicus innoxius	42173962500	42173.96
1852	Galago matschiei	41999149594	41999.15
1853	Myotis chiloensis	41899992444	41899.99
1854	Sigmodontomys alfari	41813431034	41813.43
1855	Akodon azarae	41732343196	41732.34
1856	Blarinomys breviceps	41607693523	41607.69
1857	Marmosops impavidus	41537296673	41537.30
1858	Bdeogale jacksoni	41505585266	41505.59
1859	Otomys typus	41491461181	41491.46
1860	Crocidura hutanis	41490918692	41490.92
1861	Thylamys pallidior	41480295687	41480.30
1862	Erophylla bombifrons	41449291214	41449.29
1863	Lasiurus minor	41449291214	41449.29
1864	Vampyressa melissa	41403293917	41403.29
1865	Blarina hylophaga	41288226387	41288.23
1866	Orthogeomys grandis	41252693064	41252.69
1867	Myotis sicarius	41227304285	41227.30
1868	Niviventer tenaster	41153132801	41153.13
1869	Rattus annandalei	41113713202	41113.71
1870	Rhagomys rufescens	41035686326	41035.69
1871	Cercocebus lunulatus	40980449107	40980.45
1872	Kerivoula smithii	40942283577	40942.28
1873	Gerbillus simoni	40887682614	40887.68
1874	Callithrix jacchus	40870525993	40870.53
1875	Paradoxurus jerdoni	40668999369	40669.00
1876	Balionycteris seimundi	40546526258	40546.53
1877	Coendou pruinosus	40472615230	40472.62
1878	Aotus griseimembra	40186756723	40186.76
1879	Cryptotis alticola	40132375948	40132.38
1880	Genetta victoriae	40130122992	40130.12
1881	Nasalis larvatus	40124038511	40124.04
1882	Sciurus stramineus	40106859118	40106.86
1883	Lenothrix canus	40094182787	40094.18

1884	Calomyscus elburzensis	40003922255	40003.92
1885	Arvicanthis nairobae	39974441583	39974.44
1886	Sapajus libidinosus	39843437396	39843.44
1887	Reithrodontomys microdon	39452384652	39452.38
1888	Blarinella wardi	39445902809	39445.90
1889	Sciurus ignitus	39432234013	39432.23
1890	Monodelphis scalops	39429960924	39429.96
1891	Sicista strandi	39321783721	39321.78
1892	Dobsonia crenulata	39277344616	39277.34
1893	Aotus azarae	39156751558	39156.75
1894	Callospermophilus lateralis	39084717379	39084.72
1895	Petinomys fuscocapillus	39080714987	39080.71
1896	Presbytis siamensis	39058641563	39058.64
1897	Eonycteris robusta	38896920808	38896.92
1898	Crocidura paradoxura	38887064749	38887.06
1899	Oxymycterus quaestor	38839500717	38839.50
1900	Sciurocheirus alleni	38759323620	38759.32
1901	Hydromys chrysogaster	38737800171	38737.80
1902	Spalax microphthalmus	38695796579	38695.80
1903	Callicebus nigrifrons	38686529683	38686.53
1904	Tupaia splendidula	38650548012	38650.55
1905	Ailurops ursinus	38636789598	38636.79
1906	Sorex samniticus	38635963042	38635.96
1907	Thomomys umbrinus	38603131223	38603.13
1908	Abrothrix jelskii	38541062081	38541.06
1909	Dobsonia exoleta	38516898908	38516.90
1910	Kerivoula lenis	38494387764	38494.39
1911	Akodon paranaensis	38473833444	38473.83
1912	Microtus gerbii	38448440222	38448.44
1913	Sylvisorex ollula	38410210611	38410.21
1914	Moschus leucogaster	38286937821	38286.94
1915	Presbytis melalophos	38257108123	38257.11
1916	Rhinolophus shortridgei	38217394938	38217.39
1917	Casinycteris argynnis	38164911549	38164.91
1918	Suncus infinitesimus	38147332235	38147.33
1919	Chiromyscus chiropus	38142389417	38142.39
1920	Euoticus pallidus	38132923303	38132.92
1921	Microtus lusitanicus	38119679752	38119.68
1922	Promops davisoni	38116807774	38116.81
1923	Epomophorus angolensis	37972675062	37972.68
1924	Cryptonanus agricolai	37935638123	37935.64

1925	Strigocuscus celebensis	37874024877	37874.02
1926	Cryptotis orophila	37841649946	37841.65
1927	Reithrodon auritus	37806982016	37806.98
1928	Cynocephalus volans	37794238350	37794.24
1929	Sundasciurus philippinensis	37794200016	37794.20
1930	Exilisciurus concinnus	37794200016	37794.20
1931	Carlito syrichta	37793253706	37793.25
1932	Crocidura theresae	37755436580	37755.44
1933	Grammomys cometes	37736168705	37736.17
1934	Lama guanicoe	37697604219	37697.60
1935	Acerodon celebensis	37678277513	37678.28
1936	Rousettus celebensis	37605457721	37605.46
1937	Ptenochirus minor	37588602266	37588.60
1938	Clyomys laticeps	37569109132	37569.11
1939	Bullimus bagobus	37545146126	37545.15
1940	Hipposideros pelingensis	37536936840	37536.94
1941	Otospermophilus beecheyi	37508556696	37508.56
1942	Hylobates moloch	37444264177	37444.26
1943	Alouatta caraya	37430911797	37430.91
1944	Thoopterus nigrescens	37404586724	37404.59
1945	Neacomys spinosus	37393476463	37393.48
1946	Dermanura anderseni	37392953280	37392.95
1947	Kerodon rupestris	37384520926	37384.52
1948	Marmosa regina	37280333022	37280.33
1949	Herpestes pulverulentus	37272195683	37272.20
1950	Gerbillus mesopotamiae	37252781861	37252.78
1951	Spermophilus suslicus	37249482318	37249.48
1952	Bassaricyon alleni	37192872693	37192.87
1953	Niviventer excelsior	37189007063	37189.01
1954	Marmota marmota	37116197749	37116.20
1955	Styloctenium wallacei	37035717286	37035.72
1956	Lophuromys machangui	36940862635	36940.86
1957	Vulpes macrotis	36884214339	36884.21
1958	Suricata suricatta	36839272798	36839.27
1959	Rhinolophus tatar	36773369232	36773.37
1960	Thoopterus suhaniahae	36762908921	36762.91
1961	Harpyionycteris celebensis	36761793551	36761.79
1962	Myotis weberi	36759453309	36759.45
1963	Bunomys chrysocomus	36758818747	36758.82
1964	Maxomys musschenbroekii	36758818747	36758.82
1965	Paruromys dominator	36758818747	36758.82

1966	Rubrisciurus rubriventer	36758818747	36758.82
1967	Scotophilus celebensis	36758559463	36758.56
1968	Muntiacus montanus	36697952348	36697.95
1969	Maxomys inflatus	36696616510	36696.62
1970	Loxodontomys micropus	36686204822	36686.20
1971	Scotonycteris zenkeri	36680280854	36680.28
1972	Juliomys pictipes	36633709262	36633.71
1973	Moschus fuscus	36620359526	36620.36
1974	Myomimus setzeri	36561527578	36561.53
1975	Anomalurus pusillus	36548667231	36548.67
1976	Fukomys ochraceocinereus	36444458599	36444.46
1977	Microtus bucharensis	36299409950	36299.41
1978	Cebus imitator	36281361605	36281.36
1979	Rattus hoffmanni	36251101284	36251.10
1980	Desmodillus auricularis	36221828586	36221.83
1981	Hippocamelus antisensis	36128366980	36128.37
1982	Neotoma leucodon	36062795793	36062.80
1983	Ateles fusciceps	36047601937	36047.60
1984	Hylaeamys yunganus	36021593538	36021.59
1985	Sciurus oculatus	35966348226	35966.35
1986	Muntiacus truongsonensis	35963477703	35963.48
1987	Lyncodon patagonicus	35961305751	35961.31
1988	Sorex dispar	35885048593	35885.05
1989	Crocidura tanakae	35878799871	35878.80
1990	Amorphochilus schnablii	35820094188	35820.09
1991	Tupaia gracilis	35804979805	35804.98
1992	Marmosops incanus	35712754356	35712.75
1993	Cercopithecus denti	35673268442	35673.27
1994	Cercopithecus erythrogaster	35598249915	35598.25
1995	Sorex raddei	35515835555	35515.84
1996	Sigmodon toltecus	35435874914	35435.87
1997	Leopardus guigna	35401696967	35401.70
1998	Rhinopoma muscatellum	35383813685	35383.81
1999	Mormopterus kalinowskii	35340427826	35340.43
2000	Wilfredomys oenax	35312002329	35312.00
2001	Presbytis robinsoni	35281921960	35281.92
2002	Syconycteris australis	35241255132	35241.26
2003	Spermophilus xanthoprymnus	35230614951	35230.61
2004	Natalus major	35207372140	35207.37
2005	Gerbillus gleadowi	35110955632	35110.96
2006	Calomys tener	34979783172	34979.78

2007	Ochotona opaca	34961396215	34961.40
2008	Laephotis botswanae	34920926994	34920.93
2009	Makalata didelphoides	34892919298	34892.92
2010	Crocidura grayi	34722600971	34722.60
2011	Abrocoma cinerea	34692667367	34692.67
2012	Oxymycterus angularis	34650215206	34650.22
2013	Proechimys guairae	34649706563	34649.71
2014	Maxomys hellwaldii	34633200998	34633.20
2015	Bassaricyon medius	34620142597	34620.14
2016	Platalina genovensium	34618224088	34618.22
2017	Sorex saussurei	34613665256	34613.67
2018	Lophuromys chrysopus	34608781008	34608.78
2019	Cebus capucinus	34539973706	34539.97
2020	Rhogeessa parvula	34529956469	34529.96
2021	Piliocolobus badius	34512604486	34512.60
2022	Mazama bororo	34490625091	34490.63
2023	Phyllomys lamarum	34390957622	34390.96
2024	Apodemus epimelas	34385958890	34385.96
2025	Rhinolophus yunanensis	34338800265	34338.80
2026	Saiga tatarica	34279504039	34279.50
2027	Hylomyscus vulcanorum	34229628193	34229.63
2028	Sturnira aratathomasi	34143453761	34143.45
2029	Myotis longipes	34049451343	34049.45
2030	Meriones zarudnyi	33960706630	33960.71
2031	Otopteropus cartilagonodus	33808134249	33808.13
2032	Microcavia australis	33783723320	33783.72
2033	Presbytis comata	33721859050	33721.86
2034	Geomys pinetis	33675031372	33675.03
2035	Thomasomys baeops	33540515090	33540.52
2036	Thomasomys taczanowskii	33527132861	33527.13
2037	Crocidura beccarii	33503465277	33503.47
2038	Graphiurus christyi	33474630320	33474.63
2039	Scotophilus ejetai	33464203096	33464.20
2040	Bauerus dubiaquercus	33340411491	33340.41
2041	Eptesicus chiriquinus	33089345971	33089.35
2042	Microryzomys altissimus	33067752104	33067.75
2043	Histiotus alienus	33020499155	33020.50
2044	Nyctinomops femorosaccus	32889153355	32889.15
2045	Sorex sinalis	32830400344	32830.40
2046	Pudu mephistophiles	32786821211	32786.82
2047	Tupaia everetti	32684707857	32684.71

2048	Apomys insignis	32684039240	32684.04
2049	Sundasciurus mindanensis	32672775538	32672.78
2050	Pectinator spekei	32672147937	32672.15
2051	Lophuromys woosnami	32664587194	32664.59
2052	Sorex merriami	32631902239	32631.90
2053	Thrichomys apereoides	32613047691	32613.05
2054	Microtus multiplex	32580851211	32580.85
2055	Palaeopropithecus ingens	32523416772	32523.42
2056	Graomys chacoensis	32462041029	32462.04
2057	Setifer setosus	32460882766	32460.88
2058	Tenrec ecaudatus	32460882596	32460.88
2059	Fukomys darlingi	32454870341	32454.87
2060	Lenomys meyeri	32382507718	32382.51
2061	Lynx canadensis	32323375691	32323.38
2062	Zaedyus pichiy	32213189759	32213.19
2063	Leopardus jacobita	32212535289	32212.54
2064	Microtus montanus	32175044797	32175.04
2065	Myotis occultus	32114434668	32114.43
2066	Marmota flaviventris	31945861166	31945.86
2067	Paragalago granti	31865590231	31865.59
2068	Gerbillurus paeba	31765198393	31765.20
2069	Cebus versicolor	31637217904	31637.22
2070	Lasiurus salinae	31636557741	31636.56
2071	Euryoryzomys macconnelli	31623371393	31623.37
2072	Martes americana	31596592144	31596.59
2073	Platyrrhinus matapalensis	31594268545	31594.27
2074	Millardia gleadowi	31344864052	31344.86
2075	Megaerops wetmorei	31289554722	31289.55
2076	Echymipera kalubu	31165961136	31165.96
2077	Dobsonia magna	31160982721	31160.98
2078	Akodon boliviensis	31065217156	31065.22
2079	Oxymycterus nasutus	31057604769	31057.60
2080	Sorex arcticus	31056132509	31056.13
2081	Presbytis sumatranus	31042735185	31042.74
2082	Octodontomys gliroides	31035460527	31035.46
2083	Spilocuscus maculatus	31005086362	31005.09
2084	Acomys kempi	30966382856	30966.38
2085	Sylvilagus nuttallii	30918202618	30918.20
2086	Myotis schaubi	30914010950	30914.01
2087	Trachypithecus popa	30752029198	30752.03
2088	Bibimys labiosus	30682964586	30682.96

2089	Zygodontomys brunneus	30619214757	30619.21
2090	Cynopterus nusatenggara	30599287626	30599.29
2091	Hipposideros tephrus	30597197371	30597.20
2092	Uromys caudimaculatus	30590765641	30590.77
2093	Sorex vagrans	30500941852	30500.94
2094	Lepus starcki	30480238839	30480.24
2095	Genetta tigrina	30456032140	30456.03
2096	Pseudoryzomys simplex	30418146112	30418.15
2097	Rhinophylla alethina	30406886412	30406.89
2098	Wiedomys pyrrhorhinos	30373091418	30373.09
2099	Dobsonia peronii	30359608756	30359.61
2100	Tachyglossus aculeatus	30322675565	30322.68
2101	Thylamys elegans	30302396681	30302.40
2102	Echymipera rufescens	30191783539	30191.78
2103	Heteromys pictus	30028533923	30028.53
2104	Chaerephon russatus	30021349136	30021.35
2105	Crocidura jouvenetae	29963917967	29963.92
2106	Cercopithecus roloway	29945342334	29945.34
2107	Lophostoma carrikeri	29875217430	29875.22
2108	Platyrrhinus incarum	29827872297	29827.87
2109	Phyllomys dasythrix	29822333199	29822.33
2110	Rhipidomys caucensis	29724935720	29724.94
2111	Microtus californicus	29715970553	29715.97
2112	Sorex gracillimus	29711042393	29711.04
2113	Vicugna vicugna	29645586067	29645.59
2114	Phalanger gymnotis	29616769813	29616.77
2115	Rhinolophus thailandensis	29613265940	29613.27
2116	Catopuma temminckii	29599156943	29599.16
2117	Atlantoxerus getulus	29596958046	29596.96
2118	Cynomops mexicanus	29585183467	29585.18
2119	Transandinomys bolivaris	29453753961	29453.75
2120	Bunomys andrewsi	29427215359	29427.22
2121	Hypsugo macrotis	29407021751	29407.02
2122	Crocidura monax	29341801543	29341.80
2123	Xenuromys barbatus	29304633616	29304.63
2124	Cercopithecus sclateri	29201162296	29201.16
2125	Chalinolobus gouldii	29181508550	29181.51
2126	Oenomys ornatus	29163200830	29163.20
2127	Murexia longicaudata	29150803621	29150.80
2128	Thomasomys contradictus	29135228080	29135.23
2129	Pteronotus rubiginosus	29090242602	29090.24

2130	Mus indutus	28980867627	28980.87
2131	Dasyurus albopunctatus	28959821618	28959.82
2132	Balantiopteryx io	28950096160	28950.10
2133	Xerospermophilus spilosoma	28934583106	28934.58
2134	Connochaetes gnou	28873117003	28873.12
2135	Cynomys ludovicianus	28865490567	28865.49
2136	Lemniscomys bellieri	28776222577	28776.22
2137	Berylmys mackenziei	28725878708	28725.88
2138	Chalinolobus morio	28721500320	28721.50
2139	Graomys griseoflavus	28691154246	28691.15
2140	Thomasomys laniger	28690300033	28690.30
2141	Crocidura buettikoferi	28596679032	28596.68
2142	Trichosurus vulpecula	28596541773	28596.54
2143	Distoechurus pennatus	28580721939	28580.72
2144	Nyctophilus geoffroyi	28572971116	28572.97
2145	Kerivoula cuprosa	28327701313	28327.70
2146	Crocidura attila	28271110286	28271.11
2147	Sylvilagus bachmani	28263455805	28263.46
2148	Perognathus flavescens	28261087412	28261.09
2149	Trinomys setosus	28237415021	28237.42
2150	Galea musteloides	28231947629	28231.95
2151	Eudorcas thomsonii	28206616005	28206.62
2152	Clethrionomys centralis	28149137653	28149.14
2153	Microtus daghestanicus	28059133683	28059.13
2154	Apodemus rusiges	27982372138	27982.37
2155	Tlacuatzin canescens	27951978274	27951.98
2156	Sorex trowbridgii	27892437922	27892.44
2157	Genetta poensis	27859240217	27859.24
2158	Gerbillus dunni	27839915081	27839.92
2159	Euderma maculatum	27816848892	27816.85
2160	Hylomyscus stella	27749172335	27749.17
2161	Balionycteris maculata	27738559039	27738.56
2162	Mustela subpalmata	27691525022	27691.53
2163	Paraxerus alexandri	27670659975	27670.66
2164	Elephantulus intufi	27585611245	27585.61
2165	Calomys expulsus	27517258615	27517.26
2166	Monodelphis kunsi	27514849032	27514.85
2167	Oxymycterus paramensis	27468298464	27468.30
2168	Ozimops planiceps	27433829630	27433.83
2169	Miniopterus macrocneme	27408998281	27409.00
2170	Erophylla sezekorni	27339820659	27339.82

2171	Pteronotus macleayii	27339820659	27339.82
2172	Aeromys thomasi	27332636184	27332.64
2173	Hipposideros pygmaeus	27312329412	27312.33
2174	Kerivoula dongduongana	27288551018	27288.55
2175	Sciurus igniventris	27287732333	27287.73
2176	Rhipidomys venezuelae	27242254258	27242.25
2177	Phyllotis darwini	27225115725	27225.12
2178	Eidolon dupreanum	27210618348	27210.62
2179	Alouatta pigra	27194140138	27194.14
2180	Mazama nana	27137340514	27137.34
2181	Lophostoma evotis	27087919771	27087.92
2182	Austronomus australis	27048360555	27048.36
2183	Xerus inauris	27046627712	27046.63
2184	Vampyriscus nymphaea	27045253265	27045.25
2185	Miopithecus talapoin	27026903840	27026.90
2186	Hoplomys gymnurus	27014098519	27014.10
2187	Allactodipus bobrinskii	26983709456	26983.71
2188	Funisciurus substriatus	26916400195	26916.40
2189	Tolypeutes tricinctus	26813174708	26813.17
2190	Cryptonanus chacoensis	26761371613	26761.37
2191	Auliscomys pictus	26716921351	26716.92
2192	Phalanger orientalis	26679653318	26679.65
2193	Lonchophylla hesperia	26659307554	26659.31
2194	Galago gallarum	26642212268	26642.21
2195	Cephalophus ogilbyi	26617991453	26617.99
2196	Phyllotis osilae	26614987347	26614.99
2197	Lemniscomys griselda	26504705772	26504.71
2198	Crocidura macarthuri	26486876039	26486.88
2199	Tamiasciurus douglasii	26412310510	26412.31
2200	Conepatus humboldtii	26353750690	26353.75
2201	Marmosa alstoni	26315589155	26315.59
2202	Loxodonta cyclotis	26252080119	26252.08
2203	Artibeus inopinatus	26240552949	26240.55
2204	Peroryctes raffrayana	26204153886	26204.15
2205	Cebus aequatorialis	26166859891	26166.86
2206	Eothenomys custos	26166214421	26166.21
2207	Anomalurus pelii	26142384083	26142.38
2208	Myospalax myospalax	26120420134	26120.42
2209	Antilocapra americana	26107559927	26107.56
2210	Sciurus griseus	25975102713	25975.10
2211	Sorex ornatus	25948527957	25948.53

2212	Cryptotis goodwini	25904389765	25904.39
2213	Idiurus zenkeri	25894568759	25894.57
2214	Murexia melanurus	25867530398	25867.53
2215	Delomys dorsalis	25839099359	25839.10
2216	Platyrrhinus albericoi	25784344529	25784.34
2217	Oligoryzomys andinus	25773733003	25773.73
2218	Gerbillus tarabuli	25762975132	25762.98
2219	Pseudocheirus peregrinus	25755519332	25755.52
2220	Myotis goudoti	25751106277	25751.11
2221	Marmosops fuscatus	25718976975	25718.98
2222	Acerodon mackloti	25711662634	25711.66
2223	Saccolaimus flaviventris	25676965462	25676.97
2224	Vespadelus regulus	25665806407	25665.81
2225	Peromyscus attwateri	25660316526	25660.32
2226	Crocidura eburnea	25653306839	25653.31
2227	Berylmys manipulus	25646012178	25646.01
2228	Miniopterus manavi	25612063259	25612.06
2229	Bassaricyon gabbii	25610314339	25610.31
2230	Alouatta belzebul	25593028404	25593.03
2231	Ochotona rutila	25585262262	25585.26
2232	Pithecheir parvus	25578907934	25578.91
2233	Drymoreomys albimaculatus	25543886564	25543.89
2234	Cercartetus caudatus	25521951153	25521.95
2235	Elephantulus fuscipes	25492872566	25492.87
2236	Talpa davidiana	25453976562	25453.98
2237	Genetta johnstoni	25432660403	25432.66
2238	Otomys sloggetti	25432171847	25432.17
2239	Sorex nanus	25401073621	25401.07
2240	Anoura luismanueli	25370653303	25370.65
2241	Episoriculus leucops	25367588775	25367.59
2242	Thomasomys cinereiventer	25364830099	25364.83
2243	Gerbillus andersoni	25362804684	25362.80
2244	Phacochoerus aethiopicus	25332266719	25332.27
2245	Mustela africana	25329058981	25329.06
2246	Acomys selousi	25325708423	25325.71
2247	Auliscomys boliviensis	25286039027	25286.04
2248	Tupaia dorsalis	25225354884	25225.35
2249	Scapanus latimanus	25072826237	25072.83
2250	Hyperacrius fertilis	25043950178	25043.95
2251	Lycalopex vetulus	25041046753	25041.05
2252	Choloepus didactylus	24981426706	24981.43

2253	Chelemys macronyx	24971635565	24971.64
2254	Hipposideros grandis	24964810391	24964.81
2255	Alticola macrotis	24937276778	24937.28
2256	Crocidura rhoditis	24923518608	24923.52
2257	Viverra civettina	24896589355	24896.59
2258	Paramelomys mollis	24886964173	24886.96
2259	Crocidura grandiceps	24850050441	24850.05
2260	Paramelomys rubex	24831412534	24831.41
2261	Scotophilus robustus	24810973281	24810.97
2262	Acomys percivali	24756168988	24756.17
2263	Mops midas	24660399367	24660.40
2264	Carollia benkeithi	24652102657	24652.10
2265	Myotis annectans	24597324303	24597.32
2266	Microsciurus mimulus	24568879760	24568.88
2267	Pelomys hopkinsi	24554318520	24554.32
2268	Eremodipus lichtensteini	24540288939	24540.29
2269	Rattus steini	24539623320	24539.62
2270	Handleyomys fuscatus	24515979788	24515.98
2271	Crocidura floweri	24513135522	24513.14
2272	Mallomys rothschildi	24502889177	24502.89
2273	Microperoryctes longicauda	24421144015	24421.14
2274	Hipposideros lekaguli	24387311376	24387.31
2275	Uromys anak	24302025724	24302.03
2276	Hylomyscus arcimontensis	24294854187	24294.85
2277	Murexia naso	24281033694	24281.03
2278	Microtus liechtensteini	24274962632	24274.96
2279	Dactylonax palpator	24268657321	24268.66
2280	Myomyscus yemeni	24229730725	24229.73
2281	Pseudochirops cupreus	24120726322	24120.73
2282	Niviventer eha	24093338422	24093.34
2283	Pteropus lombocensis	24070328862	24070.33
2284	Microsciurus alfari	24042807809	24042.81
2285	Eligmodontia typus	24040197307	24040.20
2286	Anisomys imitator	24002629335	24002.63
2287	Thylamys venustus	23903022016	23903.02
2288	Taterillus pygargus	23872272054	23872.27
2289	Sciurus spadiceus	23866120178	23866.12
2290	Mustela felipei	23864522415	23864.52
2291	Necromys urichi	23827127288	23827.13
2292	Dasymys foxi	23807350018	23807.35
2293	Alouatta arctoidea	23782186049	23782.19

2294	Sigmodon fulviventer	23730827518	23730.83
2295	Proechimys roberti	23695565898	23695.57
2296	Colobus polykomos	23643966915	23643.97
2297	Dipodomys phillipsii	23641723700	23641.72
2298	Oxymycterus dasytrichus	23605463498	23605.46
2299	Aethomys thomasi	23575288752	23575.29
2300	Macropus giganteus	23552247676	23552.25
2301	Callithrix aurita	23549807340	23549.81
2302	Hipposideros khasiana	23506781350	23506.78
2303	Cercopithecus pogonias	23445240620	23445.24
2304	Auliscomys sublimis	23440400678	23440.40
2305	Dipodomys merriami	23424350536	23424.35
2306	Nyctophilus gouldi	23386374761	23386.37
2307	Myomyscus angolensis	23358704007	23358.70
2308	Wallabia bicolor	23296560995	23296.56
2309	Nyctiellus lepidus	23279045908	23279.05
2310	Chilonatalus macer	23278350411	23278.35
2311	Capromys pilorides	23278220586	23278.22
2312	Lemmiscus curtatus	23198736562	23198.74
2313	Heliosciurus undulatus	23194070504	23194.07
2314	Herpestes fuscus	23124663496	23124.66
2315	Boneia bidens	23100806700	23100.81
2316	Andinomys edax	23023975910	23023.98
2317	Soriculus nigrescens	22976149939	22976.15
2318	Aethomys bocagei	22959177428	22959.18
2319	Lasiurus insularis	22888237724	22888.24
2320	Mysateles prehensilis	22887659337	22887.66
2321	Presbytis rubicunda	22848923564	22848.92
2322	Geoxus valdivianus	22842463400	22842.46
2323	Abrothrix andinus	22800054854	22800.05
2324	Sorex orizabae	22794249238	22794.25
2325	Rhinolophus philippinensis	22761361367	22761.36
2326	Peromyscus gymnotis	22713553012	22713.55
2327	Pipistrellus collinus	22713133973	22713.13
2328	Proechimys decumanus	22679995132	22680.00
2329	Neomicroxus bogotensis	22637514062	22637.51
2330	Pseudochirops corinnae	22587328807	22587.33
2331	Pteropus poliocephalus	22551501034	22551.50
2332	Geomys breviceps	22543222463	22543.22
2333	Oryzomys gorgasi	22542671554	22542.67
2334	Myotis macropus	22538317451	22538.32

2335	Akodon lutescens	22530060723	22530.06
2336	Nyctimene certans	22417955170	22417.96
2337	Trachypithecus margarita	22369617625	22369.62
2338	Apodemus hyrcanicus	22334076774	22334.08
2339	Sigmodon mascotensis	22297221609	22297.22
2340	Rusa marianna	22228410293	22228.41
2341	Crocidura cinderella	22222293118	22222.29
2342	Acrobates pygmaeus	22207380125	22207.38
2343	Zaglossus bartoni	22199301869	22199.30
2344	Murina tubinaris	22154173498	22154.17
2345	Spermophilus major	22143856439	22143.86
2346	Sorex veraepacis	22127630144	22127.63
2347	Neotragus batesi	22087263804	22087.26
2348	Cercopithecus campbelli	22079245719	22079.25
2349	Myotis ciliolabrum	22055895615	22055.90
2350	Miniopterus gleni	22033158046	22033.16
2351	Myosorex cafer	22012409644	22012.41
2352	Rhinolophus deckenii	22009817166	22009.82
2353	Neurotrichus gibbsii	22005370496	22005.37
2354	Taterillus congicus	21947333117	21947.33
2355	Muriculus imberbis	21893276516	21893.28
2356	Dendromus lovati	21885050104	21885.05
2357	Cercocebus atys	21853214035	21853.21
2358	Phascolosorex dorsalis	21771145222	21771.15
2359	Phascolarctos cinereus	21741763664	21741.76
2360	Phalanger sericeus	21732723021	21732.72
2361	Chaerephon johorensis	21716919361	21716.92
2362	Scapanus orarius	21638643857	21638.64
2363	Equus hemionus	21618447396	21618.45
2364	Gerbillus maghrebi	21571561401	21571.56
2365	Hodomys alleni	21492692081	21492.69
2366	Mastomys kollmannspergeri	21484309396	21484.31
2367	Callithrix penicillata	21466354100	21466.35
2368	Pygathrix nigripes	21428930476	21428.93
2369	Pogonomys macrourus	21413603445	21413.60
2370	Rhipidomys macrurus	21349722814	21349.72
2371	Austronomus kuboriensis	21299192164	21299.19
2372	Calomys sorellus	21287034977	21287.03
2373	Akodon varius	21259549248	21259.55
2374	Phyllomys nigrispinus	21160185716	21160.19
2375	Akodon serrensis	21158551561	21158.55

2376	Nycticebus hilleri	21157647226	21157.65
2377	Myotis pruinosus	21157476726	21157.48
2378	Phalanger carmelitae	21114831104	21114.83
2379	Dorcopsulus vanheurni	21065361191	21065.36
2380	Mastomys huberti	21006440599	21006.44
2381	Dactylopsila trivirgata	20997158466	20997.16
2382	Lasiurus pfeifferi	20966225745	20966.23
2383	Dasyprocta mexicana	20964283955	20964.28
2384	Callosciurus phayrei	20950075235	20950.08
2385	Monodelphis peruviana	20921945691	20921.95
2386	Vespadelus darlingtoni	20831034307	20831.03
2387	Marmosops noctivagus	20822862135	20822.86
2388	Acomys ngurui	20808669916	20808.67
2389	Eliomys melanurus	20787931961	20787.93
2390	Talpa romana	20734731775	20734.73
2391	Melomys rufescens	20730321164	20730.32
2392	Oligoryzomys stramineus	20713511048	20713.51
2393	Rhipidomys itoan	20688690591	20688.69
2394	Monodelphis palliolata	20675114676	20675.11
2395	Eligmodontia puerulus	20625207680	20625.21
2396	Rupicapra rupicapra	20559024968	20559.02
2397	Myomimus personatus	20558607706	20558.61
2398	Lepus corsicanus	20513010527	20513.01
2399	Thallomys nigricauda	20506873414	20506.87
2400	Heteromys teleus	20464286691	20464.29
2401	Microtus majori	20454358744	20454.36
2402	Vespadelus vulturnus	20430965394	20430.97
2403	Saimiri cassiquiarensis	20409392380	20409.39
2404	Crocidura smithii	20347243187	20347.24
2405	Murexia habbema	20255771652	20255.77
2406	Gerbillus nancillus	20221749850	20221.75
2407	Rhinolophus megaphyllus	20182013664	20182.01
2408	Mesomys hispidus	20147142743	20147.14
2409	Rhinolophus creaghi	20128290825	20128.29
2410	Rhipidomys couesi	20120522199	20120.52
2411	Onychomys torridus	20104901683	20104.90
2412	Kerivoula flora	20050385250	20050.39
2413	Suncus hututsi	19993785720	19993.79
2414	Cryptoprocta ferox	19888574785	19888.57
2415	Synaptomys borealis	19884622406	19884.62
2416	Sorex bendirii	19867887941	19867.89

2417	Myosorex babaulti	19863081041	19863.08
2418	Gerbillus famulus	19823821159	19823.82
2419	Sicista severtzovi	19806315263	19806.32
2420	Falsistrellus tasmaniensis	19799395501	19799.40
2421	Mormopterus jugularis	19771159983	19771.16
2422	Rhipidomys fulviventer	19741865045	19741.87
2423	Gerbillus latastei	19649713639	19649.71
2424	Ateles hybridus	19609573126	19609.57
2425	Perognathus longimembris	19530560269	19530.56
2426	Aplodontia rufa	19515081923	19515.08
2427	Grammomys buntingi	19512149700	19512.15
2428	Mus baoulei	19485344447	19485.34
2429	Akodon albiventer	19461261207	19461.26
2430	Mus shortridgei	19414297938	19414.30
2431	Rousettus madagascariensis	19390110071	19390.11
2432	Mops congicus	19359488095	19359.49
2433	Dasyprocta fuliginosa	19350178714	19350.18
2434	Marmosa constantiae	19349011418	19349.01
2435	Myotis atacamensis	19347032625	19347.03
2436	Dremomys gularis	19287328960	19287.33
2437	Microtus oregoni	19285354072	19285.35
2438	Crocidura elongata	19259332282	19259.33
2439	Elephantulus fuscus	19255315286	19255.32
2440	Hylomyscus pamfi	19248989403	19248.99
2441	Thomasomys nicefori	19248167016	19248.17
2442	Phyllomys pattoni	19169262764	19169.26
2443	Peromyscus stirtoni	19115076024	19115.08
2444	Neotoma bryanti	19044069121	19044.07
2445	Saimiri collinsi	19034392139	19034.39
2446	Neodon sikimensis	19015428909	19015.43
2447	Thylogale browni	19010920798	19010.92
2448	Hyperacrius wynnei	18973081594	18973.08
2449	Microtus townsendii	18944203794	18944.20
2450	Gerbillus poecilops	18913447686	18913.45
2451	Cryptotis squamipes	18893034341	18893.03
2452	Chlorotalpa sclateri	18849031422	18849.03
2453	Praomys verschureni	18827622123	18827.62
2454	Asellia italosomalica	18725960528	18725.96
2455	Lorentzimys nouhuysi	18696693866	18696.69
2456	Grammomys macmillani	18666350653	18666.35
2457	Oxymycterus roberti	18641055785	18641.06

2458	Prosciurillus murinus	18625170112	18625.17
2459	Martes gwatkinsii	18619686018	18619.69
2460	Phyllomys blainvillii	18619613966	18619.61
2461	Sorex granarius	18618540194	18618.54
2462	Crocidura beatus	18605575150	18605.58
2463	Petaurista magnificus	18602879562	18602.88
2464	Dasyurus maculatus	18564541574	18564.54
2465	Otomops formosus	18549927126	18549.93
2466	Glyphonycteris daviesi	18530314535	18530.31
2467	Rattus fuscipes	18503289246	18503.29
2468	Dendromus kivu	18499652522	18499.65
2469	Platacanthomys lasiurus	18464180192	18464.18
2470	Stenocephalemys griseicauda	18451160322	18451.16
2471	Euneomys petersoni	18428576804	18428.58
2472	Peromyscus californicus	18330982598	18330.98
2473	Craseomys andersoni	18288926646	18288.93
2474	Hylaeamys oniscus	18263052574	18263.05
2475	Abrocoma bennettii	18222013669	18222.01
2476	Dolichotis patagonum	18219056749	18219.06
2477	Peromyscus eremicus	18203412254	18203.41
2478	Dromiciops gliroides	18193027474	18193.03
2479	Habromys lophurus	18110157803	18110.16
2480	Neotoma micropus	18089088384	18089.09
2481	Macruromys major	18061083396	18061.08
2482	Crocidura pachyura	18058955639	18058.96
2483	Oryx gazella	18057127653	18057.13
2484	Neotamias amoenus	18050799331	18050.80
2485	Molossus currentium	18030945374	18030.95
2486	Microsciurus flaviventer	18022160227	18022.16
2487	Necromys amoenus	17998516537	17998.52
2488	Hipposideros pomona	17938536909	17938.54
2489	Pteropus pumilus	17933901125	17933.90
2490	Crocidura lea	17926020342	17926.02
2491	Delomys sublineatus	17920906305	17920.91
2492	Ctenomys frater	17917401077	17917.40
2493	Georychus capensis	17906903180	17906.90
2494	Rattus verecundus	17860719881	17860.72
2495	Oligoryzomys griseolus	17839099807	17839.10
2496	Diclidurus scutatus	17829917759	17829.92
2497	Crocidura religiosa	17827724877	17827.72
2498	Thomasomys oreas	17826124805	17826.12

2499	Reithrodontomys chrysopsis	17811902721	17811.90
2500	Perognathus fasciatus	17796045539	17796.05
2501	Podomys floridanus	17786672056	17786.67
2502	Scapanus townsendii	17774312952	17774.31
2503	Cryptomys hottentotus	17761058583	17761.06
2504	Cercopithecus diana	17758631919	17758.63
2505	Zapus trinotatus	17754009575	17754.01
2506	Ozimops ridei	17740968748	17740.97
2507	Ochotona princeps	17721704277	17721.70
2508	Rhinolophus robinsoni	17607838017	17607.84
2509	Semnopithecus johnii	17541695439	17541.70
2510	Neotamias townsendii	17496598149	17496.60
2511	Gerbilliscus inclusus	17470341921	17470.34
2512	Neotoma chrysomelas	17427617708	17427.62
2513	Paranyctimene tenax	17370257880	17370.26
2514	Eligmodontia morgani	17339663461	17339.66
2515	Pseudohydromys ellermani	17336048813	17336.05
2516	Vulpes zerda	17323580672	17323.58
2517	Chiropotes satanas	17322062834	17322.06
2518	Murina puta	17313030502	17313.03
2519	Anourosorex yamashinai	17313030500	17313.03
2520	Apodemus semotus	17313029408	17313.03
2521	Saguinus ursulus	17295800831	17295.80
2522	Myonycteris relicta	17279769601	17279.77
2523	Hylaeamys laticeps	17275025276	17275.03
2524	Atelerix sclateri	17261357634	17261.36
2525	Crunomys melanius	17199665633	17199.67
2526	Spalacopus cyanus	17199273498	17199.27
2527	Niviventer coninga	17198902287	17198.90
2528	Myotis csorbai	17154406068	17154.41
2529	Steatomys bocagei	17127779739	17127.78
2530	Osgoodomys banderanus	17091985502	17091.99
2531	Lycaon pictus	17065403059	17065.40
2532	Thylamys velutinus	17047967756	17047.97
2533	Gerbilliscus gambiana	16993330091	16993.33
2534	Perameles nasuta	16904401398	16904.40
2535	Tupaia salatana	16856210488	16856.21
2536	Sapajus xanthosternos	16835841741	16835.84
2537	Trachypithecus selangorensis	16825524630	16825.52
2538	Tomopeas ravus	16786092345	16786.09
2539	Akodon dolores	16779280430	16779.28

2540	Marmosa lepida	16774246960	16774.25
2541	Saimiri boliviensis	16751250471	16751.25
2542	Glauconycteris egeria	16742176059	16742.18
2543	Crocidura nigricans	16738952586	16738.95
2544	Microtus guatemalensis	16722519087	16722.52
2545	Akodon affinis	16719569283	16719.57
2546	Hylomyscus carillus	16621016991	16621.02
2547	Pteropus rufus	16610471099	16610.47
2548	Pogonomys loriae	16602537979	16602.54
2549	Acomys spinosissimus	16581430018	16581.43
2550	Semnopithecus vetulus	16453213035	16453.21
2551	Murina aenea	16448690268	16448.69
2552	Oecomys speciosus	16404561081	16404.56
2553	Octodon degus	16355191910	16355.19
2554	Crocidura goliath	16333965588	16333.97
2555	Nyctimene keasti	16303701538	16303.70
2556	Lemniscomys linulus	16247264875	16247.26
2557	Pteropus scapulatus	16247080862	16247.08
2558	Octodon bridgesi	16211144822	16211.14
2559	Sciurus pyrrhinus	16201774003	16201.77
2560	Ctenomys magellanicus	16200778107	16200.78
2561	Paradipus ctenodactylus	16171428725	16171.43
2562	Mallomys aroaensis	16111294692	16111.29
2563	Acomys ignitus	16108538921	16108.54
2564	Neotoma macrotis	16104765800	16104.77
2565	Akodon subfuscus	16064729191	16064.73
2566	Dendrolagus goodfellowi	16058509448	16058.51
2567	Rattus niobe	16055559849	16055.56
2568	Thallomys loringi	15992681983	15992.68
2569	Phyllotis andium	15938033147	15938.03
2570	Ratufa macroura	15937967661	15937.97
2571	Callosciurus albescens	15929707380	15929.71
2572	Bos javanicus	15928865727	15928.87
2573	Rattus marmosurus	15927486897	15927.49
2574	Sorex ventralis	15907544761	15907.54
2575	Microtus qazvinensis	15886175922	15886.18
2576	Dasymys nudipes	15879945082	15879.95
2577	Pseudohydromys fuscus	15823586526	15823.59
2578	Sapajus cay	15803222899	15803.22
2579	Ammospermophilus leucurus	15800214700	15800.21
2580	Hyomys goliath	15787080206	15787.08

2581	Scotorepens orion	15783296635	15783.30
2582	Praomys delectorum	15780121148	15780.12
2583	Pseudochirulus larvatus	15777896432	15777.90
2584	Pudu puda	15762795636	15762.80
2585	Dendrolagus notatus	15761742253	15761.74
2586	Lycalopex sechurae	15744990075	15744.99
2587	Pogonomys sylvestris	15731587075	15731.59
2588	Neophascogale lorentzii	15722400268	15722.40
2589	Hyomys dammermani	15720061357	15720.06
2590	Thomasomys niveipes	15703960340	15703.96
2591	Presbytis thomasi	15676253868	15676.25
2592	Sundamys infraluteus	15648100854	15648.10
2593	Akodon caenosus	15647466746	15647.47
2594	Phyllotis limatus	15632193247	15632.19
2595	Perognathus merriami	15613307491	15613.31
2596	Notamacropus rufogriseus	15577390754	15577.39
2597	Macaca silenus	15397804911	15397.80
2598	Sminthopsis murina	15381009966	15381.01
2599	Hylobates muelleri	15376485333	15376.49
2600	Chelemys megalonyx	15372237750	15372.24
2601	Dermanura rosenbergi	15368442450	15368.44
2602	Malacothrix typica	15304214241	15304.21
2603	Graphiurus angolensis	15259384124	15259.38
2604	Macaca sinica	15185436033	15185.44
2605	Rattus lutreolus	15172518829	15172.52
2606	Chaetodipus californicus	15112456595	15112.46
2607	Oryx dammah	15103928169	15103.93
2608	Sylvisorex granti	15101041848	15101.04
2609	Dendromus insignis	15083549886	15083.55
2610	Myotis auriculus	15078999364	15079.00
2611	Crocidura bottegi	14944070466	14944.07
2612	Arielulus societatis	14922118500	14922.12
2613	Muntiacus putaoensis	14867773480	14867.77
2614	Rhipidomys emiliae	14853056477	14853.06
2615	Coccymys shawmayeri	14839817112	14839.82
2616	Capra nubiana	14833330817	14833.33
2617	Petauroides volans	14809273191	14809.27
2618	Histiotus magellanicus	14807312935	14807.31
2619	Thomasomys notatus	14794943147	14794.94
2620	Hylomyscus kerbispeterhansi	14787320270	14787.32
2621	Phenacomys intermedius	14773554584	14773.55

2622	Petaurus norfolcensis	14772426634	14772.43
2623	Rhinolophus keyensis	14694924148	14694.92
2624	Pseudohydromys murinus	14686716058	14686.72
2625	Hylomyscus endorobae	14656720380	14656.72
2626	Urocitellus richardsonii	14630638110	14630.64
2627	Microtus thomasi	14628344616	14628.34
2628	Miniopterus minor	14603398878	14603.40
2629	Hipposideros muscinus	14560859002	14560.86
2630	Emballonura raffrayana	14552177887	14552.18
2631	Ammodillus imbellis	14550680338	14550.68
2632	Tarsius fuscus	14491913096	14491.91
2633	Macaca maura	14485079080	14485.08
2634	Antechinus flavipes	14460862862	14460.86
2635	Hylobates abbotti	14401551922	14401.55
2636	Phalanger vestitus	14395288331	14395.29
2637	Tylomys watsoni	14354261821	14354.26
2638	Paradoxurus zeylonensis	14345461302	14345.46
2639	Myoprocta pratti	14329681313	14329.68
2640	Eptesicus floweri	14325458938	14325.46
2641	Neofelis diardi	14309599018	14309.60
2642	Hipposideros pendleburyi	14294064571	14294.06
2643	Dipodomys simulans	14292054933	14292.05
2644	Sigmodon alstoni	14254671146	14254.67
2645	Ochotona ladacensis	14230050863	14230.05
2646	Mallomys istapantap	14226109153	14226.11
2647	Peromyscus hylocetes	14214987510	14214.99
2648	Macronycteris commersoni	14213365699	14213.37
2649	Cebus kaapori	14135998965	14136.00
2650	Dinaromys bogdanovi	14133661154	14133.66
2651	Cratogeomys fumosus	14128078050	14128.08
2652	Vulpes velox	14116038008	14116.04
2653	Peromyscus nasutus	14115922027	14115.92
2654	Saguinus leucopus	14082255195	14082.26
2655	Murina fionae	14054582666	14054.58
2656	Murina florium	13970816001	13970.82
2657	Isothrix bistriata	13960121635	13960.12
2658	Chinchillula sahamae	13956233464	13956.23
2659	Scotophilus nucella	13948192513	13948.19
2660	Allactaga balikunica	13942038086	13942.04
2661	Pattonomys carrikeri	13938388007	13938.39
2662	Crocidura pullata	13928681607	13928.68

2663	Piliocolobus waldroni	13893425314	13893.43
2664	Papio papio	13854300772	13854.30
2665	Hylobates funereus	13795496062	13795.50
2666	Plecotus christii	13780409808	13780.41
2667	Glironia venusta	13744803365	13744.80
2668	Akodon simulator	13718133796	13718.13
2669	Platyrrhinus ismaeli	13707379686	13707.38
2670	Thomasomys dispar	13705980366	13705.98
2671	Cephalophus leucogaster	13683906556	13683.91
2672	Glauconycteris alboguttata	13598868476	13598.87
2673	Cercartetus nanus	13553908752	13553.91
2674	Miniopterus sororculus	13549058231	13549.06
2675	Phyllotis haggardi	13541301329	13541.30
2676	Hipposideros sumbae	13477073173	13477.07
2677	Rhinolophus ruwenzorii	13455960620	13455.96
2678	Brachyteles arachnoides	13455735025	13455.74
2679	Necromys lactens	13374191740	13374.19
2680	Hylobates pileatus	13361405535	13361.41
2681	Leopoldamys neilli	13354842909	13354.84
2682	Cricetulus lama	13334692350	13334.69
2683	Brucepattersonius soricinus	13321820156	13321.82
2684	Saguinus oedipus	13311254167	13311.25
2685	Sciurus yucatanensis	13218845294	13218.85
2686	Acomys mullah	13212393224	13212.39
2687	Chalinolobus dwyeri	13203228138	13203.23
2688	Myoictis melas	13188944977	13188.94
2689	Oxymycterus rufus	13129554222	13129.55
2690	Cercocebus agilis	13123328192	13123.33
2691	Taeromys celebensis	13111587517	13111.59
2692	Sciurillus pusillus	13106274107	13106.27
2693	Brucepattersonius iheringi	13092342696	13092.34
2694	Protoxerus aubinnii	13082745286	13082.75
2695	Sturnira koopmanhilli	13080231265	13080.23
2696	Dendromus messorius	13050642457	13050.64
2697	Nycticebus kayan	13039131443	13039.13
2698	Euryoryzomys nitidus	12971217856	12971.22
2699	Phoniscus papuensis	12855341079	12855.34
2700	Mastomys awashensis	12847043373	12847.04
2701	Miniopterus majori	12843311025	12843.31
2702	Nanger soemmerringii	12841841471	12841.84
2703	Thylamys cinderella	12814479251	12814.48

2704	Muntiacus vuquangensis	12789530628	12789.53
2705	Cistugo lesueuri	12778582461	12778.58
2706	Myotis simus	12765992881	12765.99
2707	Microtus paradoxus	12765073775	12765.07
2708	Paranyctimene raptor	12760745841	12760.75
2709	Lophuromys simensis	12754401067	12754.40
2710	Paracrocidura schoutedeni	12743162134	12743.16
2711	Cricetulus tibetanus	12735870581	12735.87
2712	Necromys benefactus	12712634932	12712.63
2713	Crocidura horsfieldii	12707762144	12707.76
2714	Microtus brachycercus	12679041573	12679.04
2715	Talpa caucasica	12662617423	12662.62
2716	Macaca cyclopis	12635171635	12635.17
2717	Petinomys vordermanni	12612239114	12612.24
2718	Nyctimene rabori	12607100897	12607.10
2719	Neotoma fuscipes	12586633288	12586.63
2720	Mesocricetus raddei	12548335188	12548.34
2721	Akodon juninensis	12543005873	12543.01
2722	Pseudochirulus mayeri	12515549727	12515.55
2723	Rhinolophus capensis	12472570762	12472.57
2724	Gerbilliscus phillipsi	12456432209	12456.43
2725	Gerbillus floweri	12446807733	12446.81
2726	Crocidura selina	12411341469	12411.34
2727	Zelotomys woosnami	12411092426	12411.09
2728	Otomys karoensis	12406679415	12406.68
2729	Megaerops kusnotoi	12371789285	12371.79
2730	Aotus zonalis	12366484421	12366.48
2731	Calomys boliviae	12347237614	12347.24
2732	Akodon fumeus	12323628352	12323.63
2733	Proechimys cuvieri	12283324761	12283.32
2734	Oecomys superans	12281903704	12281.90
2735	Euroscaptor subanura	12281736762	12281.74
2736	Nephelomys levipes	12262213388	12262.21
2737	Sorex mediopua	12259219155	12259.22
2738	Crocidura nigripes	12234102534	12234.10
2739	Micropteropus intermedius	12230991665	12230.99
2740	Funambulus sublineatus	12221478100	12221.48
2741	Scoteanax rueppellii	12203907608	12203.91
2742	Isoodon macrourus	12171366537	12171.37
2743	Thomasomys princeps	12117482856	12117.48
2744	Peromyscus guatemalensis	12111334239	12111.33

2745	Piliocolobus oustaleti	12105651932	12105.65
2746	Gracilinanus dryas	12095514422	12095.51
2747	Eothenomys proditor	12067782431	12067.78
2748	Stylodipus sungorus	12048497581	12048.50
2749	Aeretes melanopterus	11967588931	11967.59
2750	Makalata macrura	11927248530	11927.25
2751	Marmosops paulensis	11895151300	11895.15
2752	Apomys microdon	11884673137	11884.67
2753	Chrotomys mindorensis	11876670586	11876.67
2754	Myotis ater	11874720398	11874.72
2755	Lagothrix lagothricha	11864490711	11864.49
2756	Latidens salimalii	11810070394	11810.07
2757	Allactaga hotsoni	11782369863	11782.37
2758	Akodon dayi	11774094303	11774.09
2759	Crocidura ludia	11766163806	11766.16
2760	Mammelomys lanosus	11742996927	11743.00
2761	Platyrrhinus fusciventris	11723385222	11723.39
2762	Desmalopex leucopterus	11718161551	11718.16
2763	Arielulus torquatus	11651329485	11651.33
2764	Petinomys crinitus	11636747824	11636.75
2765	Reithrodontomys tenuirostris	11621678057	11621.68
2766	Akodon spegazzinii	11611964698	11611.96
2767	Hybomys planifrons	11583544246	11583.54
2768	Crocidura phanluongi	11533699321	11533.70
2769	Dendrohyrax validus	11522179200	11522.18
2770	Cryptotis equatoris	11520001576	11520.00
2771	Dolichotis salinicola	11505286518	11505.29
2772	Macaca tonkeana	11496536747	11496.54
2773	Pipistrellus angulatus	11484981935	11484.98
2774	Phenacomys ungava	11457483560	11457.48
2775	Otomys simiensis	11431854936	11431.85
2776	Petinomys mindanensis	11431674298	11431.67
2777	Chrysospalax trevelyani	11424046424	11424.05
2778	Murina walstoni	11341881988	11341.88
2779	Presbytis frontata	11306721302	11306.72
2780	Mastomys pernanus	11304950071	11304.95
2781	Onychomys arenicola	11302520890	11302.52
2782	Crocidura douceti	11293053405	11293.05
2783	Phyllotis wolffsohni	11277474111	11277.47
2784	Sus philippensis	11266588349	11266.59
2785	Perognathus parvus	11264404897	11264.40

2786	Macrotus californicus	11256413724	11256.41
2787	Baeodon gracilis	11239776981	11239.78
2788	Dendromus lachaisei	11236599684	11236.60
2789	Funisciurus isabella	11224213120	11224.21
2790	Neotoma albigula	11217972993	11217.97
2791	Cerradomys scotti	11215895361	11215.90
2792	Clethrionomys californicus	11209241840	11209.24
2793	Neotomodon alstoni	11120001943	11120.00
2794	Spalax zemni	11059478331	11059.48
2795	Delomys collinus	11047597013	11047.60
2796	Hipposideros curtus	11032216968	11032.22
2797	Psammomys vexillaris	11031537216	11031.54
2798	Spermophilus ralli	11029576154	11029.58
2799	Loxodontomys pikumche	10996928876	10996.93
2800	Grammomys dryas	10996640689	10996.64
2801	Ctenodactylus gundi	10968958945	10968.96
2802	Tarsius lariang	10831226269	10831.23
2803	Protochromys fellowsi	10827735933	10827.74
2804	Paraxerus vexillarius	10805778753	10805.78
2805	Chiropodomys major	10795790824	10795.79
2806	Pipistrellus endoi	10792762338	10792.76
2807	Rousettus spinalatus	10777695293	10777.70
2808	Apodemus alpicola	10772072969	10772.07
2809	Peropteryx pallidoptera	10746001618	10746.00
2810	Trinomys dimidiatus	10722172991	10722.17
2811	Paracrocidura maxima	10711791612	10711.79
2812	Cyttarops alecto	10701610856	10701.61
2813	Taphozous hildegardeae	10695145171	10695.15
2814	Rhinolophus formosae	10686626799	10686.63
2815	Eumops trumbulli	10628221216	10628.22
2816	Crocidura katinka	10627460881	10627.46
2817	Neotamias quadrivittatus	10615781917	10615.78
2818	Madoqua piacentinii	10591126000	10591.13
2819	Crocidura baileyi	10585140129	10585.14
2820	Rhinolophus damarensis	10580611378	10580.61
2821	Antechinus swainsonii	10538669578	10538.67
2822	Bubalus depressicornis	10537188619	10537.19
2823	Chrysospalax villosus	10523541286	10523.54
2824	Sorex haydeni	10505947101	10505.95
2825	Cebus brunneus	10504485277	10504.49
2826	Chaetodipus penicillatus	10493933059	10493.93

2827	Peromyscus furvus	10453575203	10453.58
2828	Graphiurus ocularis	10405826426	10405.83
2829	Myosciurus pumilio	10395822141	10395.82
2830	Cercopithecus erythrotis	10372775435	10372.78
2831	Dasyprocta iacki	10361856478	10361.86
2832	Phascogale tapoatafa	10358092748	10358.09
2833	Chodsigoa smithii	10326020477	10326.02
2834	Tolypeutes matacus	10301150388	10301.15
2835	Peromyscus fraterculus	10300401091	10300.40
2836	Chaetodipus nelsoni	10298304536	10298.30
2837	Fukomys foxi	10288584552	10288.58
2838	Anoura fistulata	10279413264	10279.41
2839	Cebus olivaceus	10267513203	10267.51
2840	Cavia magna	10260783628	10260.78
2841	Handleyomys chapmani	10254226334	10254.23
2842	Galea comes	10186128258	10186.13
2843	Philander mondolfii	10169008586	10169.01
2844	Proechimys simonsi	10167154375	10167.15
2845	Microgale dobsoni	10128797165	10128.80
2846	Pattonomys semivillosus	10095166703	10095.17
2847	Scotorepens sanborni	10085271719	10085.27
2848	Scarturus vinogradovi	10071842305	10071.84
2849	Echimys chrysurus	10052031143	10052.03
2850	Atelocynus microtis	10050140835	10050.14
2851	Cryptotis montivaga	10035120336	10035.12
2852	Dipodomys spectabilis	9988973921	9988.97
2853	Gerbillus cosensis	9980711134	9980.71
2854	Ammotragus lervia	9947102264	9947.10
2855	Nomascus leucogenys	9897854471	9897.85
2856	Oligoryzomys brendae	9847113506	9847.11
2857	Chaetodipus intermedius	9832101083	9832.10
2858	Rhinolophus guineensis	9819389121	9819.39
2859	Sigmodon alleni	9787805170	9787.81
2860	Chaetomys subspinosus	9786557048	9786.56
2861	Ochotona mantchurica	9778101555	9778.10
2862	Megasorex gigas	9745547660	9745.55
2863	Thomasomys cinnameus	9721398229	9721.40
2864	Melomys dollmani	9690816854	9690.82
2865	Gazella cuvieri	9686847937	9686.85
2866	Hylomyscus baeri	9595536450	9595.54
2867	Rhinolophus rufus	9592239096	9592.24

2868	Glauconycteris curryae	9587023919	9587.02
2869	Funisciurus bayonii	9539179110	9539.18
2870	Oecomys flavicans	9521667226	9521.67
2871	Thylamys karimii	9471722004	9471.72
2872	Oecomys paricola	9458790267	9458.79
2873	Coendou insidiosus	9427792208	9427.79
2874	Selevinia betpakdalaensis	9362689148	9362.69
2875	Sciurus aberti	9338900643	9338.90
2876	Crocidura montis	9319328379	9319.33
2877	Elephantulus revoilii	9316318331	9316.32
2878	Hylomyscus anselli	9296483950	9296.48
2879	Ctenomys minutus	9283766436	9283.77
2880	Crocidura erica	9276714678	9276.71
2881	Oligoryzomys delticola	9235967564	9235.97
2882	Phyllotis amicus	9235727635	9235.73
2883	Hipposideros camerunensis	9229514619	9229.51
2884	Vespadelus troughtoni	9227258109	9227.26
2885	Anoura cadenai	9213986474	9213.99
2886	Nyctophilus microdon	9201207177	9201.21
2887	Planigale maculata	9175753702	9175.75
2888	Apomys musculus	9171176406	9171.18
2889	Nycticebus borneanus	9131072772	9131.07
2890	Rhipidomys cariri	9118591717	9118.59
2891	Myotis federatus	9110123207	9110.12
2892	Allactaga tetradactyla	9061277401	9061.28
2893	Phloeomys cumingi	9059129394	9059.13
2894	Microcavia niata	9052832887	9052.83
2895	Raphicerus melanotis	9047358984	9047.36
2896	Sylvisorex lunaris	9046717146	9046.72
2897	Scapteromys tumidus	9007146633	9007.15
2898	Reithrodontomys zacatecae	8990121638	8990.12
2899	Presbytis fredericae	8960317982	8960.32
2900	Habromys simulatus	8957241202	8957.24
2901	Sorex preblei	8912145211	8912.15
2902	Dacnomys millardi	8888256440	8888.26
2903	Eliurus myoxinus	8874928912	8874.93
2904	Cynomops milleri	8863802735	8863.80
2905	Myotis rufopictus	8835779406	8835.78
2906	Cercopithecus wolfi	8799943139	8799.94
2907	Talpa stankovici	8799782982	8799.78
2908	Proechimys brevicauda	8798620712	8798.62

2909	Rhinolophus canuti	8794384467	8794.38
2910	Crocidura pasha	8780618113	8780.62
2911	Otomys irroratus	8771045979	8771.05
2912	Neotamias dorsalis	8754954975	8754.95
2913	Haeromys minahassae	8746940096	8746.94
2914	Leopoldamys diwangkarai	8742319743	8742.32
2915	Gazella gazella	8725662533	8725.66
2916	Margaretamys beccarii	8720385636	8720.39
2917	Xerospermophilus tereticaudus	8713153497	8713.15
2918	Allochrocebus lhoesti	8702849426	8702.85
2919	Neomicroxus latebricola	8683275351	8683.28
2920	Vespadelus pumilus	8682207832	8682.21
2921	Mops leucogaster	8666233668	8666.23
2922	Nyctimene albiventer	8665341666	8665.34
2923	Taphozous achates	8646584514	8646.58
2924	Mindomys hammondi	8646522511	8646.52
2925	Chaetodipus fallax	8639872134	8639.87
2926	Piliocolobus lulindicus	8599935189	8599.94
2927	Octodon lunatus	8582916054	8582.92
2928	Sekeetamys calurus	8579284382	8579.28
2929	Thomasomys pyrrhonotus	8570011431	8570.01
2930	Microtus felteni	8564476096	8564.48
2931	Tremarctos ornatus	8563654310	8563.65
2932	Peromyscus crinitus	8558975916	8558.98
2933	Vombatus ursinus	8543639142	8543.64
2934	Mustela russelliana	8511616342	8511.62
2935	Sciurus gilvigularis	8501171719	8501.17
2936	Sorex oreopolus	8487123655	8487.12
2937	Crocidura tenuis	8471344769	8471.34
2938	Euroscaptor micrura	8441852732	8441.85
2939	Moschus cupreus	8441386021	8441.39
2940	Dymecodon pilirostris	8437289416	8437.29
2941	Scotorepens greyii	8432639104	8432.64
2942	Fukomys damarensis	8430971361	8430.97
2943	Osphranter robustus	8421501395	8421.50
2944	Sorex sonomae	8414455110	8414.46
2945	Hesperoptenus tomesi	8308470907	8308.47
2946	Sturnira mordax	8292320847	8292.32
2947	Steatomys opimus	8287851218	8287.85
2948	Calomys hummelincki	8256646655	8256.65
2949	Petaurus australis	8237132422	8237.13

2950	Phascolosorex doriae	8229372536	8229.37
2951	Nycticeius cubanus	8226760568	8226.76
2952	Marmota caligata	8222174966	8222.17
2953	Akodon mimus	8182744683	8182.74
2954	Myotis borneoensis	8181850866	8181.85
2955	Thomasomys silvestris	8178209830	8178.21
2956	Ozimops lumsdenae	8143470908	8143.47
2957	Otomys unisulcatus	8139952902	8139.95
2958	Komodomys rintjanus	8139707810	8139.71
2959	Melomys cervinipes	8124574242	8124.57
2960	Aepyprymnus rufescens	8097902922	8097.90
2961	Rattus pococki	8097462334	8097.46
2962	Alticola roylei	8064479198	8064.48
2963	Oxymycterus caparoae	8056838559	8056.84
2964	Chalinolobus tuberculatus	8055395393	8055.40
2965	Philander andersoni	8049036333	8049.04
2966	Cebus leucocephalus	8045968681	8045.97
2967	Chionomys roberti	8044938790	8044.94
2968	Ateles belzebuth	8044923211	8044.92
2969	Mydaus javanensis	8043821076	8043.82
2970	Oreoryzomys balneator	8041510227	8041.51
2971	Crocidura niobe	8036120215	8036.12
2972	Trinomys albispinus	8030152884	8030.15
2973	Oligoryzomys chacoensis	8020627153	8020.63
2974	Micronomus norfolkensis	8018775230	8018.78
2975	Spalax uralensis	7993348552	7993.35
2976	Crocidura caspica	7934344421	7934.34
2977	Oligoryzomys arenalis	7928974184	7928.97
2978	Hemicentetes nigriceps	7878945449	7878.95
2979	Sorex ixtlanensis	7878671465	7878.67
2980	Funisciurus lemniscatus	7826775171	7826.78
2981	Hylobates albibarbis	7814093502	7814.09
2982	Thomasomys daphne	7813683503	7813.68
2983	Thomasomys popayanus	7804890552	7804.89
2984	Callosciurus quinquestriatus	7789999374	7790.00
2985	Myotis australis	7786604727	7786.60
2986	Papagomys armandvillei	7777337467	7777.34
2987	Cryptotis brachyonyx	7765239450	7765.24
2988	Pongo pygmaeus	7752643652	7752.64
2989	Mysateles melanurus	7737367475	7737.37
2990	Artibeus hirsutus	7721147178	7721.15

2991	Chalinolobus nigrogriseus	7703606005	7703.61
2992	Thomasomys praetor	7703441558	7703.44
2993	Monodelphis glirina	7695292637	7695.29
2994	Hylomyscus denniae	7684324284	7684.32
2995	Myotis izecksohni	7668493791	7668.49
2996	Hemicentetes semispinosus	7661697568	7661.70
2997	Oxymycterus delator	7640373953	7640.37
2998	Nesolagus netscheri	7634283371	7634.28
2999	Lophuromys nudicaudus	7615702643	7615.70
3000	Microtus cabrerae	7615567993	7615.57
3001	Dactylopsila megalura	7609129548	7609.13
3002	Ctenomys opimus	7574606546	7574.61
3003	Lophocebus aterrimus	7570816841	7570.82
3004	Myotis montivagus	7566914767	7566.91
3005	Trichosurus caninus	7560002682	7560.00
3006	Notamacropus parryi	7532429679	7532.43
3007	Marmosops bishopi	7525728784	7525.73
3008	Neotoma lepida	7524498350	7524.50
3009	Ctenomys saltarius	7506552516	7506.55
3010	Reithrodon typicus	7499378212	7499.38
3011	Myosorex sclateri	7492209628	7492.21
3012	Pseudomys gracilicaudatus	7490221375	7490.22
3013	Phaenomys ferrugineus	7476056833	7476.06
3014	Proechimys chrysaeolus	7475349591	7475.35
3015	Crocidura somalica	7466878326	7466.88
3016	Gerbillus aquilus	7458656215	7458.66
3017	Rhabdomys pumilio	7438423076	7438.42
3018	Nomascus gabriellae	7437606924	7437.61
3019	Arvicanthis blicki	7424430039	7424.43
3020	Cryptotis medellinia	7389857903	7389.86
3021	Laephotis angolensis	7365569050	7365.57
3022	Thylamys pulchellus	7361196444	7361.20
3023	Notamacropus dorsalis	7329325344	7329.33
3024	Sciurus nayaritensis	7329158341	7329.16
3025	Dipodomys deserti	7320004289	7320.00
3026	Gerbillus watersi	7317189056	7317.19
3027	Oligoryzomys microtis	7301312919	7301.31
3028	Cryptotis thomasi	7297642324	7297.64
3029	Marmosa phaea	7274503542	7274.50
3030	Rattus tunneyi	7255985633	7255.99
3031	Prosciurillus leucomus	7244943196	7244.94

3032	Microtus quasiater	7239876960	7239.88
3033	Nyctophilus bifax	7237737856	7237.74
3034	Dactylomys dactylinus	7233974022	7233.97
3035	Anourosorex assamensis	7224574484	7224.57
3036	Meriones chengi	7208848397	7208.85
3037	Trinomys gratiosus	7206517837	7206.52
3038	Thomasomys auricularis	7183795440	7183.80
3039	Akodon lindberghi	7182752139	7182.75
3040	Pseudomys delicatulus	7180687239	7180.69
3041	Batomys salomonseni	7171139587	7171.14
3042	Loris tardigradus	7157022973	7157.02
3043	Niviventer lepturus	7154720940	7154.72
3044	Mazama rufina	7150675384	7150.68
3045	Thomasomys paramorum	7126534516	7126.53
3046	Cratogeomys castanops	7078399102	7078.40
3047	Eliurus tanala	7078259789	7078.26
3048	Cercopithecus hamlyni	7060461750	7060.46
3049	Scotophilus trujilloi	7044053519	7044.05
3050	Eliurus webbi	7037671854	7037.67
3051	Bubalus quarlesi	7031511270	7031.51
3052	Maxomys bartelsii	7008514120	7008.51
3053	Oecomys cleberi	6997874026	6997.87
3054	Mops leucostigma	6996709802	6996.71
3055	Rusa timorensis	6983625155	6983.63
3056	Chaetodipus formosus	6982214140	6982.21
3057	Eliurus minor	6946370145	6946.37
3058	Sminthopsis crassicaudata	6930252502	6930.25
3059	Reithrodontomys brevirostris	6919480285	6919.48
3060	Thomasomys caudivarius	6899240359	6899.24
3061	Salpingotus pallidus	6898144720	6898.14
3062	Dobsonia minor	6895068896	6895.07
3063	Crocidura brunnea	6880664884	6880.66
3064	Mormopterus minutus	6877617722	6877.62
3065	Nycteris parisii	6874266040	6874.27
3066	Nyctimene robinsoni	6864441575	6864.44
3067	Falsistrellus petersi	6861203385	6861.20
3068	Thylogale stigmatica	6855727751	6855.73
3069	Microcebus lehilahytsara	6845987319	6845.99
3070	Aotus vociferans	6845405988	6845.41
3071	Melomys burtoni	6844781656	6844.78
3072	Thomasomys hylophilus	6841385898	6841.39

3073	Ovis canadensis	6836913766	6836.91
3074	Sicista napaea	6832858596	6832.86
3075	Cercopithecus cephus	6826073618	6826.07
3076	Antechinus agilis	6813358351	6813.36
3077	Pteropus neohibernicus	6813024651	6813.02
3078	Herpestes flavescens	6794158002	6794.16
3079	Lutreolina massoia	6784467371	6784.47
3080	Dipodomys heermanni	6772586501	6772.59
3081	Myosorex meesteri	6772526958	6772.53
3082	Graomys domorum	6763234922	6763.23
3083	Tragelaphus angasii	6752028889	6752.03
3084	Dipodomys agilis	6745492527	6745.49
3085	Cebus cuscinus	6734337105	6734.34
3086	Sigmodon ochrognathus	6730152833	6730.15
3087	Pseudochirulus caroli	6721722294	6721.72
3088	Genetta bourloni	6706211052	6706.21
3089	Gerbillus juliani	6693567909	6693.57
3090	Hylaeamys tatei	6684656291	6684.66
3091	Microtus schelkovnikovi	6672885542	6672.89
3092	Petaurista nobilis	6667319095	6667.32
3093	Allenopithecus nigroviridis	6661900408	6661.90
3094	Bradypus torquatus	6628884055	6628.88
3095	Crocidura crenata	6614940402	6614.94
3096	Rhynchocyon petersi	6602533748	6602.53
3097	Euoticus elegantulus	6588626773	6588.63
3098	Crocidura macmillani	6578271636	6578.27
3099	Chionomys gud	6565342141	6565.34
3100	Notiosorex evotis	6543150481	6543.15
3101	Cynomys leucurus	6541835916	6541.84
3102	Arborimus albipes	6526422291	6526.42
3103	Gerbillus somalicus	6516964315	6516.96
3104	Akodon aerosus	6510546625	6510.55
3105	Tupaia hypochrysa	6510017487	6510.02
3106	Cerradomys maracajuensis	6502912713	6502.91
3107	Pelomys minor	6491973360	6491.97
3108	Crocidura zaphiri	6487958616	6487.96
3109	Lophuromys brevicaudus	6482469749	6482.47
3110	Hipposideros hypophyllus	6475368478	6475.37
3111	Neoromicia matroka	6446053537	6446.05
3112	Hemitragus jemlahicus	6431530595	6431.53
3113	Macrotarsomys bastardi	6427448889	6427.45

3114	Muntiacus gongshanensis	6427394255	6427.39
3115	Heteromys catopterius	6426706899	6426.71
3116	Rhabdomys bechuanae	6418836926	6418.84
3117	Akodon iniscatus	6409498850	6409.50
3118	Cynomys gunnisoni	6405426766	6405.43
3119	Petaurillus hosei	6395446825	6395.45
3120	Capra pyrenaica	6377316507	6377.32
3121	Platyrrhinus chocoensis	6376687088	6376.69
3122	Chinchilla chinchilla	6369739253	6369.74
3123	Calcochloris obtusirostris	6369730155	6369.73
3124	Crocidura fumosa	6367142798	6367.14
3125	Perognathus inornatus	6340765322	6340.77
3126	Tylomys mirae	6339942886	6339.94
3127	Platyrrhinus nitelinea	6329257535	6329.26
3128	Nyctimene aello	6323792853	6323.79
3129	Marmosa rubra	6306499745	6306.50
3130	Thomasomys emeritus	6301290898	6301.29
3131	Handleyomys melanotis	6295962617	6295.96
3132	Bullimus luzonicus	6287535941	6287.54
3133	Pteropus macrotis	6266106159	6266.11
3134	Stenoderma rufum	6260583603	6260.58
3135	Bdeogale omnivora	6245403980	6245.40
3136	Brachyphylla cavernarum	6241919074	6241.92
3137	Cervus hanglu	6240861448	6240.86
3138	Scotorepens balstoni	6201757306	6201.76
3139	Phyllotis gerbillus	6191050107	6191.05
3140	Trachypithecus mauritius	6175356707	6175.36
3141	Diclidurus ingens	6160325914	6160.33
3142	Pseudohydromys eleanorae	6155265959	6155.27
3143	Notiomys edwardsii	6154156444	6154.16
3144	Sigmodon arizonae	6132560410	6132.56
3145	Cebus yuracus	6131120052	6131.12
3146	Eumops nanus	6128246881	6128.25
3147	Oecomys roberti	6092015232	6092.02
3148	Dryadonycteris capixaba	6088905550	6088.91
3149	Nasuella meridensis	6085276265	6085.28
3150	Paramelomys platyops	6080140027	6080.14
3151	Macrogalidia musschenbroekii	6061119353	6061.12
3152	Sorex shinto	6026088091	6026.09
3153	Presbytis femoralis	6025084993	6025.08
3154	Thomasomys erro	6008262646	6008.26

3155	Tupaia longipes	6003113643	6003.11
3156	Rhinolophus mcintyrei	5998580892	5998.58
3157	Hypsugo musciculus	5997821377	5997.82
3158	Crocidura nimbae	5991438381	5991.44
3159	Gazella spekei	5990722332	5990.72
3160	Sylvisorex vulcanorum	5985311985	5985.31
3161	Macaca sylvanus	5962450839	5962.45
3162	Monodelphis osgoodi	5926118560	5926.12
3163	Ateles chamek	5907136766	5907.14
3164	Irenomys tarsalis	5902652871	5902.65
3165	Thamnomys venustus	5902641008	5902.64
3166	Urocitellus elegans	5875476576	5875.48
3167	Sylvilagus obscurus	5867844569	5867.84
3168	Microtus nasarovi	5866107433	5866.11
3169	Dasypus sabanicola	5864664029	5864.66
3170	Rhinolophus smithersi	5855683290	5855.68
3171	Proechimys longicaudatus	5837634753	5837.63
3172	Oligoryzomys magellanicus	5821674021	5821.67
3173	Crocidura desperata	5818489236	5818.49
3174	Tanyuromys aphrastus	5800906415	5800.91
3175	Triaenops rufus	5794111159	5794.11
3176	Oecomys mamorae	5791135786	5791.14
3177	Oryzorictes hova	5767541653	5767.54
3178	Crocidura glassi	5757155804	5757.16
3179	Chaetodipus baileyi	5746650117	5746.65
3180	Thalpomys lasiotis	5736998015	5737.00
3181	Pipistrellus papuanus	5732055745	5732.06
3182	Myomyscus verreauxii	5729893324	5729.89
3183	Pipistrellus hanaki	5726339737	5726.34
3184	Monodelphis emiliae	5726069341	5726.07
3185	Neotamias umbrinus	5724431861	5724.43
3186	Moschus anhuiensis	5721836128	5721.84
3187	Heterogeomys cherriei	5719146832	5719.15
3188	Petaurillus kinlochii	5716830939	5716.83
3189	Rhipidomys austrinus	5712845003	5712.85
3190	Brucepattersonius griserufescens	5672336434	5672.34
3191	Graphiurus johnstoni	5670790640	5670.79
3192	Chaetodipus eremicus	5654548066	5654.55
3193	Microgale majori	5648978622	5648.98
3194	Plecturocebus discolor	5646727428	5646.73
3195	Handleyomys intectus	5639462688	5639.46

3196	Thomasomys bombycinus	5637645475	5637.65
3197	Nyctimene wrightae	5629577408	5629.58
3198	Presbytis percura	5620971878	5620.97
3199	Cebuella pygmaea	5614739201	5614.74
3200	Arborimus longicaudus	5608489401	5608.49
3201	Lophuromys kilonzoi	5604975347	5604.98
3202	Urocitellus columbianus	5599442977	5599.44
3203	Crossarchus ansorgei	5599225241	5599.23
3204	Acomys subspinosus	5588106973	5588.11
3205	Tarsius tarsier	5584607128	5584.61
3206	Microgale pusilla	5568232205	5568.23
3207	Thomasomys cinereus	5566330219	5566.33
3208	Dasyprocta variegata	5550386328	5550.39
3209	Pogonomelomys mayeri	5515362490	5515.36
3210	Pseudochirulus canescens	5509742563	5509.74
3211	Thomasomys ucucha	5492643867	5492.64
3212	Abrothrix manni	5467388179	5467.39
3213	Tupaia picta	5460483341	5460.48
3214	Antechinus stuartii	5448616802	5448.62
3215	Paragalago orinus	5442319067	5442.32
3216	Leontocebus nigricollis	5432686025	5432.69
3217	Proechimys quadruplicatus	5426586508	5426.59
3218	Mesocricetus newtoni	5418881678	5418.88
3219	Mesomys stimulax	5386810445	5386.81
3220	Callosciurus adamsi	5380457371	5380.46
3221	Prosciurillus alstoni	5366968169	5366.97
3222	Presbytis hosei	5350071468	5350.07
3223	Pygathrix nemaeus	5327267729	5327.27
3224	Paraechinus nudiventris	5326090890	5326.09
3225	Coelops robinsoni	5315530821	5315.53
3226	Phyllotis caprinus	5315319469	5315.32
3227	Cryptotis meridensis	5291785564	5291.79
3228	Brachyuromys betsileoensis	5288361402	5288.36
3229	Nephelomys keaysi	5264981516	5264.98
3230	Aconaemys sagei	5264815359	5264.82
3231	Echiothrix centrosa	5264592803	5264.59
3232	Ctenomys juris	5258297537	5258.30
3233	Caenolestes caniventer	5257639069	5257.64
3234	Myzopoda aurita	5255208024	5255.21
3235	Mazama chunyi	5253287573	5253.29
3236	Isoodon obesulus	5230791038	5230.79

3237	Meriones sacramenti	5216843775	5216.84
3238	Microgale brevicaudata	5212708880	5212.71
3239	Callicebus barbarabrownae	5204886028	5204.89
3240	Aselliscus tricuspidatus	5204461348	5204.46
3241	Sylvilagus transitionalis	5200773189	5200.77
3242	Thomasomys andersoni	5196115858	5196.12
3243	Marmosops pinheiroi	5166293683	5166.29
3244	Stenocephalemys albocaudata	5156997938	5157.00
3245	Hyosciurus heinrichi	5155624696	5155.62
3246	Brachyteles hypoxanthus	5154637608	5154.64
3247	Gerbillus amoenus	5153753048	5153.75
3248	Mops mops	5143481861	5143.48
3249	Chlorocebus djamdjamensis	5129611548	5129.61
3250	Ammospermophilus harrisii	5106440278	5106.44
3251	Cryptotis mexicana	5104279535	5104.28
3252	Microgale fotsifotsy	5100381429	5100.38
3253	Lophuromys medicaudatus	5098588971	5098.59
3254	Chaerephon jobimena	5092434116	5092.43
3255	Maxomys baeodon	5085231513	5085.23
3256	Pteropus conspicillatus	5073257459	5073.26
3257	Abeomelomys sevia	5065225264	5065.23
3258	Brachylagus idahoensis	5062332882	5062.33
3259	Otomops wroughtoni	5056970332	5056.97
3260	Neotamias merriami	5047632363	5047.63
3261	Lepus saxatilis	5041083583	5041.08
3262	Paremballonura atrata	5031598394	5031.60
3263	Microgale soricoides	5031345005	5031.35
3264	Eremoryzomys polius	5020918697	5020.92
3265	Macropus fuliginosus	5020449110	5020.45
3266	Pygathrix cinerea	5003423602	5003.42
3267	Equus ferus	4999671629	4999.67
3268	Sapajus flavius	4997724167	4997.72
3269	Thomasomys vulcani	4966795631	4966.80
3270	Otomys anchietae	4962379358	4962.38
3271	Potorous tridactylus	4960570657	4960.57
3272	Callicebus coimbrai	4934059322	4934.06
3273	Cryptotis nigrescens	4932383520	4932.38
3274	Glyphonycteris behnii	4924331069	4924.33
3275	Rhyncholestes raphanurus	4915419778	4915.42
3276	Oligoryzomys rupestris	4912148954	4912.15
3277	Microgale drouhardi	4908290557	4908.29

3278	Sundamys maxi	4905746719	4905.75
3279	Dendrolagus stellarum	4901754944	4901.75
3280	Proechimys canicollis	4897477542	4897.48
3281	Gerbillus acticola	4875100452	4875.10
3282	Chinchilla lanigera	4868170347	4868.17
3283	Hylomyscus parvus	4855480610	4855.48
3284	Crocidura bottegoides	4846157285	4846.16
3285	Gerbilliscus afra	4818608271	4818.61
3286	Hipposideros halophyllus	4803991410	4803.99
3287	Callicebus personatus	4783002144	4783.00
3288	Pygeretmus zhitkovi	4777038089	4777.04
3289	Oxymycterus inca	4721574417	4721.57
3290	Pseudohydromys patriciae	4715187378	4715.19
3291	Callospermophilus saturatus	4712300752	4712.30
3292	Ctenomys viperinus	4710609757	4710.61
3293	Ctenomys scagliai	4699691367	4699.69
3294	Ctenomys tuconax	4698853391	4698.85
3295	Rattus praetor	4685791914	4685.79
3296	Nesomys rufus	4670476360	4670.48
3297	Plecotus taivanus	4663067992	4663.07
3298	Otomys cuanzensis	4627715112	4627.72
3299	Liberiictis kuhni	4622854539	4622.85
3300	Microgale principula	4615703800	4615.70
3301	Taterillus petteri	4595251634	4595.25
3302	Aotus nancymaae	4588475898	4588.48
3303	Abrothrix illutea	4579447696	4579.45
3304	Emballonura furax	4566097741	4566.10
3305	Grammomys minnae	4564426448	4564.43
3306	Equus grevyi	4553188097	4553.19
3307	Crocidura tarfayensis	4550649953	4550.65
3308	Nesomys audeberti	4545716394	4545.72
3309	Huetia leucorhina	4542622242	4542.62
3310	Caenolestes convelatus	4535146522	4535.15
3311	Thomasomys fumeus	4534770473	4534.77
3312	Olallamys albicauda	4533583014	4533.58
3313	Sundasciurus brookei	4516868007	4516.87
3314	Rattus xanthurus	4505902575	4505.90
3315	Rhogeessa bickhami	4504510854	4504.51
3316	Dasypus pilosus	4502341109	4502.34
3317	Aethomys silindensis	4501895785	4501.90
3318	Rhipidomys modicus	4501887054	4501.89

3319	Cercartetus concinnus	4497415187	4497.42
3320	Taphozous hamiltoni	4496486412	4496.49
3321	Rhinopithecus bieti	4495535606	4495.54
3322	Microgale gymnorhyncha	4476443174	4476.44
3323	Otomys cheesmani	4472077653	4472.08
3324	Hyladelphys kalinowskii	4466805190	4466.81
3325	Microgale gracilis	4466296275	4466.30
3326	Ctenomys tucumanus	4462018467	4462.02
3327	Spilogale pygmaea	4453537319	4453.54
3328	Nomascus concolor	4443773318	4443.77
3329	Hypsugo eisentrauti	4441913835	4441.91
3330	Praomys coetzeei	4436826766	4436.83
3331	Gracilinanus emiliae	4432492199	4432.49
3332	Praomys degraaffi	4423962444	4423.96
3333	Callimico goeldii	4393329340	4393.33
3334	Tragelaphus derbianus	4376255528	4376.26
3335	Maxomys moi	4368853786	4368.85
3336	Ctenomys occultus	4361730002	4361.73
3337	Thomomys mazama	4353677131	4353.68
3338	Akodon pervalens	4349483606	4349.48
3339	Thomasomys kalinowskii	4348677900	4348.68
3340	Rhinolophus chiewkweeae	4347553022	4347.55
3341	Lagidium wolffsohni	4344789659	4344.79
3342	Nephelomys meridensis	4338782143	4338.78
3343	Mico melanurus	4338779171	4338.78
3344	Kobus leche	4337353184	4337.35
3345	Crocidura munissii	4331036306	4331.04
3346	Sorex pacificus	4326961375	4326.96
3347	Sus verrucosus	4315290970	4315.29
3348	Gerbillus perpallidus	4294959032	4294.96
3349	Microtus canicaudus	4281839628	4281.84
3350	Poelagus marjorita	4279907185	4279.91
3351	Urocitellus armatus	4278974803	4278.97
3352	Perognathus amplus	4258958267	4258.96
3353	Chilonatalus micropus	4238626785	4238.63
3354	Carterodon sulcidens	4233412711	4233.41
3355	Tarsius spectrumgurskyae	4229766413	4229.77
3356	Lophuromys melanonyx	4206089111	4206.09
3357	Gymnuromys roberti	4195737977	4195.74
3358	Hipposideros marisae	4193274964	4193.27
3359	Thomasomys australis	4190801851	4190.80

3360	Paremballonura tiavato	4185691781	4185.69
3361	Presbytis sabana	4160130024	4160.13
3362	Trachypithecus delacouri	4152524941	4152.52
3363	Ectophylla alba	4140848265	4140.85
3364	Galenomys garleppi	4134472803	4134.47
3365	Euryoryzomys lamia	4129134465	4129.13
3366	Suncus montanus	4124523643	4124.52
3367	Crocidura levicula	4113420837	4113.42
3368	Gorilla gorilla	4101517250	4101.52
3369	Phyllotis alisosiensis	4079662358	4079.66
3370	Ctenomys rionegrensis	4079460590	4079.46
3371	Coccymys ruemmleri	4067931564	4067.93
3372	Cryptotis colombiana	4066758220	4066.76
3373	Xenothrix mcgregori	4061001285	4061.00
3374	Natalus jamaicensis	4060781835	4060.78
3375	Ariteus flavescens	4060774751	4060.77
3376	Lasiurus degelidus	4060774751	4060.77
3377	Taeromys callitrichus	4055918010	4055.92
3378	Heteromys gaumeri	4050577655	4050.58
3379	Dipodomys stephensi	4050156381	4050.16
3380	Necromys lenguarum	4042665347	4042.67
3381	Elephantulus edwardii	4039300402	4039.30
3382	Oxymycterus wayku	4033040697	4033.04
3383	Ammospermophilus interpres	4029423229	4029.42
3384	Megadontomys nelsoni	4019135220	4019.14
3385	Alouatta macconnelli	4005971874	4005.97
3386	Piliocolobus semlikiensis	3994977519	3994.98
3387	Salinomys delicatus	3993229552	3993.23
3388	Leontocebus leucogenys	3992136793	3992.14
3389	Rhinolophus hillorum	3975220067	3975.22
3390	Diplomys labilis	3968370098	3968.37
3391	Phloeomys pallidus	3960599494	3960.60
3392	Lemur catta	3956032838	3956.03
3393	Mazama bricenii	3954552712	3954.55
3394	Microgale parvula	3937920477	3937.92
3395	Microgale cowani	3936706747	3936.71
3396	Microgale talazaci	3936151974	3936.15
3397	Echinops telfairi	3935873285	3935.87
3398	Lestodelphys halli	3935783384	3935.78
3399	Semnopithecus ajax	3930810370	3930.81
3400	Hylaeamys perenensis	3892559270	3892.56

3401	Lepus alleni	3872897359	3872.90
3402	Brachytarsomys albicauda	3848833279	3848.83
3403	Punomys lemminus	3847891979	3847.89
3404	Dorcatragus megalotis	3837431107	3837.43
3405	Hipposideros macrobullatus	3836512393	3836.51
3406	Macaca ochreata	3832994684	3832.99
3407	Thalpomys cerradensis	3821459980	3821.46
3408	Cebus cesarae	3815987373	3815.99
3409	Neodon fuscus	3808022113	3808.02
3410	Akodon polopi	3789442480	3789.44
3411	Eumops maurus	3760962114	3760.96
3412	Bathyergus suillus	3753205859	3753.21
3413	Chaerephon atsinanana	3749862705	3749.86
3414	Capra falconeri	3742701133	3742.70
3415	Sorex macrodon	3732578702	3732.58
3416	Sturnira bakeri	3721278074	3721.28
3417	Micronycteris sanborni	3720765415	3720.77
3418	Niviventer rapit	3718774458	3718.77
3419	Cryptotis gracilis	3718472877	3718.47
3420	Aotus brumbacki	3713342636	3713.34
3421	Chlamyphorus truncatus	3708624702	3708.62
3422	Ctenomys johannis	3708364370	3708.36
3423	Daubentonia madagascariensis	3706012406	3706.01
3424	Sylvisorex howelli	3683181361	3683.18
3425	Ammodorcas clarkei	3682847054	3682.85
3426	Chrysochloris asiatica	3676711331	3676.71
3427	Grammomys aridulus	3656127453	3656.13
3428	Akodon siberiae	3655091590	3655.09
3429	Paraxerus lucifer	3646354312	3646.35
3430	Xeronycteris vieirai	3635859544	3635.86
3431	Nyctalus furvus	3603121757	3603.12
3432	Dipodomys californicus	3602380710	3602.38
3433	Hipposideros calcaratus	3592416438	3592.42
3434	Gorilla beringei	3582565735	3582.57
3435	Dipodomys panamintinus	3579182078	3579.18
3436	Alouatta sara	3569136514	3569.14
3437	Coendou melanurus	3564167316	3564.17
3438	Kerivoula crypta	3558472444	3558.47
3439	Sminthopsis leucopus	3553828429	3553.83
3440	Presbytis bicolor	3552264853	3552.26
3441	Petrogale penicillata	3549396622	3549.40

3442	Alticola montosa	3547866182	3547.87
3443	Callicebus melanochir	3547844353	3547.84
3444	Aepeomys lugens	3537850002	3537.85
3445	Brachyuromys ramirohitra	3532500160	3532.50
3446	Mazama pandora	3526771782	3526.77
3447	Rhinolophus madurensis	3504928795	3504.93
3448	Canis simensis	3490003434	3490.00
3449	Handleyomys rhabdops	3488220040	3488.22
3450	Ctenomys torquatus	3483357280	3483.36
3451	Euroscaptor mizura	3477265146	3477.27
3452	Alticola tuvinicus	3477187293	3477.19
3453	Sylvilagus dicei	3475518032	3475.52
3454	Hipposideros maggietaylorae	3475132431	3475.13
3455	Proechimys trinitatis	3466780095	3466.78
3456	Peromyscus spicilegus	3460413527	3460.41
3457	Grammomys caniceps	3460364823	3460.36
3458	Eliurus majori	3433508241	3433.51
3459	Murina bicolor	3431397105	3431.40
3460	Trichosurus cunninghami	3426920135	3426.92
3461	Eothenomys cachinus	3418744667	3418.74
3462	Cratogeomys merriami	3417569054	3417.57
3463	Coendou nycthemera	3415893319	3415.89
3464	Xerus princeps	3393520979	3393.52
3465	Sundasciurus samarensis	3378267417	3378.27
3466	Peromyscus megalops	3374595853	3374.60
3467	Crocidura aleksandrisi	3374566795	3374.57
3468	Miopithecus ogouensis	3367668829	3367.67
3469	Melomys lutillus	3359838680	3359.84
3470	Alouatta ululata	3352838670	3352.84
3471	Crocidura vosmaeri	3347613119	3347.61
3472	Nycticebus bancanus	3347481964	3347.48
3473	Tupaia discolor	3347481964	3347.48
3474	Ctenomys osvaldoreigi	3343797866	3343.80
3475	Amblysomus corriae	3342577145	3342.58
3476	Pithecia milleri	3341971532	3341.97
3477	Miniopterus mahafaliensis	3335667086	3335.67
3478	Otomys thomasi	3334333711	3334.33
3479	Myotis secundus	3329557564	3329.56
3480	Rhinopithecus avunculus	3320040208	3320.04
3481	Sicista pseudonapaea	3318497604	3318.50
3482	Calomys fecundus	3312072512	3312.07

3483	Lonchophylla handleyi	3274019687	3274.02
3484	Syntheosciurus brochus	3273758485	3273.76
3485	Myotis keenii	3266537109	3266.54
3486	Myotis nesopolus	3265306332	3265.31
3487	Echiothrix leucura	3265125245	3265.13
3488	Chodsigoa lamula	3260154194	3260.15
3489	Millardia kathleenae	3256746696	3256.75
3490	Rhipidomys venustus	3255177779	3255.18
3491	Neoromicia flavescens	3250816151	3250.82
3492	Phyllotis anitae	3248592551	3248.59
3493	Akodon molinae	3246702127	3246.70
3494	Abrawayaomys ruschii	3236567333	3236.57
3495	Maxomys ochraceiventer	3235389006	3235.39
3496	Otomys yaldeni	3235284403	3235.28
3497	Rhipidomys tribei	3233374654	3233.37
3498	Muntiacus feae	3224271043	3224.27
3499	Molossus aztecus	3221348391	3221.35
3500	Heteromys adspersus	3211883227	3211.88
3501	Bunomys fratrorum	3209798621	3209.80
3502	Otonyctomys hatti	3197984265	3197.98
3503	Monodelphis brevicaudata	3197580902	3197.58
3504	Thomasomys hudsoni	3196890072	3196.89
3505	Euryoryzomys legatus	3154334763	3154.33
3506	Sorex bairdi	3144062332	3144.06
3507	Neotoma goldmani	3137558830	3137.56
3508	Nyctophilus microtis	3136372585	3136.37
3509	Crocidura usambarae	3135982424	3135.98
3510	Hylomyscus walterverheyeni	3135488739	3135.49
3511	Cryptotis goldmani	3131689308	3131.69
3512	Proechimys guyannensis	3131449961	3131.45
3513	Necromys obscurus	3122852190	3122.85
3514	Crocidura latona	3115665845	3115.67
3515	Crocidura xantippe	3109394452	3109.39
3516	Thylamys macrurus	3106630298	3106.63
3517	Blarinella quadraticauda	3099169324	3099.17
3518	Peropteryx trinitatis	3097607902	3097.61
3519	Proechimys goeldii	3096869401	3096.87
3520	Thomasomys ischyrus	3096509078	3096.51
3521	Prosciurillus topapuensis	3093898952	3093.90
3522	Reithrodontomys darienensis	3087549919	3087.55
3523	Sorex cansulus	3082019307	3082.02

3524	Desmomys yaldeni	3073748365	3073.75
3525	Scotophilus marovaza	3064698211	3064.70
3526	Crocidura lanosa	3064593587	3064.59
3527	Sorex buchariensis	3062169835	3062.17
3528	Spalax graecus	3060600225	3060.60
3529	Dipodomys microps	3057319722	3057.32
3530	Phyllomys lundi	3042466905	3042.47
3531	Cryptotis mayensis	3041426605	3041.43
3532	Sorex planiceps	3039450636	3039.45
3533	Reithrodontomys creper	3039049701	3039.05
3534	Praomys mutoni	3036593462	3036.59
3535	Elephantulus rupestris	3026920322	3026.92
3536	Neamblysomus julianae	3025094592	3025.09
3537	Ctenomys talarum	3002014637	3002.01
3538	Pithecia pithecia	2998439728	2998.44
3539	Nomascus nasutus	2997133974	2997.13
3540	Okapia johnstoni	2993677036	2993.68
3541	Calomyscus hotsoni	2986247228	2986.25
3542	Cebus unicolor	2976392174	2976.39
3543	Pappogeomys bulleri	2975329912	2975.33
3544	Rhogeessa aeneus	2971687898	2971.69
3545	Hylomys parvus	2965163766	2965.16
3546	Microcebus murinus	2961539705	2961.54
3547	Microhydromys richardsoni	2949135264	2949.14
3548	Proechimys steerei	2944398206	2944.40
3549	Praomys misonnei	2938889608	2938.89
3550	Murina recondita	2938855105	2938.86
3551	Leontopithecus chrysopygus	2937300511	2937.30
3552	Paragalago cocos	2932991381	2932.99
3553	Sturnira sorianoi	2926146166	2926.15
3554	Lonchorhina inusitata	2910085381	2910.09
3555	Macaca hecki	2899354765	2899.35
3556	Eupleres goudotii	2875267860	2875.27
3557	Ctenomys validus	2872898673	2872.90
3558	Macaca nigra	2863332900	2863.33
3559	Spilocuscus rufoniger	2859591120	2859.59
3560	Peromyscus zarhynchus	2859099008	2859.10
3561	Akodon torques	2858533330	2858.53
3562	Bradypus tridactylus	2854542403	2854.54
3563	Calomys venustus	2838294809	2838.29
3564	Octomys mimax	2834847141	2834.85

3565	Galidia elegans	2830294727	2830.29
3566	Piliocolobus tholloni	2812403755	2812.40
3567	Apomys littoralis	2811341286	2811.34
3568	Tupaia montana	2809748661	2809.75
3569	Fossa fossana	2807147070	2807.15
3570	Callistomys pictus	2806724615	2806.72
3571	Scolomys melanops	2795957967	2795.96
3572	Rhinolophus mabuensis	2792016779	2792.02
3573	Chiropodomys muroides	2789217047	2789.22
3574	Tympanoctomys barrerae	2783552136	2783.55
3575	Amblysomus septentrionalis	2770974612	2770.97
3576	Eothenomys chinensis	2770042599	2770.04
3577	Abrawayaomys chebezi	2769229213	2769.23
3578	Fukomys anselli	2740096069	2740.10
3579	Megadendromus nikolausi	2728299323	2728.30
3580	Ctenomys colburni	2725731764	2725.73
3581	Mus sorella	2706706430	2706.71
3582	Crocidura sicula	2705453056	2705.45
3583	Macroscelides proboscideus	2702035534	2702.04
3584	Rattus arrogans	2694303506	2694.30
3585	Carpitalpa arendsi	2689820261	2689.82
3586	Peromyscus mekisturus	2678035629	2678.04
3587	Tarsius dentatus	2677676284	2677.68
3588	Nephelomys devius	2676747351	2676.75
3589	Thamnomys kempi	2669349843	2669.35
3590	Otomys fortior	2665660391	2665.66
3591	Cephalophus callipygus	2663917588	2663.92
3592	Colobus caudatus	2663763391	2663.76
3593	Emballonura beccarii	2662712894	2662.71
3594	Thomomys bulbivorus	2661219246	2661.22
3595	Microcebus griseorufus	2652365152	2652.37
3596	Gerbillus hoogstraali	2630426441	2630.43
3597	Marmosops ocellatus	2629825347	2629.83
3598	Peromyscus yucatanicus	2629212216	2629.21
3599	Parotomys brantsii	2627228594	2627.23
3600	Sorex rohweri	2618913618	2618.91
3601	Artibeus schwartzi	2604048339	2604.05
3602	Lophuromys rahmi	2601047963	2601.05
3603	Antechinus subtropicus	2596432260	2596.43
3604	Echymipera clara	2596035557	2596.04
3605	Aepeomys reigi	2582528831	2582.53

3606	Cryptotis obscura	2576698941	2576.70
3607	Musonycteris harrisoni	2576197060	2576.20
3608	Aotus nigriceps	2573161165	2573.16
3609	Nyctophilus major	2569902929	2569.90
3610	Mops petersoni	2569257545	2569.26
3611	Akodon sylvanus	2568837575	2568.84
3612	Rattus leucopus	2567214517	2567.21
3613	Crocidura tansaniana	2566873665	2566.87
3614	Neotamias speciosus	2566395066	2566.40
3615	Tarsipes rostratus	2561625380	2561.63
3616	Cebus trinitatis	2558522309	2558.52
3617	Lonchorhina orinocensis	2554081420	2554.08
3618	Craseonycteris thonglongyai	2548195474	2548.20
3619	Ctenomys lami	2542149230	2542.15
3620	Ochotona coreana	2540608762	2540.61
3621	Dorcopsis hageni	2535830444	2535.83
3622	Fukomys zechi	2525142051	2525.14
3623	Cratogeomys goldmani	2521545565	2521.55
3624	Notamacropus irma	2516444097	2516.44
3625	Peromyscus grandis	2514540951	2514.54
3626	Crocidura greenwoodi	2498188224	2498.19
3627	Trachypithecus geei	2497494761	2497.49
3628	Paragalago zanzibaricus	2489366428	2489.37
3629	Crocidura lucina	2485331111	2485.33
3630	Urocitellus mollis	2482905669	2482.91
3631	Echimys saturnus	2481822450	2481.82
3632	Tarsius supriatnai	2480433259	2480.43
3633	Thomasomys vestitus	2476228212	2476.23
3634	Dipodomys venustus	2474255459	2474.26
3635	Kerivoula muscina	2462381434	2462.38
3636	Taterillus lacustris	2461085343	2461.09
3637	Stenocephalemys ruppi	2450212545	2450.21
3638	Plecotus turkmenicus	2444131818	2444.13
3639	Lonchophylla mordax	2439659438	2439.66
3640	Sminthopsis griseoventer	2438496778	2438.50
3641	Cryptotis peruviensis	2437630909	2437.63
3642	Myzopoda schliemanni	2436660078	2436.66
3643	Eupleres major	2435965207	2435.97
3644	Urocitellus beldingi	2428566199	2428.57
3645	Neotamias rufus	2427906296	2427.91
3646	Callithrix geoffroyi	2423176080	2423.18

3647	Galidictis fasciata	2403446328	2403.45
3648	Bibimys torresi	2402491544	2402.49
3649	Micaelamys granti	2401346113	2401.35
3650	Eptesicus taddeii	2400935745	2400.94
3651	Hoolock leuconedys	2400543488	2400.54
3652	Haeromys pusillus	2397943511	2397.94
3653	Chrotomys whiteheadi	2396008411	2396.01
3654	Cryptomys pretoriae	2390309308	2390.31
3655	Phyllostomus latifolius	2387433696	2387.43
3656	Chaerephon jobensis	2377566317	2377.57
3657	Allochrocebus preussi	2377488081	2377.49
3658	Leontocebus lagonotus	2373046385	2373.05
3659	Eptesicus dimissus	2359478577	2359.48
3660	Sciurus alleni	2356299077	2356.30
3661	Acomys minous	2354408921	2354.41
3662	Callosciurus baluensis	2353282077	2353.28
3663	Oryzorictes tetradactylus	2345457361	2345.46
3664	Peromyscus merriami	2337605348	2337.61
3665	Calomyscus grandis	2318357275	2318.36
3666	Ctenomys fulvus	2315381502	2315.38
3667	Dendrolagus inustus	2313105473	2313.11
3668	Oligoryzomys vegetus	2309574599	2309.57
3669	Tragelaphus buxtoni	2306661263	2306.66
3670	Exilisciurus whiteheadi	2295208529	2295.21
3671	Episoriculus fumidus	2292793214	2292.79
3672	Crocidura zaitsevi	2291791172	2291.79
3673	Ozimops beccarii	2288937790	2288.94
3674	Dasyprocta kalinowskii	2271854999	2271.85
3675	Thomasomys rosalinda	2263525100	2263.53
3676	Choeroniscus periosus	2256593120	2256.59
3677	Microsciurus santanderensis	2245256559	2245.26
3678	Oecomys phaeotis	2239823078	2239.82
3679	Capricornis rubidus	2211858130	2211.86
3680	Presbytis canicrus	2203113390	2203.11
3681	Proedromys bedfordi	2199582679	2199.58
3682	Petromyscus collinus	2198232030	2198.23
3683	Cheracebus lugens	2196175579	2196.18
3684	Thylamys citellus	2192718047	2192.72
3685	Marmosops parvidens	2191971614	2191.97
3686	Ctenomys mendocinus	2184503105	2184.50
3687	Plecturocebus oenanthe	2178721144	2178.72

3688	Saguinus geoffroyi	2172120182	2172.12
3689	Eupetaurus cinereus	2171609643	2171.61
3690	Rattus novaeguineae	2168252863	2168.25
3691	Cavia patzelti	2146017555	2146.02
3692	Pithecia inusta	2136053274	2136.05
3693	Rhogeessa hussoni	2133920153	2133.92
3694	Akodon orophilus	2129140808	2129.14
3695	Ctenomys steinbachi	2114852047	2114.85
3696	Crocidura nimbasilvanus	2112290963	2112.29
3697	Delanymys brooksi	2112037594	2112.04
3698	Micronycteris brosseti	2107585668	2107.59
3699	Pipistrellus raceyi	2102179119	2102.18
3700	Ctenomys yolandae	2085394212	2085.39
3701	Alionycteris paucidentata	2075980337	2075.98
3702	Crocidura caliginea	2073187546	2073.19
3703	Dasypus yepesi	2065015720	2065.02
3704	Thomasomys ladewi	2059097556	2059.10
3705	Notamacropus agilis	2056590620	2056.59
3706	Rhipidomys nitela	2055053635	2055.05
3707	Hypsugo vordermanni	2049587733	2049.59
3708	Ctenomys pearsoni	2038636488	2038.64
3709	Saimiri sciureus	2035526669	2035.53
3710	Feroculus feroculus	2028434751	2028.43
3711	Ailuropoda melanoleuca	2026818762	2026.82
3712	Arctocebus aureus	2000536117	2000.54
3713	Oxymycterus josei	1995431120	1995.43
3714	Sapajus robustus	1991550226	1991.55
3715	Geomys arenarius	1990750450	1990.75
3716	Apomys abrae	1988784654	1988.78
3717	Sicista caudata	1988267261	1988.27
3718	Mirzamys norahae	1986502244	1986.50
3719	Platyrrhinus masu	1982463183	1982.46
3720	Myoprocta acouchy	1977085413	1977.09
3721	Sturnira perla	1974906980	1974.91
3722	Rhipidomys ochrogaster	1973578854	1973.58
3723	Akodon budini	1966090356	1966.09
3724	Rhinolophus cohenae	1964267381	1964.27
3725	Kerivoula africana	1953885756	1953.89
3726	Ctenomys flamarioni	1951709835	1951.71
3727	Hylomyscus heinrichorum	1951370232	1951.37
3728	Mus callewaerti	1947581294	1947.58

3729	Gazella leptoceros	1947408995	1947.41
3730	Phalanger mimicus	1947082078	1947.08
3731	Rattus satarae	1944775774	1944.78
3732	Carollia manu	1941926313	1941.93
3733	Urocitellus canus	1938510349	1938.51
3734	Sylvisorex oriundus	1925926676	1925.93
3735	Mus crociduroides	1925814637	1925.81
3736	Capra cylindricornis	1918704951	1918.70
3737	Thomomys idahoensis	1917809911	1917.81
3738	Peromyscus keeni	1908377713	1908.38
3739	Petrogale herberti	1898014507	1898.01
3740	Wiedomys cerradensis	1897573312	1897.57
3741	Miniopterus griffithsi	1891813593	1891.81
3742	Chaetodipus spinatus	1890739036	1890.74
3743	Miniopterus aelleni	1890428162	1890.43
3744	Aconaemys fuscus	1889263364	1889.26
3745	Molossops aequatorianus	1888989140	1888.99
3746	Mandrillus sphinx	1875030737	1875.03
3747	Pithecia irrorata	1866641290	1866.64
3748	Hapalemur griseus	1863260264	1863.26
3749	Monticolomys koopmani	1857738967	1857.74
3750	Perognathus alticola	1855930207	1855.93
3751	Sylvilagus brasiliensis	1854848286	1854.85
3752	Dipodomys nelsoni	1854097729	1854.10
3753	Hipposideros durgadasi	1843246866	1843.25
3754	Peromyscus perfulvus	1842080084	1842.08
3755	Sciurocheirus gabonensis	1840102649	1840.10
3756	Oreamnos americanus	1833409764	1833.41
3757	Praomys lukolelae	1821946876	1821.95
3758	Lagidium ahuacaense	1818664843	1818.66
3759	Oxymycterus hiska	1811005762	1811.01
3760	Neotoma stephensi	1810899988	1810.90
3761	Oecomys rutilus	1809783806	1809.78
3762	Dorcopsis muelleri	1808601445	1808.60
3763	Pattonomys occasius	1807744698	1807.74
3764	Heimyscus fumosus	1806013610	1806.01
3765	Lariscus hosei	1803930146	1803.93
3766	Gerbillus stigmonyx	1799242297	1799.24
3767	Plagiodontia aedium	1793337907	1793.34
3768	Neacomys paracou	1792524335	1792.52
3769	Eulemur fulvus	1780018810	1780.02

3770	Ctenomys leucodon	1774910989	1774.91
3771	Necromys temchuki	1774465873	1774.47
3772	Sorex hosonoi	1769697976	1769.70
3773	Plecturocebus moloch	1761711344	1761.71
3774	Thomasomys macrotis	1754401219	1754.40
3775	Gerbillus rosalinda	1749584479	1749.58
3776	Apomys datae	1748418115	1748.42
3777	Paratriaenops furculus	1740931953	1740.93
3778	Volemys millicens	1736969991	1736.97
3779	Marmosops invictus	1735706346	1735.71
3780	Gracilinanus aceramarcae	1729960593	1729.96
3781	Ctenomys maulinus	1728670287	1728.67
3782	Geomys attwateri	1724787022	1724.79
3783	Praomys petteri	1710130606	1710.13
3784	Alouatta puruensis	1699448657	1699.45
3785	Rattus sordidus	1695690256	1695.69
3786	Neotamias ruficaudus	1694448930	1694.45
3787	Nephelomys caracolus	1694177397	1694.18
3788	Toromys rhipidurus	1693774499	1693.77
3789	Macaca brunnescens	1689745226	1689.75
3790	Cebuella niveiventris	1684434040	1684.43
3791	Rhynchocyon chrysopygus	1678026553	1678.03
3792	Lophuromys luteogaster	1674632947	1674.63
3793	Proedromys liangshanensis	1668028300	1668.03
3794	Cabassous chacoensis	1664803289	1664.80
3795	Jaculus thaleri	1662987206	1662.99
3796	Equus zebra	1655486119	1655.49
3797	Notocitellus annulatus	1650447337	1650.45
3798	Solenodon paradoxus	1646061513	1646.06
3799	Gerbillus mackilligini	1643911593	1643.91
3800	Oecomys rex	1641205863	1641.21
3801	Erythrocebus baumstarki	1640515084	1640.52
3802	Arabitragus jayakari	1638588245	1638.59
3803	Lemmus amurensis	1637952873	1637.95
3804	Colobus satanas	1631031069	1631.03
3805	Dremomys everetti	1628985707	1628.99
3806	Rattus osgoodi	1623050046	1623.05
3807	Glyphotes simus	1620279656	1620.28
3808	Praomys hartwigi	1616177943	1616.18
3809	Akodon toba	1615925717	1615.93
3810	Neotamias sonomae	1606440686	1606.44

3811	Lestoros inca	1602695507	1602.70
3812	Pseudomys novaehollandiae	1600091090	1600.09
3813	Piliocolobus parmentieri	1587968260	1587.97
3814	Vampyriscus brocki	1585889796	1585.89
3815	Dendrogale melanura	1585719187	1585.72
3816	Chaetodipus pernix	1584230156	1584.23
3817	Leptomys elegans	1577206717	1577.21
3818	Olallamys edax	1573672027	1573.67
3819	Eliurus grandidieri	1570992201	1570.99
3820	Lenoxus apicalis	1563080571	1563.08
3821	Toromys grandis	1557459620	1557.46
3822	Phyllomys mantiqueirensis	1555970220	1555.97
3823	Saguinus midas	1554474952	1554.47
3824	Spalax giganteus	1551527468	1551.53
3825	Trinomys paratus	1545465627	1545.47
3826	Juscelinomys candango	1541163122	1541.16
3827	Vandeleuria nilagirica	1539984374	1539.98
3828	Acomys nesiotes	1536322097	1536.32
3829	Mus cypriacus	1536322097	1536.32
3830	Thomomys townsendii	1535361481	1535.36
3831	Crocidura allex	1535304823	1535.30
3832	Notocitellus adocetus	1530639496	1530.64
3833	Leptomys ernstmayri	1527815892	1527.82
3834	Macaca nigrescens	1526550380	1526.55
3835	Cryptotis niausa	1524741101	1524.74
3836	Eudorcas tilonura	1518165948	1518.17
3837	Craseomys rex	1513854342	1513.85
3838	Apomys hylocetes	1509561354	1509.56
3839	Hadromys humei	1507303402	1507.30
3840	Rattus bontanus	1506485876	1506.49
3841	Capricornis swinhoei	1503046790	1503.05
3842	Scotophilus tandrefana	1499526195	1499.53
3843	Geogale aurita	1481076887	1481.08
3844	Saimiri oerstedii	1476680659	1476.68
3845	Ochotona forresti	1473373244	1473.37
3846	Neotoma devia	1470982829	1470.98
3847	Sciurus colliaei	1468832754	1468.83
3848	Thylogale thetis	1467138496	1467.14
3849	Lasiurus atratus	1465100164	1465.10
3850	Microakodontomys transitorius	1459290212	1459.29
3851	Balantiopteryx infusca	1457019795	1457.02

3852	Dasyprocta croconota	1447021601	1447.02
3853	Crunomys celebensis	1441842721	1441.84
3854	Melomys leucogaster	1440099329	1440.10
3855	Sorex maritimensis	1430740845	1430.74
3856	Paraxerus vincenti	1429820393	1429.82
3857	Cephalophus spadix	1419214869	1419.21
3858	Nelsonia neotomodon	1418976433	1418.98
3859	Sundasciurus jentinki	1415498693	1415.50
3860	Reithrodontomys burti	1413666678	1413.67
3861	Antechinus minimus	1413434215	1413.43
3862	Sciurus sanborni	1410261922	1410.26
3863	Crocidura manengubae	1409580494	1409.58
3864	Microtus tatricus	1400843424	1400.84
3865	Hipposideros crumeniferus	1392980560	1392.98
3866	Aethalops aequalis	1390005383	1390.01
3867	Lophostoma schulzi	1387131880	1387.13
3868	Microtus irani	1384490107	1384.49
3869	Taeromys hamatus	1383328103	1383.33
3870	Paramelomys naso	1381160754	1381.16
3871	Rhinolophus maclaudi	1380069228	1380.07
3872	Funambulus obscurus	1377380137	1377.38
3873	Bibimys chacoensis	1377074907	1377.07
3874	Pithecia mittermeieri	1375036480	1375.04
3875	Paramelomys moncktoni	1369358552	1369.36
3876	Thrichomys inermis	1367399042	1367.40
3877	Oxymycterus amazonicus	1357047925	1357.05
3878	Coendou ichillus	1356959420	1356.96
3879	Chiropotes chiropotes	1354637773	1354.64
3880	Chaetodipus artus	1351280165	1351.28
3881	Ctenomys dorbignyi	1348989780	1348.99
3882	Neamblysomus gunningi	1348441299	1348.44
3883	Thomasomys monochromos	1347928262	1347.93
3884	Euneomys fossor	1347583266	1347.58
3885	Leontocebus weddelli	1344388488	1344.39
3886	Microtus anatolicus	1339386292	1339.39
3887	Leopoldamys milleti	1337150512	1337.15
3888	Sminthopsis virginiae	1336136482	1336.14
3889	Thomasomys eleusis	1335280827	1335.28
3890	Mesocricetus auratus	1333137956	1333.14
3891	Myomimus roachi	1331901371	1331.90
3892	Saimiri ustus	1329465526	1329.47

3893	Plecturocebus ornatus	1324934040	1324.93
3894	Crocidura tarella	1323609610	1323.61
3895	Pan paniscus	1320937297	1320.94
3896	Molossus alvarezi	1317649132	1317.65
3897	Thylogale billardierii	1315858082	1315.86
3898	Sylvisorex camerunensis	1312676390	1312.68
3899	Ellobius alaicus	1291205073	1291.21
3900	Emballonura dianae	1290171964	1290.17
3901	Podogymnura truei	1288743155	1288.74
3902	Crocidura congobelgica	1283342839	1283.34
3903	Equus africanus	1281730842	1281.73
3904	Tryphomys adustus	1276063610	1276.06
3905	Eropeplus canus	1273914138	1273.91
3906	Dorcopsis luctuosa	1271706820	1271.71
3907	Parotomys littledalei	1270390718	1270.39
3908	Microtus transcaspicus	1268958986	1268.96
3909	Cercartetus lepidus	1267858713	1267.86
3910	Nyctophilus corbeni	1265474225	1265.47
3911	Sarcophilus harrisii	1252649442	1252.65
3912	Pseudomys higginsi	1252649442	1252.65
3913	Dasyurus viverrinus	1252647623	1252.65
3914	Thomasomys incanus	1252282270	1252.28
3915	Euryoryzomys emmonsae	1250932680	1250.93
3916	Nyctophilus sherrini	1247094413	1247.09
3917	Chlorotalpa duthieae	1246627894	1246.63
3918	Oecomys auyantepui	1243702297	1243.70
3919	Paraleptomys wilhelmina	1238189519	1238.19
3920	Callosciurus orestes	1235953452	1235.95
3921	Callithrix flaviceps	1229600807	1229.60
3922	Microdillus peeli	1224887241	1224.89
3923	Nilgiritragus hylocrius	1208653574	1208.65
3924	Akodon kofordi	1206707208	1206.71
3925	Crocidura grassei	1205846638	1205.85
3926	Marmosa xerophila	1204642094	1204.64
3927	Perameles gunnii	1203429329	1203.43
3928	Hipposideros coxi	1202805360	1202.81
3929	Macroderma gigas	1201924296	1201.92
3930	Lepilemur edwardsi	1197550765	1197.55
3931	Ctenomys pilarensis	1196674586	1196.67
3932	Nyctophilus holtorum	1193661216	1193.66
3933	Graphiurus monardi	1185096122	1185.10

3934	Heteromys nelsoni	1183552758	1183.55
3935	Sorex emarginatus	1181129541	1181.13
3936	Ateles paniscus	1175846860	1175.85
3937	Apodemus gurkha	1174938157	1174.94
3938	Microgale grandidieri	1173631411	1173.63
3939	Neotamias senex	1167685611	1167.69
3940	Chiropotes albinasus	1167444371	1167.44
3941	Tadarida insignis	1164365612	1164.37
3942	Eptesicus kobayashii	1162811022	1162.81
3943	Microtus schidlovskii	1160908043	1160.91
3944	Zenkerella insignis	1159603901	1159.60
3945	Mus setzeri	1153435817	1153.44
3946	Saccopteryx antioquensis	1151327019	1151.33
3947	Hipposideros papua	1150549985	1150.55
3948	Mormopterus doriae	1146214469	1146.21
3949	Chaetodipus rudinoris	1145580315	1145.58
3950	Maxomys inas	1139786544	1139.79
3951	Isothrix pagurus	1139096899	1139.10
3952	Lonchophylla cadenai	1137252403	1137.25
3953	Phyllotis definitus	1136825078	1136.83
3954	Plecotus sardus	1134900983	1134.90
3955	Uropsilus andersoni	1134614269	1134.61
3956	Nelsonia goldmani	1133156138	1133.16
3957	Presbytis chrysomelas	1122425148	1122.43
3958	Aotus trivirgatus	1121180620	1121.18
3959	Marmosops juninensis	1120203862	1120.20
3960	Hipposideros orbiculus	1113238375	1113.24
3961	Nephelomys auriventer	1109237328	1109.24
3962	Harpiola isodon	1104914612	1104.91
3963	Ozotoceros bezoarticus	1104496234	1104.50
3964	Proechimys gardneri	1101346667	1101.35
3965	Eulemur rufus	1100649255	1100.65
3966	Piliocolobus langi	1091581941	1091.58
3967	Dyacopterus rickarti	1091226696	1091.23
3968	Thylogale brunii	1090143560	1090.14
3969	Crocidura susiana	1083834607	1083.83
3970	Eothenomys wardi	1083770981	1083.77
3971	Sorex arizonae	1078621086	1078.62
3972	Orthogeomys heterodus	1075925100	1075.93
3973	Neopteryx frosti	1074633059	1074.63
3974	Calomys callidus	1072651065	1072.65

3975	Trinomys eliasi	1072319104	1072.32
3976	Geomys knoxjonesi	1070904539	1070.90
3977	Hadromys yunnanensis	1065577648	1065.58
3978	Xerospermophilus mohavensis	1063387368	1063.39
3979	Hypsugo kitcheneri	1063189820	1063.19
3980	Surdisorex norae	1059890476	1059.89
3981	Akodon sanctipaulensis	1056822921	1056.82
3982	Aotus miconax	1054637945	1054.64
3983	Reithrodontomys rodriguezi	1054582698	1054.58
3984	Sorex milleri	1053193090	1053.19
3985	Hipposideros griffini	1049822598	1049.82
3986	Pseudomys patrius	1048654425	1048.65
3987	Tarsomys apoensis	1047750959	1047.75
3988	Carpomys melanurus	1042451002	1042.45
3989	Leontopithecus rosalia	1041047860	1041.05
3990	Mammelomys rattoides	1039603049	1039.60
3991	Saguinus niger	1034514752	1034.51
3992	Cheirogaleus crossleyi	1033331068	1033.33
3993	Taeromys taerae	1024462595	1024.46
3994	Crocidura ultima	1019311086	1019.31
3995	Miniopterus egeri	1018706080	1018.71
3996	Microcebus ravelobensis	1016290110	1016.29
3997	Melanomys robustulus	1015304676	1015.30
3998	Margaretamys parvus	1012784666	1012.78
3999	Notiosorex cockrumi	1009997185	1010.00
4000	Mus mayori	1009046134	1009.05
4001	Otomys occidentalis	1008723416	1008.72
4002	Thomomys clusius	1008171904	1008.17
4003	Melasmothrix naso	1006425792	1006.43
4004	Neoromicia isabella	1003847613	1003.85
4005	Notamacropus parma	1003154889	1003.15
4006	Rattus mordax	1001509780	1001.51
4007	Praomys obscurus	1000838582	1000.84
4008	Paraxerus cooperi	999730393.5	999.73
4009	Margaretamys elegans	999213786.3	999.21
4010	Phalanger intercastellanus	997221274.4	997.22
4011	Piliocolobus epieni	993832403.6	993.83
4012	Pithecia aequatorialis	992028408.7	992.03
4013	Rattus richardsoni	988068663.2	988.07
4014	Melomys frigicola	984771803.3	984.77
4015	Murina harrisoni	979977470.8	979.98

4016	Hypsugo arabicus	978488762.6	978.49
4017	Eudorcas albonotata	972580650	972.58
4018	Gyldenstolpia fronto	970096142.8	970.10
4019	Geoxus annectens	959780367.9	959.78
4020	Callithrix kuhlii	957539255.9	957.54
4021	Taphozous troughtoni	955754850.6	955.75
4022	Ctenomys fochi	951084896.4	951.08
4023	Zygogeomys trichopus	949628768.5	949.63
4024	Lagothrix flavicauda	949625223.4	949.63
4025	Microtus kermanensis	949174008.7	949.17
4026	Thomomys monticola	946266586.1	946.27
4027	Ochotona hoffmanni	942702901.8	942.70
4028	Cratogeomys perotensis	938914413.1	938.91
4029	Microgale taiva	938031798.6	938.03
4030	Cryptonanus guahybae	937827979.5	937.83
4031	Eulemur rubriventer	932814589.2	932.81
4032	Cryptotis tamensis	925430424.7	925.43
4033	Macrotarsomys ingens	921136533.3	921.14
4034	Nycteris madagascariensis	916097382.3	916.10
4035	Scleronycteris ega	914483649.9	914.48
4036	Phaner pallescens	909543611.6	909.54
4037	Neotoma phenax	908297268.8	908.30
4038	Alticola albicaudus	906393513.2	906.39
4039	Eulemur mongoz	903756733.2	903.76
4040	Myosorex okuensis	899881964.3	899.88
4041	Lophuromys huttereri	898495877.2	898.50
4042	Gerbillus rupicola	897626785	897.63
4043	Crocidura pitmani	896754604.8	896.75
4044	Microcebus myoxinus	891564062.5	891.56
4045	Paramelomys lorentzii	890123247.9	890.12
4046	Crocidura stenocephala	885449897.3	885.45
4047	Eptesicus tatei	885089764.3	885.09
4048	Cryptotis mam	882890620.9	882.89
4049	Chiropotes utahickae	871631611.1	871.63
4050	Bettongia gaimardi	869322682.1	869.32
4051	Crocidura picea	867398930.4	867.40
4052	Rhinolophus proconsulis	864682402.3	864.68
4053	Ctenomys peruanus	864561322.4	864.56
4054	Praomys minor	864560252.4	864.56
4055	Avahi occidentalis	861697221.7	861.70
4056	Phyllotis magister	861121021.8	861.12

4057	Myotis lavali	858030353.8	858.03
4058	Anourosorex schmidi	857589258.7	857.59
4059	Neotamias siskiyou	857204381.2	857.20
4060	Propithecus coronatus	855350627.2	855.35
4061	Neohylomys hainanensis	854592171.8	854.59
4062	Arborimus pomo	854388150.3	854.39
4063	Avahi laniger	852580475.8	852.58
4064	Allactaga firouzi	851952271.3	851.95
4065	Crocidura absconditus	851771948	851.77
4066	Dipodomys compactus	848404215.3	848.40
4067	Cratogeomys planiceps	846214879.9	846.21
4068	Rattus giluwensis	843966124.5	843.97
4069	Murina gracilis	840909281	840.91
4070	Rhinolophus maendeleo	839412339.6	839.41
4071	Thrichomys pachyurus	837126777.7	837.13
4072	Thylamys fenestrae	835117267.7	835.12
4073	Pipistrellus wattsi	834864935.1	834.86
4074	Plecturocebus toppini	831538741.8	831.54
4075	Microdipodops megacephalus	824768835.7	824.77
4076	Thomasomys gracilis	819917536.8	819.92
4077	Pteropus chrysoproctus	814505528.9	814.51
4078	Neotamias durangae	812620859.1	812.62
4079	Tachyoryctes macrocephalus	811242175.3	811.24
4080	Chiruromys vates	810091893.5	810.09
4081	Myosorex tenuis	807349933.3	807.35
4082	Diplogale hosei	804691452.1	804.69
4083	Orthogeomys underwoodi	803576419.9	803.58
4084	Lonchothrix emiliae	803264555.1	803.26
4085	Crocidura sapaensis	799722992.6	799.72
4086	Mus neavei	799122936.7	799.12
4087	Harpiola grisea	794105312	794.11
4088	Petromus typicus	789914346.2	789.91
4089	Crocidura wuchihensis	788572078.9	788.57
4090	Crocidura newmarki	780957800.7	780.96
4091	Oligoryzomys moojeni	780858434.3	780.86
4092	Neotamias quadrimaculatus	780705353.9	780.71
4093	Plecotus balensis	777837926.6	777.84
4094	Peroryctes broadbenti	776225711.7	776.23
4095	Chaetodipus lineatus	775923949.8	775.92
4096	Ctenomys australis	773366911	773.37
4097	Crocidura kivuana	758538590	758.54

4098	Dipodomys nitratoides	758205852.3	758.21
4099	Cheracebus lucifer	752045944.5	752.05
4100	Zyzomys argurus	750178759	750.18
4101	Planigale novaeguineae	748316372.8	748.32
4102	Hipposideros scutinares	742345803.5	742.35
4103	Mogera etigo	738288417.5	738.29
4104	Scotinomys xerampelinus	736074182.1	736.07
4105	Dryomys laniger	732990107.9	732.99
4106	Batomys granti	727268370	727.27
4107	Dasyurus geoffroii	725007640.4	725.01
4108	Galagoides kumbirensis	724879709.1	724.88
4109	Gerbillus bottai	724656087.3	724.66
4110	Sylvilagus tapetillus	723616676.9	723.62
4111	Myosorex longicaudatus	721000984.1	721.00
4112	Ateles marginatus	719835889.1	719.84
4113	Graomys edithae	719115541	719.12
4114	Chaetodipus goldmani	713676423.6	713.68
4115	Eligmodontia moreni	708202587.7	708.20
4116	Propithecus coquereli	705057494.8	705.06
4117	Taeromys arcuatus	704809903.9	704.81
4118	Juliomys rimofrons	703994260.5	703.99
4119	Dasyurus hallucatus	703921477.6	703.92
4120	Chiropotes sagulatus	700754691.4	700.75
4121	Plecturocebus brunneus	699430100.7	699.43
4122	Euroscaptor parvidens	698127917.7	698.13
4123	Micronycteris yatesi	696219433	696.22
4124	Cephalophus jentinki	694486266.9	694.49
4125	Cephalophus zebra	694486266.9	694.49
4126	Marmosops neblina	693781913.2	693.78
4127	Xerospermophilus perotensis	691329826.7	691.33
4128	Pongo abelii	689199747.4	689.20
4129	Lepilemur ruficaudatus	685397365.9	685.40
4130	Aconaemys porteri	684323571.6	684.32
4131	Srilankamys ohiensis	681911040.7	681.91
4132	Dactylomys boliviensis	679634200.2	679.63
4133	Crocidura gracilipes	677451295.2	677.45
4134	Crocidura mutesae	677283922.6	677.28
4135	Ctenomys lewisi	669430583.9	669.43
4136	Dobsonia moluccensis	666428340.8	666.43
4137	Microgale thomasi	665201705.4	665.20
4138	Ozimops kitcheneri	662951651.9	662.95

4139	Crocidura telfordi	662803137	662.80
4140	Taterillus arenarius	662659495.3	662.66
4141	Pseudochirulus forbesi	662658526.2	662.66
4142	Ctenomys sericeus	660855876.2	660.86
4143	Glischropus javanus	660829367.8	660.83
4144	Saccopteryx gymnura	659911677.3	659.91
4145	Akodon mystax	657593218	657.59
4146	Mus oubanguii	657449037.6	657.45
4147	Gerbillus pulvinatus	656434022.8	656.43
4148	Geomys personatus	652906620.2	652.91
4149	Zaglossus bruijnii	650763525	650.76
4150	Crocidura orientalis	649355558.3	649.36
4151	Cephalophus adersi	648600311.5	648.60
4152	Sciurus arizonensis	648374359.3	648.37
4153	Piliocolobus kirkii	647921163	647.92
4154	Ozimops loriae	646645382	646.65
4155	Rupicapra pyrenaica	645095232.1	645.10
4156	Hipposideros semoni	644429352	644.43
4157	Myotis annatessae	643683041.1	643.68
4158	Cryptonanus unduaviensis	636312005.2	636.31
4159	Rhabdomys intermedius	633417153.8	633.42
4160	Plecturocebus cupreus	627949570.6	627.95
4161	Osphranter rufus	625644388.2	625.64
4162	Maxomys alticola	621719461.1	621.72
4163	Habromys delicatulus	619763016	619.76
4164	Ochotona turuchanensis	617994926.4	617.99
4165	Tragulus versicolor	612383937.7	612.38
4166	Cebus malitiosus	611510023	611.51
4167	Hapalomys longicaudatus	610882988.5	610.88
4168	Graphiurus surdus	609890322.9	609.89
4169	Lagorchestes conspicillatus	609799922.7	609.80
4170	Bunomys penitus	607586768.2	607.59
4171	Marmota menzbieri	607348640.1	607.35
4172	Camelus ferus	607091577.6	607.09
4173	Microtus dogramacii	600083835.3	600.08
4174	Ctenomys knighti	598199974.5	598.20
4175	Paulamys naso	598023904.5	598.02
4176	Rattus hainaldi	598023904.5	598.02
4177	Otomys uzungwensis	597886617	597.89
4178	Rattus jobiensis	593442738.9	593.44
4179	Mesembriomys gouldii	592397658.8	592.40

4180	Limnomys sibuanus	589824092.5	589.82
4181	Chrotomys silaceus	589271789.8	589.27
4182	Syconycteris carolinae	585024225.4	585.02
4183	Pteropus caniceps	585024225.4	585.02
4184	Pteropus personatus	585024225.4	585.02
4185	Rattus morotaiensis	585024225.4	585.02
4186	Phalanger ornatus	585023017.4	585.02
4187	Cryptotis magna	583684374.3	583.68
4188	Bison bonasus	576674723.3	576.67
4189	Crateromys schadenbergi	575196075.3	575.20
4190	Neotamias cinereicollis	573630825.7	573.63
4191	Hybomys eisentrauti	569387698.7	569.39
4192	Carpomys phaeurus	568424656.2	568.42
4193	Rhynchomys soricoides	568424656.2	568.42
4194	Dobsonia viridis	568115042.3	568.12
4195	Alouatta discolor	567706464.7	567.71
4196	Cheirogaleus major	565074601.1	565.07
4197	Eulemur rufifrons	565061477.7	565.06
4198	Dobsonia beauforti	564398509.5	564.40
4199	Dobsonia emersa	563814642	563.81
4200	Spilocuscus wilsoni	563807800.4	563.81
4201	Petaurus biacensis	563807082.4	563.81
4202	Uromys boeadii	563807050.2	563.81
4203	Nomascus annamensis	563147791.5	563.15
4204	Steatomys jacksoni	558323759.1	558.32
4205	Prometheomys schaposchnikowi	556771213.4	556.77
4206	Brucepattersonius paradisus	554029284.2	554.03
4207	Neacomys guianae	551442459	551.44
4208	Didelphis imperfecta	550557504.4	550.56
4209	Sicista kluchorica	548897419	548.90
4210	Plecturocebus donacophilus	547916143.4	547.92
4211	Trachypithecus hatinhensis	544082720.4	544.08
4212	Caluromysiops irrupta	537776017.2	537.78
4213	Crocidura fischeri	536995359.5	537.00
4214	Pteropus temminckii	535607522.2	535.61
4215	Pteropus ocularis	535583710.8	535.58
4216	Pteropus speciosus	534019065.1	534.02
4217	Mus famulus	533812920.1	533.81
4218	Dendrolagus dorianus	532475155.1	532.48
4219	Gerbillus jamesi	530974795.4	530.97
4220	Marmosa quichua	529091938.9	529.09

4221	Fukomys hanangensis	529005212.1	529.01
4222	Urocricetus alticola	525301628.6	525.30
4223	Orthogeomys cavator	524537183.8	524.54
4224	Pattonomys flavidus	524252262.6	524.25
4225	Lophuromys pseudosikapusi	520557905	520.56
4226	Myosorex blarina	517403820	517.40
4227	Paracrocidura graueri	517186437.7	517.19
4228	Sciurus richmondi	517152385.2	517.15
4229	Meriones dahli	515161277.6	515.16
4230	Propithecus verreauxi	513010876.5	513.01
4231	Akodon surdus	508838151.3	508.84
4232	Microtus xanthognathus	506614095.1	506.61
4233	Pithecia chrysocephala	506344911.5	506.34
4234	Pteropus admiralitatum	503296373	503.30
4235	Nyctimene vizcaccia	503296372.9	503.30
4236	Crocidura ramona	502408574.4	502.41
4237	Phyllotis bonariensis	502149042	502.15
4238	Miniopterus fuscus	501503073.6	501.50
4239	Bos sauveli	501165587.6	501.17
4240	Pteropus dasymallus	500042278.3	500.04
4241	Nyctimene major	498344278.5	498.34
4242	Propithecus deckenii	498236153	498.24
4243	Kunsia tomentosus	496101229.6	496.10
4244	Rhinolophus xinanzhongguoensis	493148000.6	493.15
4245	Pithecia napensis	491015786.9	491.02
4246	Mico argentatus	490990927.8	490.99
4247	Microhydromys argenteus	489219330.4	489.22
4248	Crocidura batesi	488033592.3	488.03
4249	Coendou vestitus	487388323.5	487.39
4250	Gerbillus nigeriae	485031134.2	485.03
4251	Brucepattersonius igniventris	485019252.7	485.02
4252	Neotamias obscurus	481686697.9	481.69
4253	Plecturocebus dubius	480174756.8	480.17
4254	Glauconycteris superba	479129989.6	479.13
4255	Hipposideros wollastoni	478903494.4	478.90
4256	Sturnira burtonlimi	478849117.7	478.85
4257	Cratogeomys fulvescens	477265022.9	477.27
4258	Mirza coquereli	473845609.4	473.85
4259	Ctenomys boliviensis	470035674.4	470.04
4260	Procapra przewalskii	465740145.2	465.74
4261	Porcula salvania	465279254.3	465.28

4262	Thylamys tatei	464181507.9	464.18
4263	Gerbillurus vallinus	463768336.1	463.77
4264	Crocidura watasei	459659871.1	459.66
4265	Pteropus loochoensis	458235708.2	458.24
4266	Cistugo seabrae	456790350.5	456.79
4267	Diclidurus isabella	452086046.1	452.09
4268	Crocidura afeworkbekelei	451984643.1	451.98
4269	Hesperoptenus doriae	450870371.2	450.87
4270	Mico rondoni	450823358.5	450.82
4271	Bunolagus monticularis	450062917.6	450.06
4272	Saguinus bicolor	448198440.7	448.20
4273	Lonchophylla peracchii	444773410.9	444.77
4274	Dobsonia anderseni	443299650.7	443.30
4275	Pteropus capistratus	442320634.9	442.32
4276	Uromys neobritannicus	442320626.4	442.32
4277	Kerivoula myrella	442317983.4	442.32
4278	Melonycteris melanops	442317983.3	442.32
4279	Dobsonia praedatrix	442317983.1	442.32
4280	Taphozous australis	441749507	441.75
4281	Urocitellus townsendii	439789297	439.79
4282	Andalgalomys olrogi	439523121.7	439.52
4283	Chalinolobus picatus	439279633.4	439.28
4284	Hybomys lunaris	436587351.3	436.59
4285	Dendrolagus mbaiso	435019159	435.02
4286	Saguinus imperator	434479283.3	434.48
4287	Soricomys musseri	433984189.4	433.98
4288	Lepilemur otto	433871468.9	433.87
4289	Niviventer cameroni	432526204.9	432.53
4290	Diplomys caniceps	432217171.4	432.22
4291	Ozimops petersi	431258020.9	431.26
4292	Neotamias ochrogenys	429081200	429.08
4293	Calomyscus urartensis	425600241.6	425.60
4294	Poiana leightoni	425474050.5	425.47
4295	Paramelomys levipes	425187931.4	425.19
4296	Nesolagus timminsi	424739410.5	424.74
4297	Peromyscus simulus	418621960.7	418.62
4298	Ctenodactylus vali	418496137.4	418.50
4299	Cheirogaleus medius	416987768.9	416.99
4300	Spermophilus taurensis	409910408.6	409.91
4301	Pithecia monachus	407033198.4	407.03
4302	Cryptotis dinirensis	405778791	405.78

4303	Platyrrhinus aurarius	403189366.2	403.19
4304	Praomys morio	403127738	403.13
4305	Barbastella beijingensis	400836976.1	400.84
4306	Eumops floridanus	400315103.8	400.32
4307	Crocidura arabica	400298954.1	400.30
4308	Mastacomys fuscus	398309277.2	398.31
4309	Cheracebus medemi	397784007.9	397.78
4310	Mirzamys louiseae	397572960.9	397.57
4311	Hapalemur meridionalis	396963013.7	396.96
4312	Beatragus hunteri	395717347.1	395.72
4313	Dasyurus spartacus	393555517.1	393.56
4314	Myoictis wallacei	393547868.1	393.55
4315	Varecia variegata	393314872.3	393.31
4316	Saguinus mystax	392728656.2	392.73
4317	Mastomys shortridgei	392648736	392.65
4318	Pogonomelomys bruijnii	391017445.3	391.02
4319	Natalus primus	390808183.9	390.81
4320	Ochotona iliensis	389722728.2	389.72
4321	Brucepattersonius guarani	389385884.6	389.39
4322	Peromyscus sagax	389015348.4	389.02
4323	Solisorex pearsoni	386821558.1	386.82
4324	Prosciurillus weberi	386588047.6	386.59
4325	Piliocolobus tephrosceles	383736056.6	383.74
4326	Lemniscomys roseveari	381319328	381.32
4327	Hipposideros sorenseni	377662591.7	377.66
4328	Scotophilus livingstonii	377601204.3	377.60
4329	Suncus fellowesgordoni	375915711.9	375.92
4330	Pseudochirops albertisii	375642113.1	375.64
4331	Ctenomys famosus	374807976.7	374.81
4332	Miniopterus petersoni	373929865.9	373.93
4333	Brucepattersonius misionensis	371006026.7	371.01
4334	Saguinus labiatus	370315994	370.32
4335	Rusa alfredi	369376268.1	369.38
4336	Rhinolophus willardi	368967701.1	368.97
4337	Rhogeessa genowaysi	367426632.3	367.43
4338	Gerbillus occiduus	367054141.8	367.05
4339	Rhogeessa velilla	365333379.5	365.33
4340	Melogale cucphuongensis	362220809	362.22
4341	Abrothrix lanosus	359962140.1	359.96
4342	Sylvilagus cognatus	358872778	358.87
4343	Oryx leucoryx	358706194.2	358.71

4344	Chiruromys lamia	357836993.1	357.84
4345	Reithrodontomys hirsutus	357365352.6	357.37
4346	Funambulus layardi	356258377.3	356.26
4347	Cebus albifrons	356257948.2	356.26
4348	Hipposideros khaokhouayensis	355061802.5	355.06
4349	Neusticomys ferreirai	354986810.4	354.99
4350	Suncus hosei	353001468.8	353.00
4351	Notomys mitchellii	349330129	349.33
4352	Pteropus voeltzkowi	349187403.9	349.19
4353	Mops bakarii	349111224	349.11
4354	Crocidura yaldeni	348384372.4	348.38
4355	Crocidura phaeura	343180661.3	343.18
4356	Naemorhedus baileyi	343130360.1	343.13
4357	Pseudomys desertor	338639400.8	338.64
4358	Geocapromys brownii	337039572	337.04
4359	Trachypithecus leucocephalus	335573637.7	335.57
4360	Lepus castroviejoi	335272976.5	335.27
4361	Ctenomys fodax	334381062	334.38
4362	Eudiscopus denticulus	334129634.8	334.13
4363	Petromyscus monticularis	333205842.1	333.21
4364	Abditomys latidens	332499249.4	332.50
4365	Philander mcilhennyi	331853055.2	331.85
4366	Akodon reigi	330703714.9	330.70
4367	Ammospermophilus nelsoni	329508499.9	329.51
4368	Capra caucasica	328233905.9	328.23
4369	Dendromus oreas	327354848.5	327.35
4370	Trinomys moojeni	326237124.8	326.24
4371	Myosorex kihaulei	325876953.2	325.88
4372	Leggadina lakedownensis	324904918.9	324.90
4373	Heteromys oresterus	323562657.1	323.56
4374	Crocidura miya	323338630.1	323.34
4375	Arielulus cuprosus	322158785.9	322.16
4376	Suncus zeylanicus	317797575.4	317.80
4377	Planigale tenuirostris	314421556	314.42
4378	Hipposideros einnaythu	314333643.9	314.33
4379	Heteromys oasicus	312899167	312.90
4380	Eremitalpa granti	312898837.5	312.90
4381	Rhinolophus convexus	307205567	307.21
4382	Eptesicus platyops	306342214.3	306.34
4383	Petrogale inornata	306022180.6	306.02
4384	Massoutiera mzabi	305824265.3	305.82

4385	Dendrolagus spadix	304677721.9	304.68
4386	Lycalopex fulvipes	304633939.4	304.63
4387	Leontopithecus chrysomelas	303784982.8	303.78
4388	Myotis oreias	301290463.4	301.29
4389	Eptesicus japonensis	300500060.2	300.50
4390	Pipistrellus aero	300262511.7	300.26
4391	Capra ibex	300181529.7	300.18
4392	Ctenomys pontifex	299757302.7	299.76
4393	Pseudoryx nghetinhensis	298238932.8	298.24
4394	Spalax arenarius	297855126.9	297.86
4395	Urocitellus parryii	297667636.7	297.67
4396	Isothrix orinoci	296720419.6	296.72
4397	Pongo tapanuliensis	294031323.5	294.03
4398	Apomys zambalensis	291389741.2	291.39
4399	Pteropus melanopogon	289465042.8	289.47
4400	Coendou roosmalenorum	287715587.6	287.72
4401	Ctenomys perrensi	286956804.3	286.96
4402	Hypsugo bemainty	284316818.8	284.32
4403	Laephotis namibensis	283081677.4	283.08
4404	Submyotodon latirostris	281141155.4	281.14
4405	Batomys dentatus	280600390.2	280.60
4406	Eumops wilsoni	280218610.6	280.22
4407	Mandrillus leucophaeus	277940900.6	277.94
4408	Lonchophylla pattoni	276625623.9	276.63
4409	Niviventer brahma	276154499.1	276.15
4410	Cacajao calvus	274903242.1	274.90
4411	Neotoma palatina	273779870.7	273.78
4412	Plecturocebus vieirai	273558699.4	273.56
4413	Vespadelus baverstocki	271591277.9	271.59
4414	Eulemur collaris	271052839.2	271.05
4415	Akodon philipmyersi	270885370.8	270.89
4416	Ctenomys porteousi	267147282	267.15
4417	Mico emiliae	266474158.5	266.47
4418	Nomascus siki	265329933.2	265.33
4419	Ctenomys sociabilis	264769363.9	264.77
4420	Murina beelzebub	264508728.4	264.51
4421	Thylogale calabyi	264279014	264.28
4422	Lepilemur petteri	264136279.1	264.14
4423	Hipposideros rotalis	262839067.2	262.84
4424	Scolomys ucayalensis	261808761.7	261.81
4425	Hyosciurus ileile	261378094.5	261.38

4426	Nyctophilus heran	261224189.7	261.22
4427	Cryptotis aroensis	260592638.2	260.59
4428	Lepilemur microdon	259753262.5	259.75
4429	Cryptotis venezuelensis	256732211.5	256.73
4430	Mops niangarae	255763784.5	255.76
4431	Xenomys nelsoni	254811011.9	254.81
4432	Musseromys gulantang	253553332.9	253.55
4433	Sminthopsis dolichura	253464721	253.46
4434	Kadarsanomys sodyi	250848478	250.85
4435	Suncus aequatorius	249612206.6	249.61
4436	Rattus feliceus	248188717.6	248.19
4437	Myosorex geata	243988038.8	243.99
4438	Chaetodipus arenarius	242386334	242.39
4439	Sus cebifrons	241943526.9	241.94
4440	Echimys vieirai	241207450.4	241.21
4441	Bos mutus	239607652.3	239.61
4442	Proechimys mincae	238248357.5	238.25
4443	Plecturocebus pallescens	237335871.7	237.34
4444	Chiroderma vizottoi	237007714.7	237.01
4445	Murina eleryi	235206289.6	235.21
4446	Rhinolophus belligerator	235060230.3	235.06
4447	Microcavia shiptoni	234109777.1	234.11
4448	Syconycteris hobbit	233904803.7	233.90
4449	Sorex neomexicanus	233725619.1	233.73
4450	Reithrodontomys bakeri	231810442.4	231.81
4451	Megadontomys thomasi	231351593.1	231.35
4452	Crocidura mdumai	230992995	230.99
4453	Euneomys mordax	230807015.2	230.81
4454	Lemmus sibiricus	230446455.6	230.45
4455	Peromyscus eva	227465515.8	227.47
4456	Cryptotis phillipsii	226542883	226.54
4457	Lophuromys roseveari	225932509.4	225.93
4458	Hippocamelus bisulcus	225533908	225.53
4459	Ctenomys conoveri	224946203.8	224.95
4460	Limnomys bryophilus	224071274.6	224.07
4461	Tarsomys echinatus	223570690.5	223.57
4462	Murina lorelieae	222707928.3	222.71
4463	Punomys kofordi	222339947.4	222.34
4464	Geomys texensis	222267516.7	222.27
4465	Neodon forresti	221377627.4	221.38
4466	Gerbillus hesperinus	221197462.2	221.20

4467	Capra walie	220845875.5	220.85
4468	Amblysomus marleyi	220615409.3	220.62
4469	Falsistrellus mackenziei	219331989.7	219.33
4470	Ctenomys latro	216911630.8	216.91
4471	Pseudomys oralis	216122385.1	216.12
4472	Crocidura negrina	214152660.4	214.15
4473	Soricomys montanus	213277029.6	213.28
4474	Isthmomys flavidus	213167954.8	213.17
4475	Hapalemur occidentalis	212588908.4	212.59
4476	Tarsius wallacei	212410976.2	212.41
4477	Leontocebus fuscicollis	211518414.1	211.52
4478	Tylonycteris pygmaeus	209723923.5	209.72
4479	Urocitellus nancyae	208881511.2	208.88
4480	Kobus megaceros	208755656.5	208.76
4481	Hipposideros alongensis	207389862.6	207.39
4482	Acomys airensis	206786346.3	206.79
4483	Sminthopsis macroura	206616504.2	206.62
4484	Reithrodontomys paradoxus	203971434	203.97
4485	Paratriaenops auritus	202562830.1	202.56
4486	Murina shuipuensis	201690912.9	201.69
4487	Niviventer hinpoon	201505223.5	201.51
4488	Mungotictis decemlineata	201096305.9	201.10
4489	Avahi ramanantsoavanai	199354840.2	199.35
4490	Lophuromys menageshae	197390698.8	197.39
4491	Sundasciurus davensis	193686602.6	193.69
4492	Atopogale cubana	193597852.2	193.60
4493	Petrogale assimilis	192842765.2	192.84
4494	Rhipidomys gardneri	191290198.9	191.29
4495	Chaetodipus ammophilus	190328283.5	190.33
4496	Ctenomys bonettoi	189898641.7	189.90
4497	Cynomys parvidens	188763674.4	188.76
4498	Cryptotis lacertosus	187082560.1	187.08
4499	Crocidura gathornei	187075748.9	187.08
4500	Geomys tropicalis	186129256.4	186.13
4501	Alouatta nigerrima	185877826	185.88
4502	Salanoia concolor	184623887.6	184.62
4503	Proechimys pattoni	183679048.1	183.68
4504	Chodsigoa caovansunga	183178489.1	183.18
4505	Neacomys musseri	182592123.4	182.59
4506	Dendromus ruppi	182215893.5	182.22
4507	Rhipidomys ipukensis	179949434.2	179.95

4508	Pithecia rylandsi	179877229.2	179.88
4509	Tateomys rhinogradoides	177719093.1	177.72
4510	Cacajao melanocephalus	177206108.2	177.21
4511	Niviventer culturatus	176789726.8	176.79
4512	Cryptotis perijensis	176558627	176.56
4513	Strigocuscus pelengensis	176506081.8	176.51
4514	Mus vulcani	175660259	175.66
4515	Microcebus rufus	175127054.4	175.13
4516	Rattus koopmani	174296108.2	174.30
4517	Rattus pelurus	174296108.2	174.30
4518	Tarsius pelengensis	174293355.2	174.29
4519	Cynomys mexicanus	173773745.5	173.77
4520	Neacomys dubosti	172256470.2	172.26
4521	Setirostris eleryi	171622255.1	171.62
4522	Otomys jacksoni	171517319.8	171.52
4523	Phyllomys unicolor	171227040.5	171.23
4524	Chodsigoa sodalis	171095440.4	171.10
4525	Abrocoma shistacea	169398977.8	169.40
4526	Myotis cobanensis	167300842.6	167.30
4527	Chiropodomys calamianensis	166661762.6	166.66
4528	Hystrix pumila	166661762.6	166.66
4529	Maxomys panglima	166661762.6	166.66
4530	Manis culionensis	166656940	166.66
4531	Mydaus marchei	166656275.9	166.66
4532	Acerodon leucotis	166654167.8	166.65
4533	Tupaia palawanensis	166653678.1	166.65
4534	Murina chrysochaetes	166386399.2	166.39
4535	Hylopetes nigripes	165518696.2	165.52
4536	Crocidura palawanensis	165510611.7	165.51
4537	Grammomys gigas	165462343.3	165.46
4538	Hypsugo joffrei	163328200	163.33
4539	Microcebus bongolavensis	161851352.3	161.85
4540	Philander deltae	160039713.1	160.04
4541	Neotamias bulleri	159284234.8	159.28
4542	Rhinolophus silvestris	157444914.8	157.44
4543	Callospermophilus madrensis	156510618.2	156.51
4544	Kerivoula krauensis	156028348.7	156.03
4545	Phyllonycteris aphylla	154786023	154.79
4546	Microtus miurus	153178878.2	153.18
4547	Cryptotis celaque	152727080.4	152.73
4548	Vandeleuria nolthenii	152602235	152.60

4549	Sicista caucasica	149512844.6	149.51
4550	Rattus timorensis	148963181.9	148.96
4551	Apomys iridensis	148893328.7	148.89
4552	Sundasciurus juvencus	147658180.9	147.66
4553	Urocitellus washingtoni	146978464.1	146.98
4554	Petromyscus barbouri	146926061.9	146.93
4555	Phaner furcifer	146739121.7	146.74
4556	Eulemur albifrons	146704958.5	146.70
4557	Gerbillurus setzeri	146367829.8	146.37
4558	Heterogeomys dariensis	146284680.1	146.28
4559	Apomys sierrae	145844956.1	145.84
4560	Pseudohydromys berniceae	145132447.4	145.13
4561	Lonchorhina marinkellei	144578996	144.58
4562	Rattus baluensis	143521528.2	143.52
4563	Leontocebus illigeri	143515704.9	143.52
4564	Petinomys hageni	143210065	143.21
4565	Myotis hermani	142866242.1	142.87
4566	Chiruromys forbesi	142311590	142.31
4567	Ctenomys haigi	141923622.4	141.92
4568	Avahi peyrierasi	141129541.1	141.13
4569	Acomys seurati	140323297.4	140.32
4570	Sus bucculentus	140025985.8	140.03
4571	Musseromys beneficus	139171632.3	139.17
4572	Otomys barbouri	138877263.6	138.88
4573	Plecturocebus bernhardi	138300630.9	138.30
4574	Vespadelus finlaysoni	138040594.1	138.04
4575	Suncus dayi	136425720.3	136.43
4576	Amblysomus robustus	136316700.8	136.32
4577	Maxomys dollmani	135868648.9	135.87
4578	Cercocebus chrysogaster	135364845.1	135.36
4579	Marmota camtschatica	134990961.8	134.99
4580	Ctenomys coyhaiquensis	134380752.7	134.38
4581	Taeromys punicans	133773278.8	133.77
4582	Catagonus wagneri	133344686.7	133.34
4583	Crocidura ansellorum	133063188.1	133.06
4584	Pithecheir melanurus	132569705	132.57
4585	Myotis diminutus	132302514.4	132.30
4586	Lophuromys sabunii	131953749.3	131.95
4587	Lonchophylla fornicata	130257313.6	130.26
4588	Lepilemur mustelinus	129052609.1	129.05
4589	Eulemur sanfordi	128778418.2	128.78

4590	Mallomys gunung	128254937.7	128.25
4591	Epomophorus anselli	127638826.4	127.64
4592	Dendroprionomys rousseloti	127579496.7	127.58
4593	Apomys sacobianus	127420256.6	127.42
4594	Microdipodops pallidus	126973657.6	126.97
4595	Dipodomys elator	126442790.3	126.44
4596	Lasiurus castaneus	124358476.9	124.36
4597	Paramelomys gressitti	123693207.6	123.69
4598	Rattus korinchi	123225003.7	123.23
4599	Sorex camtschatica	122908147.1	122.91
4600	Mirza zaza	122172766.2	122.17
4601	Marmosops creightoni	121965536.8	121.97
4602	Calomys tocantinsi	121324284.2	121.32
4603	Dama mesopotamica	120252495.2	120.25
4604	Sorex tenellus	120043671.3	120.04
4605	Peromyscus polius	119696404.4	119.70
4606	Coendou speratus	119611763.2	119.61
4607	Otomops johnstonei	119292902.3	119.29
4608	Reithrodontomys musseri	116386600.2	116.39
4609	Plecturocebus parecis	115935145.2	115.94
4610	Reithrodontomys raviventris	115645114.5	115.65
4611	Peromyscus melanurus	115622894.1	115.62
4612	Propithecus diadema	115553915.3	115.55
4613	Lophuromys chercherensis	114709702.5	114.71
4614	Juscelinomys huanchacae	114539720.3	114.54
4615	Sus ahoenobarbus	114215098	114.22
4616	Epomophorus grandis	113684183.6	113.68
4617	Crateromys heaneyi	113683290.9	113.68
4618	Hylopetes winstoni	113430540.1	113.43
4619	Sylvisorex silvanorum	111067904.2	111.07
4620	Sminthopsis granulipes	110618508.1	110.62
4621	Pithecia pissinattii	110523225.1	110.52
4622	Cryptotis cavatorculus	110160069.7	110.16
4623	Plecturocebus hoffmannsi	110125182	110.13
4624	Rattus mollicomulus	110059600.9	110.06
4625	Otomys zinki	109943957.5	109.94
4626	Pteropus intermedius	108209264.9	108.21
4627	Ctenomys argentinus	107445944.1	107.45
4628	Biswamoyopterus biswasi	106638862.1	106.64
4629	Petromyscus shortridgei	106471541.6	106.47
4630	Rhipidomys wetzeli	105762575.7	105.76

4631	Lophuromys cinereus	105351761.3	105.35
4632	Mus fragilicauda	105172471.8	105.17
4633	Crocidura baluensis	104978832.9	104.98
4634	Saguinus inustus	104728828.6	104.73
4635	Pithecia vanzolinii	104521255.7	104.52
4636	Meriones arimalius	104097204.2	104.10
4637	Ctenomys bergi	104017691.8	104.02
4638	Abrothrix sanborni	103999208.5	104.00
4639	Hybomys basilii	103731282.2	103.73
4640	Lonchophylla chocoana	103479450	103.48
4641	Fukomys kafuensis	103268917.7	103.27
4642	Eulemur coronatus	103093080.2	103.09
4643	Dipodomys gravipes	102877041.9	102.88
4644	Pseudomys albocinereus	102820507.4	102.82
4645	Pseudocheirus occidentalis	102023738.8	102.02
4646	Coendou baturitensis	101947765.8	101.95
4647	Lepilemur seali	101571690.4	101.57
4648	Sus oliveri	99926194.29	99.93
4649	Mystacina tuberculata	99799537.5	99.80
4650	Lophuromys makundii	99201081.36	99.20
4651	Rattus blangorum	99060877.86	99.06
4652	Fukomys vandewoestijneae	98995633.67	99.00
4653	Bunomys coelestis	98752433.19	98.75
4654	Felovia vae	98539804.97	98.54
4655	Melanomys zunigae	96539088.68	96.54
4656	Hipposideros coronatus	96036200.63	96.04
4657	Catopuma badia	94354356.67	94.35
4658	Tamiasciurus mearnsi	94316715.21	94.32
4659	Hypsugo lanzai	93989590.42	93.99
4660	Crocidura zimmermanni	93688888.55	93.69
4661	Calcochloris tytonis	92757637.36	92.76
4662	Macroscelides flavicaudatus	91350829.08	91.35
4663	Crocidura lwiroensis	90361856.42	90.36
4664	Indri indri	90284489.61	90.28
4665	Taphozous georgianus	88774954.35	88.77
4666	Rattus mindorensis	88527035.47	88.53
4667	Plecturocebus grovesi	88469867.03	88.47
4668	Macaca munzala	88390022.92	88.39
4669	Plecturocebus cinerascens	88249634	88.25
4670	Apodemus avicennicus	88065855.91	88.07
4671	Lepilemur dorsalis	88012314.48	88.01

4672	Proechimys hoplomyoides	87155919.19	87.16
4673	Bullimus gamay	86842673.62	86.84
4674	Isothrix negrensis	86840350.43	86.84
4675	Mico leucippe	86341562.61	86.34
4676	Ctenomys brasiliensis	86057742.72	86.06
4677	Pipistrellus permixtus	84658060.21	84.66
4678	Peromyscus mayensis	83979811.67	83.98
4679	Microcebus tavaratra	83892810.8	83.89
4680	Gerbillurus tytonis	83476463.48	83.48
4681	Chacodelphys formosa	83190748.92	83.19
4682	Neotamias canipes	83160598.67	83.16
4683	Cheracebus regulus	82933121.71	82.93
4684	Cryptotis hondurensis	82804656.25	82.80
4685	Alexandromys middendorffii	82177807.88	82.18
4686	Avahi betsileo	82143818.69	82.14
4687	Galea flavidens	81984809.11	81.98
4688	Pseudomys hermannsburgensis	81218410.4	81.22
4689	Salpingotulus michaelis	81160184.06	81.16
4690	Mylomys rex	80570679.37	80.57
4691	Gerbillus grobbeni	79867163.4	79.87
4692	Cryptotis peregrina	79557422.04	79.56
4693	Thylamys pusillus	79362358.42	79.36
4694	Ozimops cobourgianus	78833316.96	78.83
4695	Saguinus melanoleucus	78775932.54	78.78
4696	Nyctophilus arnhemensis	78384704.26	78.38
4697	Rhinonicteris aurantia	78313826.26	78.31
4698	Chaerephon gallagheri	78209004.54	78.21
4699	Petrogale mareeba	77981639.33	77.98
4700	Sylvisorex isabellae	77830054.99	77.83
4701	Heteromys spectabilis	77657722.27	77.66
4702	Crocidura cranbrooki	77323699.08	77.32
4703	Maxomys hylomyoides	77014342.54	77.01
4704	Miniopterus brachytragos	76811551.67	76.81
4705	Chlorocebus dryas	76632222.65	76.63
4706	Ningaui yvonneae	76505556.23	76.51
4707	Graphiurus rupicola	75817382.76	75.82
4708	Apomys magnus	75483992.47	75.48
4709	Pseudomys shortridgei	75473770.23	75.47
4710	Mustela tonkinensis	75238146.69	75.24
4711	Bunomys karokophilus	75183655.18	75.18
4712	Podoxymys roraimae	74941146.21	74.94

4713	Rattus salocco	74829855.24	74.83
4714	Pseudomys nanus	74779094.97	74.78
4715	Pipistrellus westralis	74778894.81	74.78
4716	Lonchophylla dekeyseri	74734911.35	74.73
4717	Oxymycterus hucucha	74678265.15	74.68
4718	Leontocebus nigrifrons	74621495.47	74.62
4719	Osphranter antilopinus	74256459.68	74.26
4720	Nyctophilus daedalus	74250654.3	74.25
4721	Peromyscus schmidlyi	74121884.69	74.12
4722	Antechinomys laniger	73996069.86	74.00
4723	Crocidura wimmeri	73755689.4	73.76
4724	Rhogeessa menchuae	73752693.07	73.75
4725	Rattus colletti	73693188.02	73.69
4726	Taphozous kapalgensis	73693086.45	73.69
4727	Hipposideros stenotis	73693083.73	73.69
4728	Nyctophilus walkeri	73693083.73	73.69
4729	Vespadelus caurinus	73693064.52	73.69
4730	Conilurus penicillatus	73693042.49	73.69
4731	Pipistrellus adamsi	73692728.97	73.69
4732	Rattus montanus	73595503.31	73.60
4733	Mico humeralifer	73467884.25	73.47
4734	Rhipidomys macconnelli	73138748.71	73.14
4735	Prosciurillus abstrusus	73041418.89	73.04
4736	Sminthopsis gilberti	72862862.18	72.86
4737	Tapecomys primus	72595436.41	72.60
4738	Rhinolophus sakejiensis	71743874.71	71.74
4739	Hybomys badius	71105305.27	71.11
4740	Lepus hainanus	70668944.79	70.67
4741	Urocitellus endemicus	70620359.87	70.62
4742	Ctenomys ibicuiensis	70538194	70.54
4743	Planigale gilesi	70513883.5	70.51
4744	Piliocolobus gordonorum	70315859.51	70.32
4745	Crocidura hilliana	70200170.17	70.20
4746	Hapalemur aureus	70152477.67	70.15
4747	Dicrostonyx torquatus	69718652.33	69.72
4748	Crunomys suncoides	69704471.63	69.70
4749	Hypsiprymnodon moschatus	69386076.4	69.39
4750	Ctenomys pundti	69229738.65	69.23
4751	Ctenomys coludo	68845453.85	68.85
4752	Pseudomys bolami	68699365.16	68.70
4753	Cryptotis endersi	68439517.48	68.44

4754	Hipposideros nequam	68407703.09	68.41
4755	Piliocolobus foai	68231106.74	68.23
4756	Hipposideros inexpectatus	68188089.05	68.19
4757	Santamartamys rufodorsalis	67409317.4	67.41
4758	Nyctimene draconilla	67151312.76	67.15
4759	Setonix brachyurus	67109077.56	67.11
4760	Crocidura grandis	66970880.73	66.97
4761	Prionomys batesi	66695539.4	66.70
4762	Rhinopoma hadramauticum	66594492.99	66.59
4763	Proechimys oconnelli	66573839.82	66.57
4764	Myosorex zinki	66569545.25	66.57
4765	Phyllotis osgoodi	66369491.57	66.37
4766	Ctenomys emilianus	66248785.88	66.25
4767	Peromyscus melanocarpus	66108967.89	66.11
4768	Congosorex verheyeni	65933315.5	65.93
4769	Lophostoma kalkoae	65809234.66	65.81
4770	Myotis planiceps	65597898.77	65.60
4771	Thyroptera devivoi	65542542.47	65.54
4772	Congosorex polli	64729094.12	64.73
4773	Peromyscus ochraventer	63898169.14	63.90
4774	Eulemur macaco	63896331.1	63.90
4775	Coccymys kirrhos	63874504.92	63.87
4776	Lemmus trimucronatus	63771579.41	63.77
4777	Laonastes aenigmamus	63617208.9	63.62
4778	Brachytarsomys villosa	63131598.67	63.13
4779	Notomys alexis	62783905.97	62.78
4780	Mesomys leniceps	62642966.48	62.64
4781	Dipodomys ingens	61968811.3	61.97
4782	Pelomys isseli	61769277.78	61.77
4783	Phascogale calura	61308928.91	61.31
4784	Anthops ornatus	60978389.82	60.98
4785	Dobsonia inermis	60978389.82	60.98
4786	Hipposideros dinops	60978389.82	60.98
4787	Melomys bougainville	60978389.82	60.98
4788	Melonycteris woodfordi	60978389.82	60.98
4789	Pteralopex anceps	60978389.82	60.98
4790	Pteropus rayneri	60978389.82	60.98
4791	Solomys ponceleti	60978389.82	60.98
4792	Solomys salebrosus	60978389.82	60.98
4793	Pogonomelomys brassi	60959857.69	60.96
4794	Cheirogaleus grovesi	60197816.94	60.20

4795	Lonchophylla bokermanni	60113733.67	60.11
4796	Gerbillus brockmani	59761680.46	59.76
4797	Hylomys megalotis	58147707.93	58.15
4798	Margaretamys christinae	58109275.32	58.11
4799	Kerodon acrobata	57921541.78	57.92
4800	Eliurus carletoni	55961980.93	55.96
4801	Rattus ranjiniae	55873444.18	55.87
4802	Euneomys chinchilloides	55372777.03	55.37
4803	Macaca leucogenys	55216319.01	55.22
4804	Sorex kozlovi	54525948.15	54.53
4805	Pteralopex flanneryi	52736413.04	52.74
4806	Dendrolagus lumholtzi	51914130.75	51.91
4807	Pteropus mahaganus	51742471.92	51.74
4808	Chodsigoa parva	51454916.1	51.45
4809	Phaner electromontis	50993521.33	50.99
4810	Crocidura umbra	50949185.56	50.95
4811	Rhogeessa mira	50809432.45	50.81
4812	Nesomys lambertoni	50486329.91	50.49
4813	Hylopetes bartelsi	49954336.23	49.95
4814	Hylaeamys acritus	49913432.41	49.91
4815	Rattus hoogerwerfi	49426522.9	49.43
4816	Sylvilagus robustus	49280261.07	49.28
4817	Sicista kazbegica	48525043.17	48.53
4818	Plecturocebus caligatus	48489471.02	48.49
4819	Dorcopsulus macleayi	48392697.87	48.39
4820	Eulemur cinereiceps	47770340.84	47.77
4821	Glauconycteris gleni	47209819.94	47.21
4822	Lamottemys okuensis	47180768.98	47.18
4823	Lepilemur ankaranensis	46562408.47	46.56
4824	Hylomyscus grandis	45899891.56	45.90
4825	Tylomys bullaris	45443045.9	45.44
4826	Mico schneideri	45442916.86	45.44
4827	Cheirogaleus shethi	45429118.36	45.43
4828	Rhinolophus hilli	45079447.48	45.08
4829	Paucidentomys vermidax	44973268.8	44.97
4830	Notiosorex villai	44431194.63	44.43
4831	Sylvisorex morio	44001241.56	44.00
4832	Lemniscomys mittendorfi	43775536.87	43.78
4833	Volemys musseri	43558154.3	43.56
4834	Lophuromys dieterleni	43239317.92	43.24
4835	Apomys aurorae	41964671.46	41.96

4836	Neacomys minutus	41908153.92	41.91
4837	Lepilemur fleuretae	41885566.62	41.89
4838	Cryptotis oreoryctes	41864464.48	41.86
4839	Myoictis wavicus	41843774.65	41.84
4840	Petaurista mechukaensis	41626432.07	41.63
4841	Gracilimus radix	41589069.12	41.59
4842	Pipistrellus minahassae	41373474.44	41.37
4843	Bunomys torajae	41198461.99	41.20
4844	Marmosops handleyi	40431975.47	40.43
4845	Otomops madagascariensis	40327984.46	40.33
4846	Ovis nivicola	40264848.1	40.26
4847	Sommeromys macrorhinos	40161144.99	40.16
4848	Cerradomys marinhus	40046022.27	40.05
4849	Mus fernandoni	39917715.26	39.92
4850	Elephantulus pilicaudus	39695897.3	39.70
4851	Phyllomys kerri	39570517.61	39.57
4852	Leggadina forresti	39010866.63	39.01
4853	Rattus villosissimus	39010866.63	39.01
4854	Lonchorhina fernandezi	38555917.37	38.56
4855	Petrogale lateralis	38226446.93	38.23
4856	Cebus castaneus	37971391.63	37.97
4857	Rattus omichlodes	37616824.84	37.62
4858	Neoromicia malagasyensis	37107405.35	37.11
4859	Diomys crumpi	37004633.23	37.00
4860	Lepilemur grewcockorum	35947473.08	35.95
4861	Plecturocebus modestus	35905662.55	35.91
4862	Pseudochirops archeri	35223079.38	35.22
4863	Apomys banahao	34145170.16	34.15
4864	Microcebus jonahi	33916688.96	33.92
4865	Microcebus ganzhorni	33907503.65	33.91
4866	Gymnobelideus leadbeateri	33663438.47	33.66
4867	Trinomys mirapitanga	33561119.65	33.56
4868	Nomascus hainanus	32844130.86	32.84
4869	Rhinopithecus brelichi	32699356.76	32.70
4870	Pteropus keyensis	32531331.46	32.53
4871	Myotis stalkeri	32531331.42	32.53
4872	Crocidura musseri	32275696.46	32.28
4873	Notamacropus eugenii	32196953.11	32.20
4874	Nyctophilus shirleyae	32117553.57	32.12
4875	Proechimys echinothrix	32055283.88	32.06
4876	Sylvisorex akaibei	31958523.2	31.96

4877	Plecturocebus urubambensis	31516651.51	31.52
4878	Prolemur simus	30918073.17	30.92
4879	Otomys burtoni	30416153.81	30.42
4880	Bathyergus janetta	30098795.15	30.10
4881	Crocidura eisentrauti	30054113.8	30.05
4882	Microcebus simmonsi	30021794.42	30.02
4883	Microcebus tanosi	29972030.56	29.97
4884	Peromyscus hooperi	29781875.21	29.78
4885	Rhynchomys banahao	29687846.09	29.69
4886	Calomyscus tsolovi	29535244.59	29.54
4887	Saguinus martinsi	29517845.77	29.52
4888	Pteropus pohlei	29087413.9	29.09
4889	Emballonura serii	29054226.35	29.05
4890	Neoromicia robertsi	28996991.9	29.00
4891	Lepilemur betsileo	28763015.42	28.76
4892	Crocidura longipes	28554621.22	28.55
4893	Otomys dartmouthi	28208083.86	28.21
4894	Cercocebus sanjei	28045420.35	28.05
4895	Batomys uragon	28028704.38	28.03
4896	Hemibelideus lemuroides	27992180.74	27.99
4897	Pseudochirulus herbertensis	27992180.74	27.99
4898	Myosorex bururiensis	27345184.97	27.35
4899	Spalax antiquus	27254630.6	27.25
4900	Crocidura arispa	27212477.24	27.21
4901	Microperoryctes papuensis	26956784.53	26.96
4902	Petaurista mishmiensis	26568850.82	26.57
4903	Pseudoberylmys muongbangensis	26145550.93	26.15
4904	Plecturocebus baptista	26141902.52	26.14
4905	Tateomys macrocercus	25746263.12	25.75
4906	Cryptotis mccarthyi	25468478.49	25.47
4907	Apomys camiguinensis	25394855.7	25.39
4908	Chaetocauda sichuanensis	25183313.67	25.18
4909	Pseudohydromys occidentalis	25164939.69	25.16
4910	Lophuromys eisentrauti	24808461.95	24.81
4911	Dendrolagus ursinus	24799111.38	24.80
4912	Bison bison	24337762.95	24.34
4913	Orthogeomys cuniculus	24121749	24.12
4914	Cryptotis griseoventris	24030062	24.03
4915	Varecia rubra	24021846.64	24.02
4916	Rhinolophus ziama	23926130.72	23.93
4917	Spermophilus musicus	23765100.33	23.77

4918	Tylomys tumbalensis	23731564.28	23.73
4919	Monodelphis unistriata	23543787.5	23.54
4920	Dendrolagus matschiei	23367190.45	23.37
4921	Mico chrysoleucos	23187951.54	23.19
4922	Avahi meridionalis	23106811.19	23.11
4923	Chodsigoa salenskii	22764444.35	22.76
4924	Murexia rothschildi	22690347.1	22.69
4925	Melomys cooperae	22569760.41	22.57
4926	Rousettus linduensis	22549466.92	22.55
4927	Crocidura nana	22501152.86	22.50
4928	Habromys schmidlyi	22445822.05	22.45
4929	Neotamias panamintinus	22368066.8	22.37
4930	Microtus umbrosus	22233834.98	22.23
4931	Haeromys margarettae	22189923.42	22.19
4932	Sturnira mistratensis	22022535.48	22.02
4933	Suncus mertensi	21952088.46	21.95
4934	Sicista loriger	21723877.52	21.72
4935	Ochotona collaris	21510172.73	21.51
4936	Rhinolophus perditus	21195002.16	21.20
4937	Hipposideros turpis	21193798.9	21.19
4938	Trachypithecus poliocephalus	21153076.95	21.15
4939	Cheracebus torquatus	20780116.39	20.78
4940	Microgale nasoloi	20696271.68	20.70
4941	Mesocapromys nanus	20411124.85	20.41
4942	Planigale ingrami	20369261.66	20.37
4943	Pseudomys johnsoni	20369261.66	20.37
4944	Pteronotus paraguanensis	20092276.68	20.09
4945	Microgale longicaudata	20062515.56	20.06
4946	Propithecus tattersalli	20059972.91	20.06
4947	Alticola lemminus	19997468.77	20.00
4948	Melogale everetti	19871441.84	19.87
4949	Petrogale purpureicollis	19822062.01	19.82
4950	Galidictis grandidieri	19734194.46	19.73
4951	Crocidura virgata	19464797.72	19.46
4952	Pseudomys occidentalis	19430078.59	19.43
4953	Myosorex jejei	19418016.08	19.42
4954	Pseudomys apodemoides	19260532.05	19.26
4955	Neodon linzhiensis	18971591.54	18.97
4956	Cheirogaleus sibreei	18541536.87	18.54
4957	Murina ryukyuana	18492848	18.49
4958	Ctenomys roigi	18420552.1	18.42

4959	Sylvilagus varynaensis	17990477.4	17.99
4960	Brassomys albidens	17717871.91	17.72
4961	Rhinolophus montanus	17702107.26	17.70
4962	Voalavo gymnocaudus	17525679.7	17.53
4963	Micronycteris giovanniae	17490221.99	17.49
4964	Sorex stizodon	17276089.87	17.28
4965	Lynx pardinus	17248286.47	17.25
4966	Cercocebus galeritus	16919352.93	16.92
4967	Suncus ater	16823246.2	16.82
4968	Ovis dalli	16709369.64	16.71
4969	Phyllomys brasiliensis	16655003.57	16.66
4970	Microgale dryas	16586767.17	16.59
4971	Peromyscus bullatus	16580349.37	16.58
4972	Petaurillus emiliae	16530920.95	16.53
4973	Microperoryctes murina	16497368.1	16.50
4974	Heterogeomys lanius	16472511.87	16.47
4975	Cryptomys mahali	16300497.72	16.30
4976	Surdisorex schlitteri	16268740.07	16.27
4977	Microgale jobihely	16266964.35	16.27
4978	Propithecus edwardsi	16181317.88	16.18
4979	Microcebus mamiratra	16112602.77	16.11
4980	Rhinolophus mitratus	16065406.46	16.07
4981	Microcebus margotmarshae	15925348.67	15.93
4982	Gerbillus lowei	15881738.9	15.88
4983	Calyptophractus retusus	15528711.31	15.53
4984	Pithecia albicans	15312966.3	15.31
4985	Taterillus tranieri	15024549.83	15.02
4986	Paragalago rondoensis	14908959.47	14.91
4987	Neotoma angustapalata	14548278.78	14.55
4988	Rhagomys longilingua	14392633.53	14.39
4989	Cremnomys elvira	14265390.02	14.27
4990	Podogymnura aureospinula	14225079.08	14.23
4991	Lepilemur septentrionalis	14224638.17	14.22
4992	Lepilemur milanoii	13592567.37	13.59
4993	Piliocolobus rufomitratus	13550618.13	13.55
4994	Neotoma nelsoni	13438920.55	13.44
4995	Chrotomys gonzalesi	13159509.21	13.16
4996	Microtus elbeyli	13023748.85	13.02
4997	Cheirogaleus thomasi	13015261.05	13.02
4998	Onychogalea fraenata	12935949.17	12.94
4999	Ningaui ridei	12831071.89	12.83

5000	Antechinus adustus	12800332.6	12.80
5001	Antechinus godmani	12800332.6	12.80
5002	Sminthopsis youngsoni	12800278.69	12.80
5003	Crateromys australis	12570920.82	12.57
5004	Plecturocebus miltoni	12251951.77	12.25
5005	Crocidura armenica	12240721.67	12.24
5006	Avahi cleesei	12186781.36	12.19
5007	Allocebus trichotis	11415105.07	11.42
5008	Pseudomys fumeus	11320832.11	11.32
5009	Batomys russatus	11263701.41	11.26
5010	Miniopterus ambohitrensis	11251970.96	11.25
5011	Sorex sclateri	11098187.76	11.10
5012	Crocidura polia	10921060.18	10.92
5013	Thomasomys onkiro	10902489.38	10.90
5014	Anonymomys mindorensis	10902423.12	10.90
5015	Rhynchomys isarogensis	10742911.52	10.74
5016	Lepilemur tymerlachsoni	10686355.05	10.69
5017	Apomys gracilirostris	10579659.84	10.58
5018	Lonchophylla orcesi	10482236.97	10.48
5019	Lophuromys stanleyi	10356136.24	10.36
5020	Tokudaia muenninki	10251972.84	10.25
5021	Sundasciurus steerii	10146646.31	10.15
5022	Plecturocebus aureipalatii	9869751.043	9.87
5023	Cryptochloris zyli	9816158.173	9.82
5024	Sorex lyelli	9729463.675	9.73
5025	Carollia monohernandezi	9592651.605	9.59
5026	Gerbillus percivali	9590571.404	9.59
5027	Myosorex eisentrauti	9407845.512	9.41
5028	Crocidura pergrisea	9357928.297	9.36
5029	Neotamias alpinus	9274400.86	9.27
5030	Myosorex kabogoensis	9272429.636	9.27
5031	Callibella humilis	8926134.506	8.93
5032	Thallomys shortridgei	8822737.032	8.82
5033	Thyroptera wynneae	8726648.883	8.73
5034	Dasycercus blythi	8697125.902	8.70
5035	Sminthopsis longicaudata	8697125.902	8.70
5036	Sminthopsis ooldea	8691879.428	8.69
5037	Microcebus danfossi	8647649.345	8.65
5038	Lepilemur randrianasoloi	8405776.973	8.41
5039	Microgale monticola	8296366.102	8.30
5040	Taphozous hilli	8179538.879	8.18

5041	Glauconycteris kenyacola	8121254.622	8.12
5042	Tonkinomys daovantieni	8074730.809	8.07
5043	Millardia kondana	8065974.677	8.07
5044	Pseudomys australis	8043526.891	8.04
5045	Lepilemur hubbardorum	7853459.746	7.85
5046	Myotis peninsularis	7800529.142	7.80
5047	Abrocoma boliviensis	7551388.642	7.55
5048	Petaurus gracilis	7367030.453	7.37
5049	Mogera tokudae	7108381.113	7.11
5050	Apomys minganensis	7101856.648	7.10
5051	Petrogale persephone	6992416.832	6.99
5052	Rungwecebus kipunji	6975408.424	6.98
5053	Hapalemur alaotrensis	6975273.914	6.98
5054	Plecturocebus stephennashi	6960099.28	6.96
5055	Megadontomys cryophilus	6915209.426	6.92
5056	Alexandromys sachalinensis	6910749.516	6.91
5057	Dasykaluta rosamondae	6881245.584	6.88
5058	Ningaui timealeyi	6881245.584	6.88
5059	Pseudantechinus roryi	6881245.584	6.88
5060	Dobsonia chapmani	6732731.213	6.73
5061	Myosorex rumpii	6555112.287	6.56
5062	Graphiurus walterverheyeni	6395018.588	6.40
5063	Myotis morrisi	6326425.764	6.33
5064	Sicista trizona	6325935.588	6.33
5065	Plecturocebus caquetensis	6312333.421	6.31
5066	Crunomys fallax	6271659.138	6.27
5067	Marmota olympus	6119950.734	6.12
5068	Myoictis leucura	6034055.439	6.03
5069	Notoryctes typhlops	5919033.102	5.92
5070	Pseudantechinus macdonnellensis	5919033.102	5.92
5071	Zyzomys pedunculatus	5919033.102	5.92
5072	Trachypithecus laotum	5737134.287	5.74
5073	Spalax istricus	5677378.71	5.68
5074	Archboldomys luzonensis	5558265.295	5.56
5075	Dryomys niethammeri	5515146.161	5.52
5076	Ovibos moschatus	5381083.308	5.38
5077	Lepus arcticus	5254624.628	5.25
5078	Sylvilagus insonus	5254042.123	5.25
5079	Crocidura annamitensis	4996307.123	5.00
5080	Propithecus candidus	4929150.713	4.93
5081	Leimacomys buettneri	4779985.538	4.78

5082	Neotamias palmeri	4668003.696	4.67
5083	Lepilemur jamesorum	4667819.985	4.67
5084	Saxatilomys paulinae	4524919.691	4.52
5085	Lepilemur aeeclis	4386691.025	4.39
5086	Crocidura macowi	4327463.737	4.33
5087	Macrotis lagotis	4296840.537	4.30
5088	Musseromys inopinatus	4231527.656	4.23
5089	Eulemur flavifrons	4091028.024	4.09
5090	Parantechinus apicalis	4080269.82	4.08
5091	Pithecia cazuzai	4041553.111	4.04
5092	Andalgalomys pearsoni	4032392.737	4.03
5093	Tragulus williamsoni	3930982.039	3.93
5094	Thamnomys schoutedeni	3908716.241	3.91
5095	Pithecia hirsuta	3784306.088	3.78
5096	Microcebus jollyae	3705348.955	3.71
5097	Nanger dama	3667921.516	3.67
5098	Paraleptomys rufilatus	3638542.938	3.64
5099	Lepilemur leucopus	3431691.642	3.43
5100	Pseudantechinus woolleyae	3325174.926	3.33
5101	Pithecia isabela	3325105.409	3.33
5102	Hypsugo anthonyi	3105365.352	3.11
5103	Potorous longipes	2922341.849	2.92
5104	Pseudohydromys germani	2845536.672	2.85
5105	Rattus vandeuseni	2845536.672	2.85
5106	Piliocolobus preussi	2826253.845	2.83
5107	Pseudomys chapmani	2807587.903	2.81
5108	Pteropus seychellensis	2737415.384	2.74
5109	Murina fusca	2626557.784	2.63
5110	Murina harpioloides	2568898.706	2.57
5111	Ctenomys goodfellowi	2498795.382	2.50
5112	Dicrostonyx nelsoni	2306322.401	2.31
5113	Lepus othus	2306322.401	2.31
5114	Acerodon humilis	2292553.519	2.29
5115	Melomys caurinus	2292553.519	2.29
5116	Melomys talaudium	2292553.519	2.29
5117	Leontocebus cruzlimai	2273962.569	2.27
5118	Isthmomys pirrensis	2271568.488	2.27
5119	Mico intermedius	2271401.299	2.27
5120	Petrogale rothschildi	2260505.777	2.26
5121	Biswamoyopterus laoensis	2234227.685	2.23
5122	Rattus elaphinus	2213924.623	2.21

5123	Sicista armenica	2128432.661	2.13
5124	Acomys cilicicus	2095868.432	2.10
5125	Romerolagus diazi	2079720.572	2.08
5126	Bettongia tropica	1981144.623	1.98
5127	Taeromys microbullatus	1940243.774	1.94
5128	Eliurus danieli	1932027.831	1.93
5129	Mesomys occultus	1868217.844	1.87
5130	Phyllomys thomasi	1801788.275	1.80
5131	Pithecheirops otion	1728726.985	1.73
5132	Alexandromys kikuchii	1655158.941	1.66
5133	Crocidura hikmiya	1611030.187	1.61
5134	Cacajao hosomi	1602848.444	1.60
5135	Crocidura orii	1424230.261	1.42
5136	Burramys parvus	1400979.432	1.40
5137	Dicrostonyx groenlandicus	1398381.588	1.40
5138	Myotis soror	1323457.555	1.32
5139	Peromyscus winkelmanni	1192596.489	1.19
5140	Styloctenium mindorensis	1188343.42	1.19
5141	Nyctimene cyclotis	1167704.733	1.17
5142	Pseudochirops coronatus	1167704.733	1.17
5143	Pseudochirulus schlegeli	1167704.733	1.17
5144	Myosorex schalleri	1166866.75	1.17
5145	Proechimys kulinae	1162582.228	1.16
5146	Crocidura harenna	1160795.733	1.16
5147	Dendromus vernayi	1146077.461	1.15
5148	Axis calamianensis	1143066.401	1.14
5149	Sundasciurus hoogstraali	1143066.401	1.14
5150	Mico marcai	1142162.839	1.14
5151	Crateromys paulus	1140704.972	1.14
5152	Crocidura sokolovi	1128652.315	1.13
5153	Onychogalea unguifera	1086008.394	1.09
5154	Leontocebus fuscus	1071357.451	1.07
5155	Sminthopsis hirtipes	1036068.83	1.04
5156	Trachypithecus shortridgei	1035720.308	1.04
5157	Tokudaia osimensis	1031353.571	1.03
5158	Microcebus marohita	1020641.558	1.02
5159	Leptomys signatus	1017558.833	1.02
5160	Cryptochloris wintoni	979053.0041	0.98
5161	Myrmecobius fasciatus	950326.3662	0.95
5162	Dicrostonyx hudsonius	939140.7451	0.94
5163	Axis kuhlii	890408.7292	0.89

5164	Eliurus penicillatus	874067.5586	0.87
5165	Myosorex gnoskei	823585.1263	0.82
5166	Gardnerycteris koepckeae	787946.7454	0.79
5167	Hipposideros lamottei	630443.5751	0.63
5168	Pseudomys pilligaensis	618684.9075	0.62
5169	Piliocolobus bouvieri	584166.0529	0.58
5170	Dendrolagus pulcherrimus	582966.3524	0.58
5171	Rattus tawitawiensis	581625.2538	0.58
5172	Mico munduruku	580768.2393	0.58
5173	Neoromicia roseveari	578146.6607	0.58
5174	Trinomys yonenagae	574366.1685	0.57
5175	Glauconycteris machadoi	573014.5005	0.57
5176	Crocidura zimmeri	570616.118	0.57
5177	Antechinus bellus	570304.5836	0.57
5178	Cuscomys oblativa	570262.894	0.57
5179	Microcebus arnholdi	568056.3003	0.57
5180	Petrogale godmani	563373.113	0.56
5181	Dendrolagus bennettianus	562093.744	0.56
5182	Lepus flavigularis	561068.1903	0.56
5183	Crocidura dhofarensis	560356.4529	0.56
5184	Microtus oaxacensis	557882.044	0.56
5185	Mico nigriceps	550345.4726	0.55
5186	Sturnira nana	545681.001	0.55
5187	Leporillus apicalis	517587.0228	0.52
5188	Abrocoma budini	513232.9855	0.51
5189	Fukomys ilariae	505543.5994	0.51
5190	Lasiorhinus latifrons	488794.5305	0.49
5191	Alticola olchonensis	485496.7726	0.49
5192	Rhynchocyon udzungwensis	467082.0529	0.47
5193	Tympanoctomys kirchnerorum	426968.6158	0.43
5194	Fukomys livingstoni	407560.0041	0.41
5195	Propithecus perrieri	351737.521	0.35
5196	Rhinolophus kahuzi	322060.5641	0.32
5197	Diplothrix legata	321796.0601	0.32
5198	Avahi unicolor	294274.7076	0.29
5199	Eliurus ellermani	283738.0968	0.28
5200	Surdisorex polulus	270218.3596	0.27
5201	Sorex portenkoi	252236.8285	0.25
5202	Sorex ugyunak	217526.8861	0.22
5203	Crocidura mindorus	206283.2387	0.21
5204	Tarsius pumilus	192446.8578	0.19

5205	Lepilemur ahmansoni	170882.6263	0.17
5206	Alexandromys evoronensis	69585.0208	0.07
5207	Otomops secundus	65593.1938	0.07
5208	Dendromus kahuziensis	18940.58739	0.02
5209	Crocidura panayensis	15719.19281	0.02
5210	Habromys lepturus	14322.98492	0.01
5211	Trachypithecus barbei	1880.096761	0.00
5212	Crocidura ninoyi	362.268163	0.00

Table S2: The cloven-hoofed animals designated by the International Union for Conservation of Nature (IUCN) as most affected by *Brucella* risk

No.	sci_name	Brucellosis Overlap（M²）	Brucellosis Overlap（km²）
1	Sus scrofa	7.20002E+12	7200021.43
2	Rusa unicolor	2.72912E+12	2729116.897
3	Muntiacus vaginalis	2.27624E+12	2276237.646
4	Capreolus pygargus	2.07018E+12	2070177.658
5	Muntiacus reevesi	1.56399E+12	1563987.445
6	Tragelaphus scriptus	1.47767E+12	1477672.116
7	Sylvicapra grimmia	1.4234E+12	1423395.157
8	Capricornis sumatraensis	1.24804E+12	1248042.83
9	Elaphodus cephalophus	1.18905E+12	1189053.173
10	Kobus ellipsiprymnus	1.08302E+12	1083023.713
11	Phacochoerus africanus	1.05218E+12	1052179.734
12	Odocoileus virginianus	1.00832E+12	1008316.989
13	Gazella subgutturosa	1.00217E+12	1002165.793
14	Axis axis	9.68069E+11	968069.0446
15	Capreolus capreolus	9.14243E+11	914242.5043
16	Moschus berezovskii	8.81048E+11	881047.6009
17	Ourebia ourebi	8.7278E+11	872780.4695
18	Moschus moschiferus	8.64295E+11	864295.3125
19	Potamochoerus larvatus	8.57578E+11	857578.1373
20	Boselaphus tragocamelus	8.23295E+11	823294.6048
21	Oreotragus oreotragus	7.46615E+11	746614.9135
22	Tetracerus quadricornis	7.45011E+11	745010.5579
23	Redunca redunca	7.25679E+11	725678.9517
24	Dama dama	7.17918E+11	717917.9971
25	Syncerus caffer	7.01727E+11	701726.8517
26	Cervus elaphus	6.55839E+11	655839.344
27	Cervus canadensis	6.30502E+11	630502.2134
28	Pecari tajacu	6.23986E+11	623985.7208
29	Ovis ammon	5.7035E+11	570350.1046
30	Pseudois nayaur	5.66866E+11	566865.5748
31	Tragelaphus strepsiceros	5.57554E+11	557553.8463
32	Philantomba monticola	5.35307E+11	535307.4421
33	Alcelaphus buselaphus	5.3061E+11	530609.8022
34	Tragelaphus oryx	5.28244E+11	528243.8165
35	Antilope cervicapra	5.0913E+11	509129.6536
36	Moschus chrysogaster	4.85499E+11	485499.3754
37	Procapra picticaudata	4.62633E+11	462633.3323
38	Cephalophus silvicultor	4.50682E+11	450681.9041

39	Alces alces	4.27019E+11	427018.5341
40	Muntiacus muntjak	3.96946E+11	396946.4581
41	Tragulus kanchil	3.78171E+11	378170.5652
42	Potamochoerus porcus	3.72314E+11	372314.4473
43	Aepyceros melampus	3.66536E+11	366536.446
44	Redunca arundinum	3.62717E+11	362716.8607
45	Tayassu pecari	3.52962E+11	352961.8195
46	Gazella bennettii	3.42147E+11	342147.0416
47	Hippotragus equinus	3.24813E+11	324812.877
48	Cephalophus rufilatus	3.2006E+11	320060.388
49	Mazama americana	3.0682E+11	306819.5935
50	Capra sibirica	3.05076E+11	305076.2112
51	Kobus kob	2.85107E+11	285107.3978
52	Raphicerus campestris	2.64005E+11	264004.637
53	Procapra gutturosa	2.62075E+11	262075.4905
54	Hippotragus niger	2.61127E+11	261127.2306
55	Bos gaurus	2.60976E+11	260975.7639
56	Tragulus napu	2.60958E+11	260957.8385
57	Cervus albirostris	2.37779E+11	237779.1173
58	Raphicerus sharpei	2.10966E+11	210965.9222
59	Hylochoerus meinertzhageni	2.08135E+11	208134.8722
60	Mazama gouazoubira	2.03073E+11	203072.6699
61	Cephalophus dorsalis	1.95491E+11	195491.1234
62	Redunca fulvorufula	1.95435E+11	195434.843
63	Ovis vignei	1.79318E+11	179317.5643
64	Capra aegagrus	1.72321E+11	172321.3575
65	Nesotragus moschatus	1.68027E+11	168027.0264
66	Cephalophus niger	1.64921E+11	164920.8722
67	Madoqua kirkii	1.60057E+11	160056.8412
68	Mazama temama	1.56222E+11	156221.948
69	Tragelaphus imberbis	1.33161E+11	133161.1549
70	Cephalophus nigrifrons	1.33017E+11	133016.5559
71	Cervus nippon	1.3043E+11	130430.2286
72	Connochaetes taurinus	1.27545E+11	127545.1156
73	Odocoileus hemionus	1.17873E+11	117873.2535
74	Hyemoschus aquaticus	1.07098E+11	107097.6707
75	Gazella dorcas	1.06748E+11	106748.3165
76	Cephalophus weynsi	1.04679E+11	104679.2044
77	Tragelaphus eurycerus	1.04226E+11	104226.2964
78	Madoqua guentheri	1.03774E+11	103773.7678
79	Litocranius walleri	1.02036E+11	102035.8108

80	Budorcas taxicolor	97129777073	97129.77707
81	Philantomba maxwellii	96143323584	96143.32358
82	Muntiacus rooseveltorum	93690057107	93690.05711
83	Philantomba walteri	91574707477	91574.70748
84	Gazella marica	89023809972	89023.80997
85	Nanger granti	85573547689	85573.54769
86	Giraffa camelopardalis	85509031755	85509.03175
87	Cephalophus harveyi	84469005342	84469.00534
88	Cephalophus natalensis	83322320081	83322.32008
89	Ovis gmelini	79087277835	79087.27783
90	Madoqua saltiana	77938203628	77938.20363
91	Damaliscus lunatus	74875534758	74875.53476
92	Muntiacus crinifrons	73553845468	73553.84547
93	Capricornis crispus	69858227178	69858.22718
94	Neotragus pygmaeus	69123528102	69123.5281
95	Pantholops hodgsonii	68175204700	68175.2047
96	Mazama nemorivaga	66760829219	66760.82922
97	Eudorcas rufifrons	63499290251	63499.29025
98	Pelea capreolus	61609530966	61609.53097
99	Sus barbatus	61435980961	61435.98096
100	Naemorhedus goral	59030895946	59030.89595
101	Sus celebensis	57717050802	57717.0508
102	Oryx beisa	55723471199	55723.4712
103	Muntiacus atherodes	52467680662	52467.68066
104	Antidorcas marsupialis	49894549839	49894.54984
105	Damaliscus pygargus	46032866633	46032.86663
106	Gazella arabica	45823811231	45823.81123
107	Rangifer tarandus	44323571292	44323.57129
108	Naemorhedus caudatus	42616767667	42616.76767
109	Moschus leucogaster	38286937821	38286.93782
110	Lama guanicoe	37697604219	37697.60422
111	Muntiacus montanus	36697952348	36697.95235
112	Moschus fuscus	36620359526	36620.35953
113	Hippocamelus antisensis	36128366980	36128.36698
114	Muntiacus truongsonensis	35963477703	35963.4777
115	Mazama bororo	34490625091	34490.62509
116	Saiga tatarica	34279504039	34279.50404
117	Pudu mephistophiles	32786821211	32786.82121
118	Vicugna vicugna	29645586067	29645.58607
119	Connochaetes gnou	28873117003	28873.117
120	Eudorcas thomsonii	28206616005	28206.61601

121	Mazama nana	27137340514	27137.34051
122	Cephalophus ogilbyi	26617991453	26617.99145
123	Antilocapra americana	26107559927	26107.55993
124	Phacochoerus aethiopicus	25332266719	25332.26672
125	Rusa marianna	22228410293	22228.41029
126	Neotragus batesi	22087263804	22087.2638
127	Rupicapra rupicapra	20559024968	20559.02497
128	Oryx gazella	18057127653	18057.12765
129	Bos javanicus	15928865727	15928.86573
130	Pudu puda	15762795636	15762.79564
131	Oryx dammah	15103928169	15103.92817
132	Muntiacus putaoensis	14867773480	14867.77348
133	Capra nubiana	14833330817	14833.33082
134	Cephalophus leucogaster	13683906556	13683.90656
135	Nanger soemmerringii	12841841471	12841.84147
136	Muntiacus vuquangensis	12789530628	12789.53063
137	Sus philippensis	11266588349	11266.58835
138	Madoqua piacentinii	10591126000	10591.126
139	Bubalus depressicornis	10537188619	10537.18862
140	Ammotragus lervia	9947102264	9947.102264
141	Gazella cuvieri	9686847937	9686.847937
142	Raphicerus melanotis	9047358984	9047.358984
143	Gazella gazella	8725662533	8725.662533
144	Moschus cupreus	8441386021	8441.386021
145	Mazama rufina	7150675384	7150.675384
146	Bubalus quarlesi	7031511270	7031.51127
147	Rusa timorensis	6983625155	6983.625155
148	Ovis canadensis	6836913766	6836.913766
149	Tragelaphus angasii	6752028889	6752.028889
150	Hemitragus jemlahicus	6431530595	6431.530595
151	Muntiacus gongshanensis	6427394255	6427.394255
152	Capra pyrenaica	6377316507	6377.316507
153	Cervus hanglu	6240861448	6240.861448
154	Gazella spekei	5990722332	5990.722332
155	Moschus anhuiensis	5721836128	5721.836128
156	Mazama chunyi	5253287573	5253.287573
157	Tragelaphus derbianus	4376255528	4376.255528
158	Kobus leche	4337353184	4337.353184
159	Sus verrucosus	4315290970	4315.29097
160	Mazama bricenii	3954552712	3954.552712
161	Dorcatragus megalotis	3837431107	3837.431107

162	Capra falconeri	3742701133	3742.701133
163	Ammodorcas clarkei	3682847054	3682.847054
164	Mazama pandora	3526771782	3526.771782
165	Muntiacus feae	3224271043	3224.271043
166	Okapia johnstoni	2993677036	2993.677036
167	Cephalophus callipygus	2663917588	2663.917588
168	Tragelaphus buxtoni	2306661263	2306.661263
169	Capricornis rubidus	2211858130	2211.85813
170	Gazella leptoceros	1947408995	1947.408995
171	Capra cylindricornis	1918704951	1918.704951
172	Oreamnos americanus	1833409764	1833.409764
173	Arabitragus jayakari	1638588245	1638.588245
174	Eudorcas tilonura	1518165948	1518.165948
175	Capricornis swinhoei	1503046790	1503.04679
176	Cephalophus spadix	1419214869	1419.214869
177	Nilgiritragus hylocrius	1208653574	1208.653574
178	Ozotoceros bezoarticus	1104496234	1104.496234
179	Eudorcas albonotata	972580650	972.58065
180	Cephalophus jentinki	694486266.9	694.4862669
181	Cephalophus zebra	694486266.9	694.4862669
182	Cephalophus adersi	648600311.5	648.6003115
183	Rupicapra pyrenaica	645095232.1	645.0952321
184	Tragulus versicolor	612383937.7	612.3839377
185	Camelus ferus	607091577.6	607.0915776
186	Bison bonasus	576674723.3	576.6747233
187	Bos sauveli	501165587.6	501.1655876
188	Procapra przewalskii	465740145.2	465.7401452
189	Porcula salvania	465279254.3	465.2792543
190	Beatragus hunteri	395717347.1	395.7173471
191	Rusa alfredi	369376268.1	369.3762681
192	Oryx leucoryx	358706194.2	358.7061942
193	Naemorhedus baileyi	343130360.1	343.1303601
194	Capra caucasica	328233905.9	328.2339059
195	Capra ibex	300181529.7	300.1815297
196	Pseudoryx nghetinhensis	298238932.8	298.2389328
197	Sus cebifrons	241943526.9	241.9435269
198	Bos mutus	239607652.3	239.6076523
199	Hippocamelus bisulcus	225533908	225.533908
200	Capra walie	220845875.5	220.8458755
201	Kobus megaceros	208755656.5	208.7556565
202	Sus bucculentus	140025985.8	140.0259858

203	Catagonus wagneri	133344686.7	133.3446867
204	Dama mesopotamica	120252495.2	120.2524952
205	Sus ahoenobarbus	114215098	114.215098
206	Sus oliveri	99926194.29	99.92619429
207	Ovis nivicola	40264848.1	40.2648481
208	Bison bison	24337762.95	24.33776295
209	Ovis dalli	16709369.64	16.70936964
210	Ovibos moschatus	5381083.308	5.381083308
211	Tragulus williamsoni	3930982.039	3.930982039
212	Nanger dama	3667921.516	3.667921516
213	Axis calamianensis	1143066.401	1.143066401
214	Axis kuhlii	890408.7292	0.890408729
